# Altered topology of large-scale structural brain networks in chronic stroke

**DOI:** 10.1093/braincomms/fcz020

**Published:** 2019-10-04

**Authors:** Bastian Cheng, Eckhard Schlemm, Robert Schulz, Marlene Boenstrup, Arnaud Messé, Claus Hilgetag, Christian Gerloff, Götz Thomalla

**Affiliations:** 1 Department of Neurology, University Medical Center Hamburg-Eppendorf, D20246 Hamburg, Germany; 2 Human Cortical Physiology and Neurorehabilitation Section, National Institute of Neurological Disorders and Stroke, National Institutes of Health, Bethesda, MD 20892, USA; 3 Institute of Computational Neuroscience, University Medical Center Hamburg-Eppendorf, Hamburg 20252, Germany

**Keywords:** cerebral ischaemia, plasticity, imaging methodology, axon degeneration

## Abstract

Beyond disruption of neuronal pathways, focal stroke lesions induce structural disintegration of distant, yet connected brain regions via retrograde neuronal degeneration. Stroke lesions alter functional brain connectivity and topology in large-scale brain networks. These changes are associated with the degree of clinical impairment and recovery. In contrast, changes of large scale, structural brain networks after stroke are less well reported. We therefore aimed to analyse the impact of focal lesions on the structural connectome after stroke based on data from diffusion-weighted imaging and probabilistic fibre tracking. In total, 17 patients (mean age 64.5 ± 8.4 years) with upper limb motor deficits in the chronic stage after stroke and 21 healthy participants (mean age 64.9 ± 10.3 years) were included. Clinical deficits were evaluated by grip strength and the upper extremity Fugl-Meyer assessment. We calculated global and local graph theoretical measures to characterize topological changes in the structural connectome. Results from our analysis demonstrated significant alterations of network topology in both ipsi- and contralesional, primarily unaffected, hemispheres after stroke. Global efficiency was significantly lower in stroke connectomes as an indicator of overall reduced capacity for information transfer between distant brain areas. Furthermore, topology of structural connectomes was shifted toward a higher degree of segregation as indicated by significantly higher values of global clustering and modularity. On a level of local network parameters, these effects were most pronounced in a subnetwork of cortico-subcortical brain regions involved in motor control. Structural changes were not significantly associated with clinical measures. We propose that the observed network changes in our patients are best explained by the disruption of inter- and intrahemispheric, long white matter fibre tracts connecting distant brain regions. Our results add novel insights on topological changes of structural large-scale brain networks in the ipsi- and contralesional hemisphere after stroke.

## Teaser

We characterize topological changes in large-scale structural brain networks in 17 patients with chronic stroke. By applying graph theoretical methods, we demonstrate significant disruption of connectome topology being shifted toward a higher degree of segregation and loss of integration analogous to results from a basic network simulation model.

## Graphical Abstract

**Graphical Abstract:**
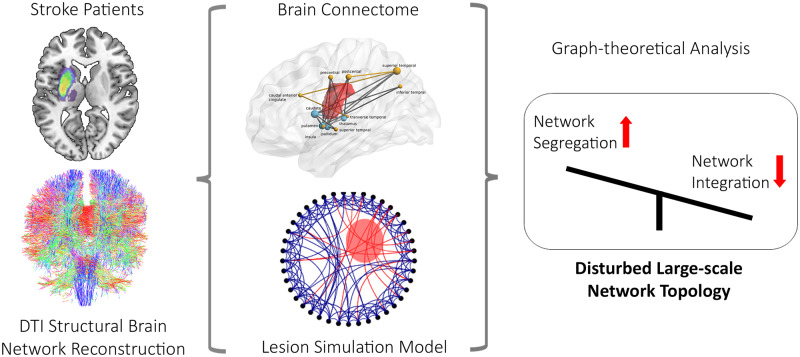

## Introduction

Ischaemic stroke is one of the major causes of adult disability worldwide, often leading to deficits of motor, cognitive or language functions and thus impairments in daily life. In addition to localized disruption of neuronal pathways, stroke lesions induce remote effects on brain function in distant, yet connected brain regions, a phenomenon known as diaschisis (Carrera and Tononi, 2014). In a similar pattern, subcortical stroke lesions have been shown to induce structural degeneration of remote, yet connected white and grey matter, most likely due to retrograde, anterograde as well as transneuronal degeneration (Duering *et al.*, 2015; Cheng *et al.*, 2019).

In recent years, growing evidence supports the understanding of the brain as a complex network of interconnected areas, the structural basis of which can be described by the structural ‘connectome’ (Bullmore and Sporns, 2009). This comprehensive map of neuronal connections reconstructs the brain as a network based on two principle components: nodes, which represent pre-specified cortical areas; and edges, representing the interconnecting white matter tracts. In this abstract form, the connectome can be described by mathematical approaches such as graph theory (Rubinov and Sporns, 2010). By this approach, we gain insight into organizational properties of the brain structure, its configuration and topology. A growing number of studies demonstrate that nodes and edges in the connectome are not organized in a random configuration. Rather, structural connections are arranged in a balanced state that supports functional segregation between different specialized brain areas but also allows for their functional integration (Sporns, 2013). These aspects of network topology can be captured by specific graph theoretical measures such as the global efficiency, clustering and modularity (Rubinov and Sporns, 2010; Aerts *et al.*, 2016).

Focal stroke lesions disrupt the large-scale network of interconnected brain areas distant from the lesion side as a specific form of ‘connectomal’ diaschisis (Carrera and Tononi, 2014). There is evidence for widespread alterations of functional brain connectivity in the human connectome after stroke studied by electroencephalography and functional magnetic resonance imaging (MRI). However studies of structural changes in large scale, whole-brain networks after stroke are still scarce (Carter *et al.*, 2012; Aerts *et al.*, 2016). Specifically, there are few reports on the effect of focal stroke lesions on structural brain networks at the contralesional hemisphere. Therefore, we aim to reconstruct and analyse structural connectomes after stroke both in the ipsi- and contralesional hemisphere. For this aim, we examine patients with upper limb motor deficits as a common and relevant clinical deficit in the chronic stage after stroke. We reconstruct the structural connectome based on diffusion-weighted imaging and probabilistic fibre tracking algorithm. We apply graph theoretical tools to connectome data to illustrate topological changes in comparison to healthy participants of comparable age. We hypothesize that at the ipsilesional hemisphere, topological measures of the connectome reflect a connectivity deficit as well as a disturbed configuration of structural networks. In addition, we aim to demonstrate changes in connectivity and network topology in the contralesional hemisphere that occur in the wake of structural diaschisis or adaptive and compensatory plasticity. Lastly, studying patients with motor deficits, we hypothesize that global and local graph parameters relate to clinical measures of motor impairment.

## Materials and methods

### Subjects

For this cross-sectional observational study, patients with first time, singular, supratentorial ischaemic stroke and upper limb motor deficits in the chronic stage (more than 6 months since stroke onset) were recruited from our university hospital. Exclusion criteria included contraindications to MRI, aphasia or hemianopia rendering patients unable to give written informed consent or participate in clinical evaluations as well as presence of neurological or psychiatric comorbidities. Motor deficits were quantified by means of whole handgrip force and the Fugl-Meyer assessment of the upper extremity (UEFM), which quantifies active movement range and synergies of upper extremity muscles on an ordinal scale. For grip strength testing, grip force of both hands (affected and unaffected) was measured three consecutive times using a hand dynamometer (Strength JAMAR hand evaluation kit, Elite healthcare, UK) and the average of three measurements was taken for further analysis. Clinical evaluation and testing of motor deficits were performed prior to MRI, all participants were scanned by MRI on the same day as clinical testing. Demographic data (gender, age and days since stroke onset) were recorded. In addition to patients, healthy participants of comparable age and gender were recruited. The study was approved by the local ethics committee. All participants gave written informed consent according to the Declaration of Helsinki.

### Imaging

Diffusion-weighted and high-resolution T_1_-weighted anatomical images were acquired using a 3 T Siemens Skyra MRI scanner (Siemens, Erlangen, Germany). For the former, 75 axial slices were obtained covering the whole brain with gradients (*b *=* *1500 s/mm^2^) applied along 64 non-collinear directions with the following sequence parameters: repetition time = 10 000 ms, echo time = 82 ms, field of view = 256 × 204, slice thickness = 2 mm, in-plane resolution = 2 × 2 mm. For the latter, a 3D magnetization-prepared, rapid acquisition gradient-echo sequence was used with the following parameters: repetition time = 2500 ms, echo time = 2.12 ms, field of view = 256 × 208 mm, 256 axial slices, slice thickness = 0.94 mm, and in-plane resolution = 0.83 × 0.83 mm.

### Reconstruction of the structural brain connectome

Undirected, weighted networks were constructed based on high-resolution structural imaging, diffusion tensor imaging and probabilistic tractography to approximate white matter fibre tracts as described previously (Schlemm *et al.*, 2017). In summary, diffusion-weighted images were analysed using the FSL software package 5.1 (http://www.fmrib.ox.ac.uk/fsl). All datasets were corrected for eddy currents and head motion. Structural T1-weighted anatomical images were processed using the FreeSurfer software package 5.3.0 with standard procedures and parameters resulting in a cortical parcellation of 34 cortical and 6 subcortical regions per hemisphere (Desikan *et al.*, 2006; Behrens *et al.*, 2007). Additionally, one brain stem region was defined manually in Montreal Neurological Institute space at the level of the pontomedullary junction comprising the area of descending motor tracts. For cortical regions, masks from automated parcellation by Freesurfer were refined to delineate the grey–white matter boundary underlying the cortical areas to increase anatomical accuracy for diffusion tensor imaging fibre tracking. Therefore, surface maps of the boundary between grey and white matter were generated by FreeSurfer and applied to constrain parcellation masks to a ribbon directly underlying the cortical grey matter. All resulting masks were visually checked for plausibility and accuracy. In total, 82 masks (41 per hemisphere) were created as listed in Supplementary Table 1. Stroke lesions were manually delineated on T1-weighted anatomical MR images and visually checked for plausibility and accuracy after registration to individual maps of fractional anisotropy. Stroke lesion masks were applied to exclude affected brain regions from probabilistic fibre tracking. In addition, pathways were disregarded if they entered a voxel with fractional anisotropy < 0.15. This value was chosen based on previous studies and experience (Cheng *et al.*, 2014; Finger *et al.*, 2016) showing this threshold consistently preventing erroneous tracking between neighbouring gyri.

Processing of diffusion data included application of a probabilistic diffusion model modified to allow estimation of multiple (*n* = 2) fibre directions using the program bedpostX. Probabilistic fibre tracking was carried out using the command probtrackx2 as implemented in FSL and described previously (Schlemm *et al.*, 2017). From each seed region of interest (ROI) voxel, 5000 streamlines were initiated through the probability distribution on principle fibre direction. Structural connectivity between two regions was measured by masking each seed ROI results by each of the remaining ROIs. Both whole-brain and intrahemispheric fibre tracking were performed to individually create connectivity matrices including and excluding interhemispheric (transcallosal) connections. For intrahemispheric network reconstruction, tracking streamlines were discarded when they intersected the midline as defined by a manually generated exclusion mask. Weighted connectivity matrices were computed by defining the strength of the connection from ROI *s* to ROI *t* to be given by the raw number of streamlines starting in *s* and running through *t*, divided by the sum of the volumes of *s* and *t*. Network reconstruction resulted in connectivity matrices of dimension 82 × 82 and 41 × 41, for whole-brain and intrahemispheric networks, respectively, which were symmetrized by averaging with their own transpose.

### Graph theoretical analysis of structural networks

Properties of the structural connectome were quantified on the level of global connection strength, global and local topological graph parameters as well as patterns of edgewise connectivity. For the latter two, the Brain Connectivity Toolbox (Version 2017-15-01) (Rubinov and Sporns, 2010) and the network-based statistics (NBS, v1.2) toolbox (Zalesky *et al.*, 2010) were used, respectively. In detail, we first calculated median connectivity strength (q_50_) of each network. Second, we characterized prominent topological aspects of network architectures using global graph parameters (GGP). Specifically, we chose global efficiency, clustering and modularity since these measures are robust and well suited to capture topological properties in large-scale networks. Whereas global efficiency can be viewed as a measure of network integration, global clustering and modularity reflect the element of segregation in large-scale brain networks (Bullmore and Sporns, 2009; Welton *et al.*, 2015). In weighted networks, the former two are strongly influenced by both connection strength and topology (Rubinov and Sporns, 2010). Therefore, we normalized global efficiency and clustering in relation to 1000 simulated random networks with equal distribution of edge weight, node strength and degree to increase comparability between individual connectomes as described previously (Rubinov and Sporns, 2011; Schlemm *et al.*, 2017). Furthermore, measures of network architecture are sensitive to the density at which networks are analysed (van Wijk *et al.*, 2010; Fornito *et al.*, 2013). Estimates of anatomical connection density depend on the resolution of the underlying cortical parcellation and fibre reconstruction method (Bassett and Bullmore, 2017), with values ranging from <30% in the binary case to 66% in a Macaque tract-tracing study (Markov *et al.*, 2013). The optimal choice of network density for the analysis of imaging-derived weighted structural connectomes therefore remains controversial (Civier *et al.*, 2019). As done previously (Seguin *et al.*, 2018; Buchanan *et al.*, 2019), weighted brain networks were therefore thresholded by multiplication by a sparsity mask corresponding to the 20%, 25%, (…), 75% and 80% strongest connections in the average network of healthy participants and analysed over this range of network densities. We computed GGP separately for all whole-brain and intrahemispheric networks (including and excluding interhemispheric transcallosal connections, respectively). Lastly, the local graph parameter node strength (Rubinov and Sporns, 2010) was calculated to quantify aspects of a network’s architecture in the vicinity of a specific brain region (node) to localize changes in network structure at specific regions in the brain.

Whereas the aforementioned graph parameters characterize network topology at the level of interconnected nodes, changes in the structural connectome can also be described focusing on configuration of edges, i.e. interconnecting white matter tracts. For this purpose, we applied the NBS toolbox as described previously (Zalesky *et al.*, 2010). In summary, this approach identifies and visualizes subnetworks of edges with significant differences in connection strength between patients and healthy participants. Similar to traditional cluster-based thresholding, NBS applies a two-step method that exploits the extent to which edges with a large difference in connectivity are interconnected, therefore addressing the issue of family-wise error rate of the set of identified edges.

A fixed proportional thresholding of 50% was applied to structural connectomes for the analysis of local network parameters. This value was chosen so as to be consistent with both recent interval estimates of the mammalian structural connectome (Bassett and Bullmore, 2017) and the peak intrahemispheric efficiency we observed in our data. A sensitivity analysis for group differences in local network architecture with respect to network density was performed.

### Computational lesion model

**Figure 1:**
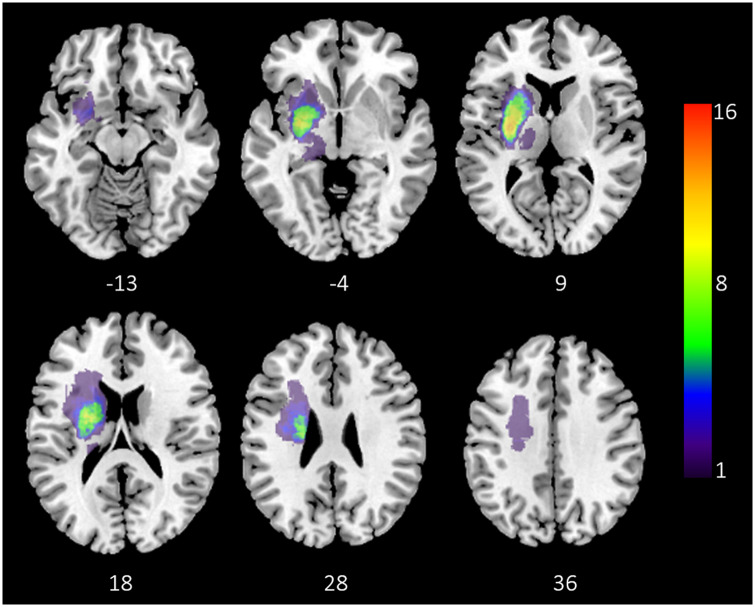
**Lesion overlay.** Overlay plot illustrating the stroke lesion distribution of all patients (*n* = 17), slice numbers represent *z* coordinates corresponding to the standard MNI152 brain template. Number of affected patients is represented by colour. For illustrative purposes, all lesions were flipped to the same hemisphere.

In addition to empirically observed alterations of the structural connectome, we were interested in basic computational lesion models that could reproduce and help to interpret observed changes in network topology of chronic stroke patients. Based on the topological assumption of small-world characteristics as one key feature of the human connectome (Bassett and Bullmore, 2006), we simulated the influence of stroke lesions on overall network topology using the Watts–Strogatz model which generates networks exhibiting small-worldness characteristics similar to a prominent topological feature observed structural brain networks (Bassett and Bullmore, 2006). We used the Network Generation and Analysis Toolbox implemented in Matlab (Sizemore *et al.*, 2017) to simulate *n* = 38 realizations of the Watts–Strogatz model (matching the total number of patients and control subjects) with 41 nodes (matching the number of regions of interest in each hemisphere). The following configuration parameters were used: *P* = 0.25 (probability of rewiring an edge of the ring lattice into a random short cut connection) and *k* = 10 (desired mean degree for each node). The latter parameter was chosen matching the average degree of the empirical brain networks at a density of *κ* = 0.27. Following generation of network models, we simulated the effect of virtual lesions in a paradigm that preferentially affects long-range connections. This paradigm was chosen with respect to the stroke lesion distribution in our group of patients that almost exclusively affected subcortical regions (see Fig. 1). The resulting network disruption was then quantified by graph theoretical measures analogous to the empirical data. As before, global efficiency and clustering were normalized with respect to the average value in 1000 null models obtained from degree-, strength- and weight-preserving a randomization.

### Statistical analysis

We quantified network alterations in ipsi- and contralesional hemispheres in comparison to connectomes from age- and gender-matched healthy participants in terms of global and local network properties. Median connectivity strength was analysed using a mixed-effects linear model including (i) the fixed effect ‘side’ with levels left and right; (ii) the fixed effect ‘lesion status’ with levels ipsilesional, contralesional and healthy participants; (iii) the random factor ‘subject’. The model was first fitted with interaction terms; in the absence of a statistically significant interaction it was refitted without interactions, followed by *post hoc* analyses of main effects. Effect sizes in omnibus tests were quantified as ratios of likelihoods corresponding to models with and without the factor of interest. Residuals were tested against normality using the Shapiro–Wilk test (Shapiro and Francia, 1972). For GGP, effects of lesion status on efficiency, clustering and modularity were analysed over the entire range of thresholds using the multi-threshold permutation correction approach (Drakesmith *et al.*, 2015). This technique accounts for both the magnitude of the effect at a particular network density and its persistence across network densities and thus mitigates the multiple comparison problem associated with analysing the networks at different sparsity levels. It is thus threshold-agnostic and eliminates the necessity to pre-specify an arbitrary network density. The method was first applied to whole-brain GGP as well as left and right intrahemispheric GGP, respectively. To isolate the effect of lesion status, data were then pooled and an multi-threshold permutation correction analysis was performed for the residuals obtained from subtracting from each data point the corresponding hemisphere- and density-specific group average. For group comparison of local network properties, a mass univariate analysis was chosen. Mixed-effects linear models as before were fitted for local graph parameters of individual ROIs and assessed for statistical significance of lesion status. After correction for multiple testing using the Bonferroni approach, brain regions showing altered local network characteristics were identified and subjected to *post hoc* tests. We investigated significant differences in white matter (edgewise) connectivity using NBS (Zalesky *et al.*, 2010). Therefore, a range of *t*-values ranging from 1 to 5 was employed in the first step to detect critical differences in edgewise connectivity. A conservative *t*-value of 3.1, corresponding to an edgewise significance level of *P* ≈ 0.0025, which maximized the statistical significance of detected network disruptions was chosen to localize the relevant subnetwork (a more detailed description of this approach can be found in the Supplementary material). Imaging-derived measures of connectivity strength as well as global and local network topology were assessed for association with motor performance scores (grip strength and UEFM) using linear regression analysis. All statistical analyses were conducted in the R computing environment (R Core Team, 2008).

### Data availability

Data from this study is available for researches upon reasonable request.

## Results

### Subjects

Table 1 displays an overview of the subjects included in this study. A total of 17 patients were recruited (13 females). Mean age was 64.5 ± 8.4 years, 8 (47%) patients had lesions in the left hemisphere. In addition, 21 healthy participants with comparable distributions of age and gender (10 females, age 64.9 ± 10.3 years) were recruited. More detailed individual values for demographic, clinical and graph theoretical data can be found in Supplementary Table 2. There was no statistically significant association between age and disease status [*F*(2,35) = 0.009, *P* = 0.999] or gender and disease status (χ^2^ = 3.287, simulated *P* = 0.219). Lesion distribution is illustrated in Fig. 1 with lesion volume ranging between 0.1 ml and 3.9 ml (median 0.8 ml, interquartile range (IQR) 0.3–1.7 ml). All stroke lesions were located in brain areas supplied by the middle cerebral artery involving the basal ganglia and subcortical white matter. In a minority of patients, cortical insular regions were affected. Primary sensory and motor cortices were unaffected by stroke lesions in all patients. Analysis of motor scores indicated a deficit in grip strength at the affected compared to the unaffected hand (Cohen’s *d* = 1.06, *P* = 0.004). There was no significant association between lesion volume and grip strength of the affected hand (*r* = 0.44; *P* = 0.075) as well as UEFM (*r* = 0.12; *P* = 0.639).

**Table 1:** Demographic and clinical details

	Patients (*n* = 17)	Controls (*n* = 21)	*P*-value
Female (%)	13 (76.5)	10 (47.6)	0.140
Mean age [years] (±SD)	64.5 (±8.4)	64.9 (±10.3)	0.919
Lesion on the left (%)	8 (47.1)		
Days since stroke (median, IQR)	377 (363–610)		
Delta grip strength [kg] (median, IQR)	5.66 (4.00–13.00)		
UEFM (median, IQR)	66 (60–66)		

Baseline characteristics of stroke patients and healthy controls included in the study. *P*-values are based on group comparison by unpaired *t*-tests.

### Brain network measures and graph theoretical analysis

We investigated three aspects of structural brain networks in association with clinical parameters to capture the integrity and topological changes of the structural connectome during the chronic stage after stroke. Therefore, we applied measures from graph theory that quantify the degree of overall connectivity, network organization (topology) and localization of pronounced network changes in specific anatomical brain areas.

**Figure 2:**
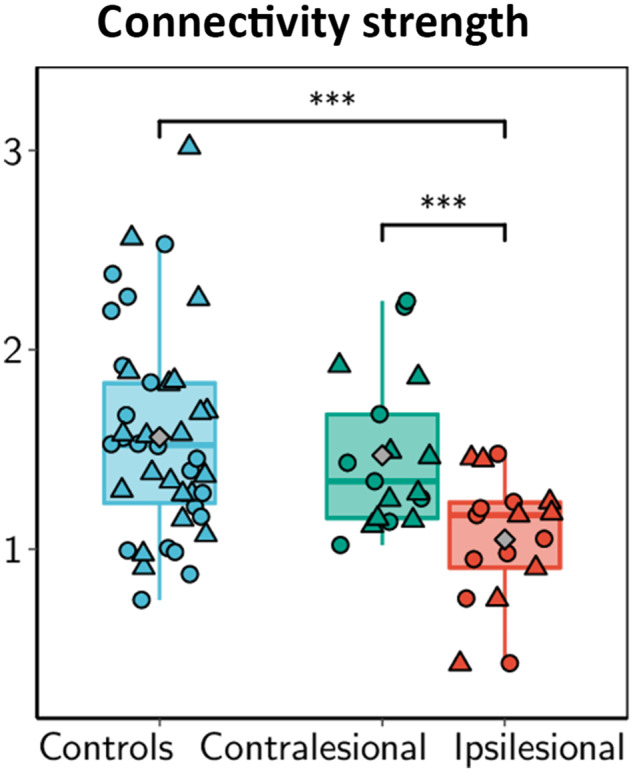
**Global intrahemispheric connectivity strength q50 stratified by lesion status.** Boxplots show median values. Diamonds (grey colour) mark the group mean in healthy controls (blue), contralesional (green) and ipsilesional (red) hemispheres. Circles and triangles indicate left and right hemispheres, respectively. Individual contrasts with a statistical significance exceeding *α* = 0.05 are indicated by brackets. *** *P* < 0.0005.

First, overall connectivity of structural brain networks was quantified by means of median connectivity strength and compared to healthy participants as are shown in Fig. 2. Median connectivity strength q_50_ was different between hemispheres of healthy participants, contralesional hemispheres and ipsilesional hemispheres (Λ_2_ = 16.933, *P* < 0.001). *Post hoc* tests detailed in Table 2 indicated overall reduced connection strength in ipsilesional hemispheres compared to both contralesional hemispheres and hemispheres of healthy participants. A trend of lower connection strength was also detected in contralesional hemispheres compared to healthy participants although this did not reach statistical significance. Inclusion of age and sex as cofactors did not essentially alter the results of this analysis (Supplementary Table 3). There was no evidence for a significant difference in global connectivity between left and right hemispheres [q_50_: *F*(1,37) = 0.112, *P* = 0.739; λ: *F*(1,37) = 0.865, *P* = 0.358] or a significant interaction between lesion status and side of the lesion (q_50_: Λ_2_ = 0.779, *P* = 0.677; λ: Λ_2_ = 2.702, *P* = 0.259). No statistically significant associations between global connectivity and grip strength of the affected hand or the UEFM were observed (see Supplementary Table 4 for details).

**Table 2:** Median connectivity strength (q50) in intrahemispheric connectomes

	Group and lesion status			Test statistic
	Controls	Ipsilesional	Contralesional	
Any hemisphere	1.56 ± 0.08^a^	1.05 ± 0.08	1.47 ± 0.09^a^	*F*(2,36.65) = 9.29; *P* = 0.001
Left hemisphere	1.60 ± 0.11^a^	1.07 ± 0.13	1.41 ± 0.10^a^	*F*(2,35) = 4.02; *P* = 0.027
Right hemisphere	1.53 ± 0.11^a^	1.03 ± 0.10	1.54 ± 0.176^a^	*F*(2,35) = 4.05; *P* = 0.026

Test statistics in the last column represent the effect of lesion status (control, ipsilesional and contralesional). Identical superscripts within a row indicate the absence of a statistically significant difference in *post hoc* mean-separation testing. See also Fig. 2 for illustration.

**Figure 3:**
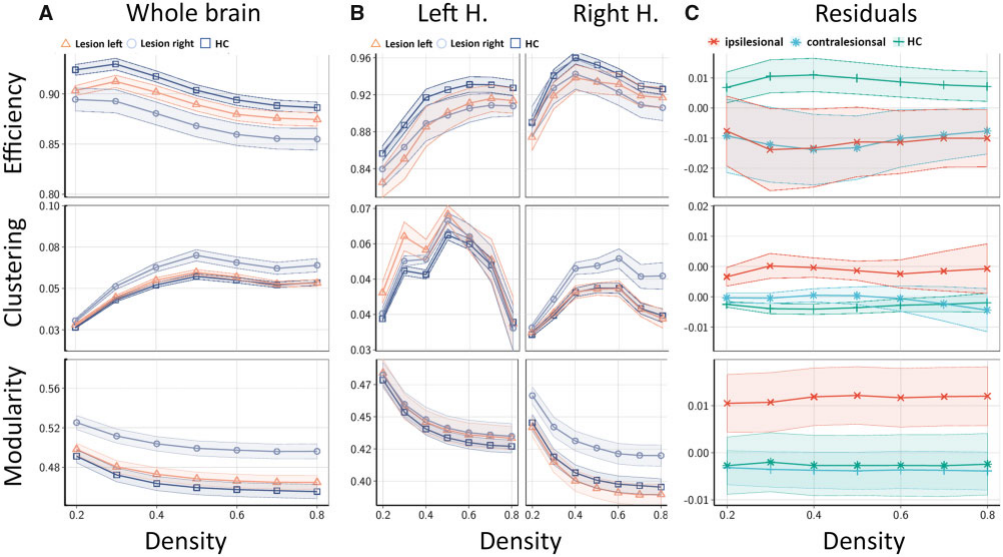
**GGP as functions of network density.** Global efficiency, clustering and modularity of whole brain (**A**) as well as intrahemispheric brain networks (**B**) for patients with lesions in the left (triangle marker) and right (circle marker) hemispheres, as well as healthy controls (box marker). Ribbons indicate standard errors of the mean. Efficiency and clustering have been normalized with respect to average values in n=1000 strength-, degree- and weight-preserving random null models. (**C**) Residuals of hemispheric GGP after subtraction of the average values in left and right hemispheres for healthy controls (+), hemispheres affected by stroke (×), as well as contralesional hemispheres (∗). For ease of visualization, the curves in the second row in panels (**A**) and (**B**) represent the difference between the normalized clustering coefficients and one, multiplied by the square of the network density, i.e. $\left(C-1\right)\kappa^{2}$. HC = healthy controls.

Second, we investigated topological changes of the connectome after stroke that can be considered as the layout and organization of structural brain networks that are not captured by measures of connection strength alone. These topological features are described by measures of graph theory that indicate the degree of integration and segregation in structural networks (Bullmore and Sporns, 2009; Welton *et al.*, 2015). As described in the Materials and methods section, we chose global efficiency, clustering and modularity to capture these aspects; results are illustrated in Fig. 3. In the analysis of whole-brain networks (Fig. 3A), normalized global efficiency was reduced in connectomes of patients with both left- and right-sided lesions compared to brain networks of healthy participants. Normalized global clustering and modularity were increased in stroke patients compared to normal participants with a more pronounced effect in right-sided lesions. In the separate analysis of intrahemispheric networks excluding interhemispheric connections (Fig. 3B), we observed similar results. Normalized global efficiency was reduced equally in both ipsi- and contralesional hemispheres, whereas the increase in clustering, while present both ipsi- and contralesionally, was more pronounced in directly affected ipsilesional hemispheres. For both measures, the effects were independent from hemisphere side (left and right). Intrahemispheric modularity was increased ipsilesionally in patients with right-sided stroke and unchanged in contralesional hemispheres. Details of the statistical analysis are reported in Supplementary Table 5.

**Figure 4:**
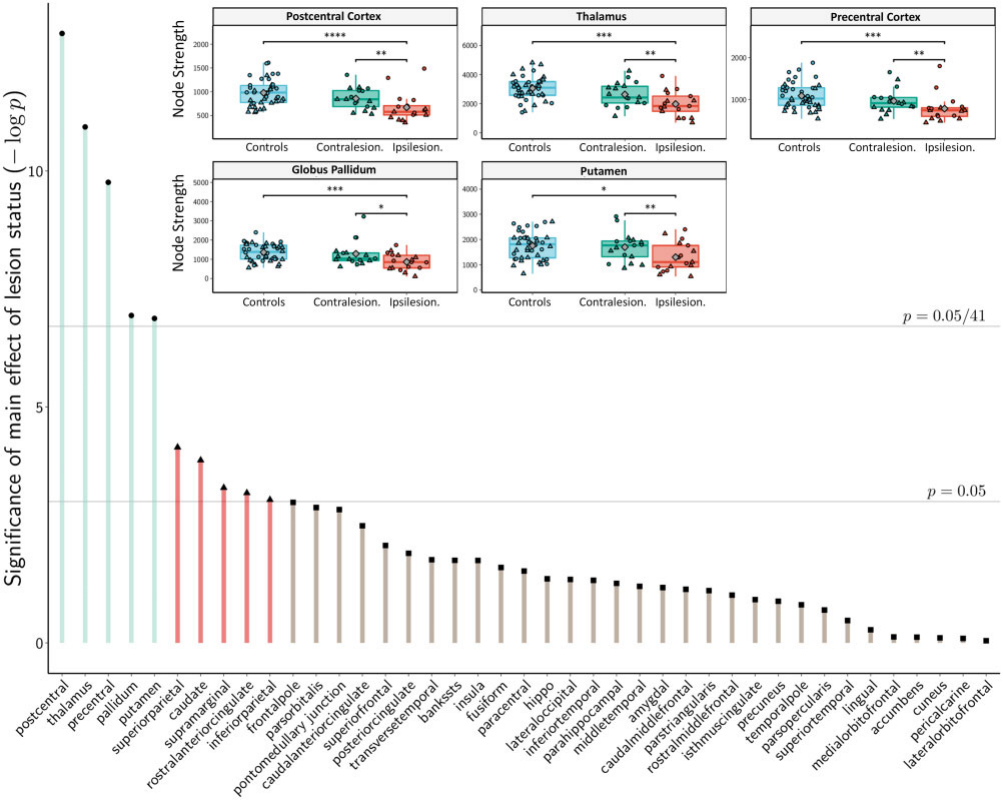
**Connectome disruption quantified by local node strength.** Height of bars indicate strength of statistical evidence from linear mixed-effects modelling for main effect of lesion status (ipsilesional, contralesional and healthy control) on local node strength of each predefined region (negative logarithm of the obtained *P*-value). Horizontal lines correspond to significant effect in mass univariate modelling (*P* = 0.05 and *P* = 0.05/41 as the criticality threshold after Bonferroni correction). *Inset*: Node strength of five brain regions exhibiting a significant effect of lesions status after Bonferroni correction. Diamonds mark the group means. Individual circles and triangles indicate left and right hemispheres, respectively. Individual contrasts with a statistical significance are indicated by brackets. * *P* < 0.05; ** *P* < 0.005; *** *P* < 0.0005.

**Figure 5:**
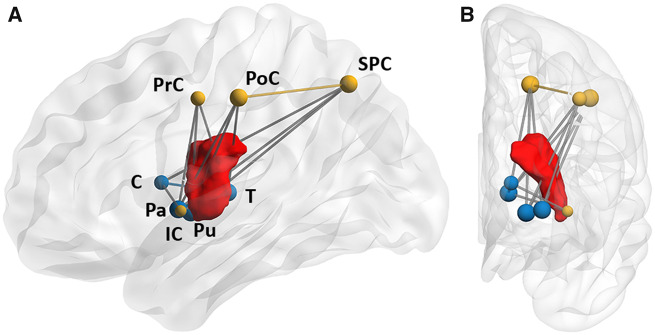
**NBS analysis.** Lateral (**A**) and frontal (**B**) projections on a standard brain template. Displayed is the ipsilesional subnetwork consisting of edges with a connectivity deficit compared to healthy controls, quantified by an edgewise *t*-statistic exceeding a threshold value of *t* = 3.1. Blue and yellow spheres indicate subcortical and cortical regions, respectively. To illustrate 3D spatial co-localizations with stroke lesion, core lesion distribution (red object) from the majority of patients is shown (11 of 17 patients, see also Fig. 1 for a 2D representation of stroke lesions). C = caudate nucleus; IC = insular cortex; Pa = pallidum; PoC = postcentral cortex; PrC = precentral cortex; Pu = putamen; SPC = superior parietal cortex; T = thalamus.

Third, we aimed to localize the reported changes of network connectivity and topology to specific anatomical brain regions using local graph theoretical parameters and NBS mapping. As shown in Fig. 4, we identified five brain regions with significant changes in local node strength compared to healthy participants based on an analysis by mass univariate linear mixed-effects modelling. Local node strength was significantly reduced in ipsilesional hemispheres compared to contralesional hemispheres and healthy participants and most prominently reduced in the post- and precentral cortices as well as the thalamus, pallidum and putamen (after Bonferroni correction for multiple comparisons). There were no significant interactions of lesion status with hemisphere side. These results were stable across a range of network densities imposed by proportional thresholding (Supplementary Fig. 1). As an alternative and complementary approach, we applied NBS to identify connections and subnetworks that are significantly altered in our group of patients. Results are shown in Fig. 5. The resulting subnetwork comprises edges with significantly lower connectivity in the ipsilesional hemisphere compared to healthy participants. These include cortico-subcortical white matter tracts between the superior temporal gyrus, pre- and postcentral gyrus, insular cortex, caudate nucleus thalamus, putamen, and pallidum. Results over a wider range of initial *t*-thresholds are shown in Supplementary Figs 2 and 3.

### Simulated network lesion model

**Figure 6:**
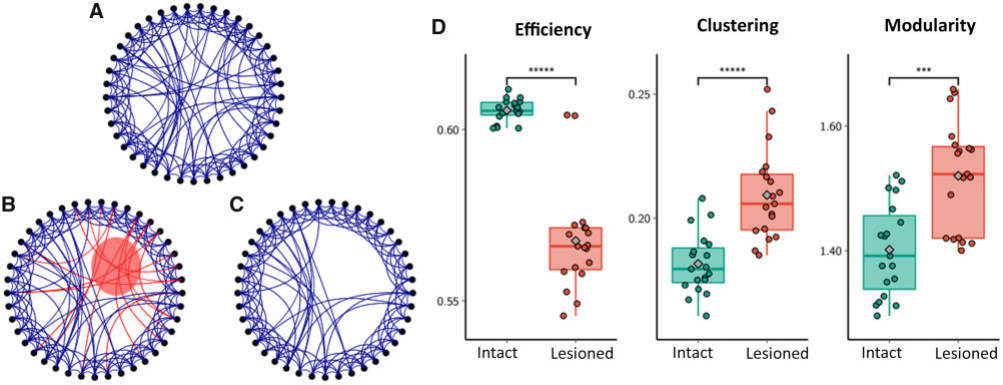
**Effect of lesioning long-range connections in the Watts–Strogatz model.** (**A**) Simulation of a network model using a weighted ring lattice with 41 nodes and mean degree 10. (**B**) Subsequently, a proportion of long-range connections is removed, modelling the effect of a focal, e.g. ischaemic, lesion (red circle). (**C**) Resulting ‘lesioned’ network model; (**D**) GGP efficiency, clustering coefficient and modularity of 38 realizations (21 intact, 17 lesioned) of Watts–Strogatz networks. The former two measures were normalized with respect to *n* = 1000 strength-, degree- and weight-preserving random null models. Diamonds mark the mean and brackets a significant statistical group difference. *** *P* < 0.0005, ***** *P* < 0.000005.

Figure 6 illustrates the effect of simulating focal lesions in a computational network model with prominent small-world properties. Removing a proportion (q = 0.5) of long-range connections from the network architecture resulted in an overall and significant decrease of normalized global efficiency as well as an increase of normalized global clustering and modularity.

## Discussion

In our study, comprehensive analysis of structural brain networks in chronic stroke patients yielded two major results: Firstly, significantly reduced structural connectivity was apparent in the ipsilesional hemisphere. Secondly, graph theoretical analysis revealed a disturbed topology of structural brain networks in both ipsi- and contralesional hemispheres characterized by lower integration and higher segregation. These topological alterations were apparent from overall decreased global efficiency (as a measure of integration) and increased global clustering and modularity (as measures of network segregation) in structural connectomes of stroke patients compared to healthy participants. We did not detect associations with clinical parameters of motor impairment with global or local graph theoretical measures.

Rapid and persisting disruption of structural brain connectivity is the key characteristic of ischaemic stroke underlying clinical deficits. In our group of patients in the chronic stage after stroke, these effects were apparent in marked overall reduction of white matter connectivity at the ipsilesional hemisphere. In terms of a more localized, network-based analysis, these changes were most prominently shown affecting nodes (i.e. brain regions) involved in the control and execution of motor functions. Local node strength, a measure of nodal connectivity, was significantly reduced in the pre- and postcentral cortex, superior and inferior parietal cortical areas as well as the basal ganglia (Fig. 4). Whereas altered connectivity of subcortical regions directly affected by the stroke lesions are self-evident, altered nodal graph measures in primarily unaffected brain regions (such as the pre- and postcentral cortex) can be explained by structural disconnections of cortical areas after stroke. In accordance with this mechanism, separate analysis of edgewise connection strength by NBS detected reduced structural integrity involving connections in a cortico-subcortical subnetwork surrounding the focus of stroke lesions in our patients (Fig. 5).

Beyond the extent of reduced global connectivity in the ipsi- and contralesional hemisphere, we characterized changes of large-scale network topology at the chronic stage after stroke. Specifically, we were interested in structural network measures that promote integration and segregation of brain function that are known to be altered in various neurological diseases that are not captured directly by measures of connectivity strength (Bassett and Bullmore, 2009). For this purpose, we have chosen the parameters of global efficiency, clustering and modularity as established measures of brain network topology in various neurological diseases and to facilitate comparability of our results with previous studies (Aerts *et al.*, 2016). To account for the influence of connection strength on these measures, we aimed to establish the significance of network measures by comparison with statistics calculated on null hypothesis networks (Albert and Barabási, 2002). Our results show that in the chronic stage after stroke, structural brain networks were characterized by lower global efficiency and increased global clustering compared to healthy, age-matched participants. These changes were robust and consistent over various network densities and affected the ipsilesional as well as contralesional hemisphere independent of lesion side. Changes in normalized modularity were characterized by higher values at the ipsilesional hemispheres compared to healthy participants. Taken together, these results point toward an altered topology of the structural connectome with a lower potential to integrate functional activity between various, distant brain areas. On the contrary, structural networks of stroke patients were characterized by increases of localized, clustered and small-scale subnetworks. Regarding our group of patients with mainly subcortical stroke lesions, we hypothesize that these changes in network topology are best explained by ischaemic injury and consecutive disintegration of long white matter fibre tracts interconnecting distant cortical and subcortical areas. To further investigate this hypothesis, we applied an established, basic mathematical network model (Watts–Strogatz) simulating characteristic aspects of brain network topologies, specifically small-world properties (Fig. 6). We found that a paradigm with selective loss of long distant connections led to changes in global efficiency, clustering and modularity analogous to observations in the ipsilesional hemisphere of stroke patients. Application of this simplified model illustrates how single, strategically localized lesions can lead to the ‘break down’ of efficient network architecture observed in our stroke patients. Although the network model chosen in our study reflects on basic (yet fundamental) properties of neuronal networks, it remains oversimplified and does not account for the far more complex configuration of the human connectome. Therefore, comprehensive, large-scale models and simulations, optimally in native brain space are needed that would exceed the purpose of this study.

Contrary to changes of post-stroke global functional brain connectivity, reports on alterations in large scale, whole-brain structural connectomes after stroke are scarce. In a previous topological network analysis, ‘communicability’ as a localized, nodal measure of efficient information transfer was significantly reduced in structural connectomes of nine patients in the early chronic stage (4–5 weeks) after stroke (Crofts *et al.*, 2011). Compared to age-matched healthy controls, changes were most prominent ipsilesional and in regions surrounding the stroke lesion, comparable to our observations in the ipsilesional hemisphere by nodal and edgewise network analysis. In addition, classification algorithms separated patients from healthy participants based on lower communicability values in homologous brain regions at the contralesional hemispheres. In contrast to findings from our study, global graph measures such as efficiency and clustering were not significantly divergent between patients and healthy controls. This is potentially due to the significantly larger interval after stroke in our group of patients, as structural white matter disintegration is known to evolve over time and become more prominent and more easily detected by structural imaging modalities such as diffusion tensor imaging. Focusing on changes of the contralesional hemisphere, a recent experimental animal study applied ultra-high-resolution diffusion MRI to reconstruct structural brain networks 70 days after experimental stroke in post-mortem rat brains (Sinke *et al.*, 2018). Graph theoretical measures were calculated at contralesional hemisphere using a minimum spanning tree algorithm to detect relevant changes of network topology, specifically the ‘back bone’ of fundamental structural connections. As a result, several topological features of contralesional brain networks were associated with sensory-motor deficits. Specifically, high network eccentricity (longer path lengths between nodes in a spanning tree) was associated with worse performance in tests of sensory and motor behaviour. Longer path lengths between nodes are also the underlying base for a decrease of global efficiency. Thus, these results from experimental stroke models would be in line with our observations of decreased global efficiency in chronic stroke patients. Taken together, these results point towards alterations of structural network topology at the contralesional hemisphere that is not directly affected by a stroke. Our results also indicate a globally reduced structural connectivity in the contralesional hemisphere, which, however, did not reach statistical significance in *post hoc* testing. At the level of network topology, significant changes were apparent with structural connectome topology being shifted to a less optimal configuration with lowered ability to integrate various brain regions segregated into a higher number of clusters with lesser degree of long-distance interconnections. In terms of a conceptual framework of complex networks, these changes signify an alteration from the proposedly optimal ‘small world’ configuration towards a more random topology involving both hemispheres after stroke (Aerts *et al.*, 2016). Analogous to the underlying damage to white matter tracts at the ipsilesional hemisphere, we propose that degeneration of long range, white matter tracts are the primary pathophysiological mechanism of disturbed network topology in contralesional hemispheres of stroke patients. Distant effects of stroke lesions at the contralesional hemisphere were previously reported in terms of white and grey matter disintegration in contralesional hemispheres after stroke (Koliatsos *et al.*, 2004; Avanzino *et al.*, 2007; Schaechter *et al.*, 2009; Cheng *et al.*, 2019). Although the primary mechanism of these effects at the distance of stroke lesions are still to be elucidated, they are most likely induced by transneuronal mechanisms via the degeneration of interneurons at the cerebral cortex and transcallosal fibres subsequently resulting in apoptosis of contralesional white matter tracts. However, other factors, such as diffusely distributed cortical hypometabolism found in previous studies of diaschisis after subcortical stroke at the contralesional hemisphere are potential contributors (Kushner *et al.*, 1984; Pappata *et al.*, 1990). Taken together, our observations demonstrate the impact of focal lesions on contralesional connectomes as a form of ‘Connectomal Diaschisis’ in analogy to the well-described effects of ipsilateral lesions to distant brain structure and function after stroke (Carrera and Tononi, 2014).

In our study, we focused on patients with upper limb deficits that have a major impact on daily activities and occur frequently in stroke populations. In addition, recovery of upper extremity function after stroke has been extensively studied by structural MRI studies highlighting brain structures crucial for clinical outcomes such as the pyramidal tract or connected primary and secondary motor areas. Disturbances of structural organization in the chronic phase after stroke were not associated with severity of clinical motor impairment as measured by grip strength and the UEFM. This is in contrast to several neurological diseases where alterations of the structural brain network topology were related to the degree of clinical deficits (Aerts *et al.*, 2016). Lack of clinical implications in our study might stem from the relatively small patient number or long time interval after stroke, were adaptive mechanisms in brain function have led to rehabilitation of initial motor impairment ‘despite’ extensive damages to structural brain networks (Aerts *et al.*, 2016).

Several limitations of our study have to be mentioned. Considering the cross-sectional design of our study and lack of longitudinal data, we cannot conclude that the observed alterations in structural network integrity and topology are solely caused by the localized stroke lesion. Although the observed changes are pathophysiologically plausible and reproducible in a basic network simulation model, it is conceivable that they are influenced by other premorbid risk factors for cerebrovascular diseases such as hypertension or diabetes commonly present in stroke patients. Since we did not systematically record these potentially confounding factors, further studies applying a multivariate analysis and longitudinal design are needed to elucidate the significance of potential premorbid vulnerabilities of brain networks in stroke patients. In terms of adaptive mechanisms, changes of network measures such as increased communicability have been found in the two previous studies mentioned above and discussed to indicate structural plasticity after stroke (Crofts *et al.*, 2011; Sinke *et al.*, 2018). In our study, we did not observe increases in structural connectivity or topological changes that would indicate adaptive improvements in network function, most likely due to the cross-sectional design of our study. Given the sample size of 17 patients, it was necessary to restrict attention to a small number of well-established network measures in order to minimize the risk of overfitting. Results should therefore be confirmed and extended to more subtle network properties in larger cohorts of stroke patients. Further studies including data from functional brain imaging, are needed to clarify the extent and role of compensatory structural network changes after stroke. Lastly, due to the pre-specified phenomenological selection of stroke patients in our study, we are unable to draw conclusions as to the generalizability of our results to stroke patients demonstrating different lesion patterns (i.e. mainly involving cortical areas).

In summary, we demonstrate that stroke lesions affect not only structural integrity but also topology of the human structural connectome. Our findings suggest that alterations in white matter structure, primarily of long fibre tracts connecting distant brain areas, influence network measures of efficiency of communication in both hemispheres following stroke. Further studies are needed to investigate the functional relevance of disturbed optimal balance between regional segregation and interregional integration in structural connectomes after stroke.

## Funding

This research was supported from the German Research Foundation (DFG) SFB-936 Multi-site Communication in the Brain (projects A1, C1 and C2).

## Competing interests

The authors report no competing interests.

## Supplementary Material

fcz020_Supplementary_Data

## Abbreviations

GGP =: global graph parameters
MNI =: Montreal Neurological Institute
MRI =: magnetic resonance imaging
NBS =: network-based statistics
ROI =: region of interest
UEFM =: Fugl-Meyer assessment of the upper extremity
