# Supplementary material for: Altered topology of large-scale structural brain networks in chronic stroke
Source: Brain Commun. 2019 Oct 4;1(1):fcz020. doi: 10.1093/braincomms/fcz020 (PMC7425306; doi:10.1093/braincomms/fcz020)
Supplement: fcz020_Supplementary_Data [file fcz020_supplementary_data.zip › Manuscript_revision_1.pdf]

OXFORD UNIVERSITY PRESS | Brain Communications

Altered topology of large-scale structural brain networks in chronic stroke

Journal:	*Brain Communications*
Manuscript ID	BRAINCOM-2019-032.R1
Manuscript Type:	Original Article
Date Submitted by the Author:	04-Aug-2019
Complete List of Authors:	Cheng, Bastian; University Medical Center Hamburg Eppendorf, Department of Neurology<br>Schlemm, Eckhard; University Medical Center Hamburg Eppendorf, Department of Neurology<br>Schulz, Robert; University Medical Center Hamburg-Eppendorf, Department of Neurology<br>Boenstrup, Marlene; University Medical Center Hamburg Eppendorf, Department of Neurology<br>Messé, Arnaud; University Medical Center Hamburg-Eppendorf, Department of Computational Neuroscience<br>Hilgetag, Claus; University Medical Center Hamburg-Eppendorf, Department of Computational Neuroscience<br>Gerloff, Christian; University Medical Center Hamburg, Cortical Physiology Research Group, Department of Neurology<br>Thomalla, Götz; University Medical Center Hamburg-Eppendorf, Neurology
Keywords:	

SCHOLARONE™
Manuscripts

Altered topology of large-scale structural brain networks in chronic stroke

Bastian Cheng*[1], Eckhard Schlemm*[1], Robert Schulz[1], Marlene Boenstrup[1,2], Arnaud Messé[3], Claus Hilgetag3, Christian Gerloff[1], Götz Thomalla[1]

[1] Department of Neurology, University Medical Center Hamburg-Eppendorf, Hamburg, Germany

[2] Human Cortical Physiology and Neurorehabilitation Section, National Institute of Neurological Disorders and Stroke, National Institutes of Health, Bethesda, MD, USA

[3] Institute of Computational Neuroscience, University Medical Center Hamburg-Eppendorf, Hamburg, Germany

* Both authors contributed equally

Correspondence:

Bastian Cheng, MD
University Medical Center Hamburg Eppendorf
Martinistraße 52
D20246 Hamburg
fon: 0049-40-7410-51082
fax: 0049-40-7410-57391
E-Mail: b.cheng@uke.de

Short title: Structural brain networks in chronic stroke

Keywords: Cerebral ischemia; Plasticity; Imaging methodology; Axon degeneration

For Review Only

Abbreviations

MRI:	Magnetic Resonance Imaging
EEG:	Electroencephalography
UEFM:	Fugl-Meyer assessment of the upper extremity
MNI:	Montreal Neurological Institute
DTI:	Diffusion Tensor Imaging
FA:	Fractional Anisotropy
ROI:	Region of Interest
NBS:	Network-based Statistics
GGP:	Global Graph Parameters
MTPC:	Multi-Threshold Permutation Correction
MCP:	Middle Cerebral Artery

For Review Only

Abstract

Beyond disruption of neuronal pathways, focal stroke lesions induce structural disintegration of distant, yet connected brain regions via retrograde neuronal degeneration. Stroke lesions alter functional brain connectivity and topology in large-scale brain networks. These changes are associated with the degree of clinical impairment and recovery. In contrast, changes of large-scale, structural brain networks after stroke are less well reported. We therefore aimed to analyse the impact of focal lesions on the structural connectome after stroke based on data from diffusion-weighted imaging and probabilistic fiber tracking. In total, 17 patients (mean age 64.5 ± 8.4 years) with upper limb motor deficits in the chronic stage after and 21 healthy participants (mean age 64.9 ± 10.3 years) were included. Clinical deficits were evaluated by grip strength and the upper extremity Fugl-Meyer assessment. We calculated global and local graph theoretical measures to characterize topological changes in the structural connectome. Results from our analysis demonstrated significant alterations of network topology in both ipsi- and contralesional, primarily unaffected, hemispheres after stroke. Global efficiency was significantly lower in stroke connectomes as an indicator of overall reduced capacity for information transfer between distant brain areas. Furthermore, topology of structural connectomes was shifted toward a higher degree of segregation as indicated by significantly higher values of global clustering and modularity. On a level of local network parameters, these effects were most pronounced in a subnetwork of cortico-subcortical brain regions involved in motor control. Structural changes were not significantly associated with clinical measures. We propose that the observed network changes in our patients are best explained by the disruption of inter- and intrahemispheric, long white matter fiber tracts connecting distant brain regions. Our results add novel insights on topological changes of structural large-scale brain networks in the ipsi- and contralesional hemisphere after stroke.

For Review Only

Introduction

Ischemic stroke is one of the major causes of adult disability worldwide, often leading to deficits of motor, cognitive or language functions and thus impairments in daily life. In addition to localized disruption of neuronal pathways, stroke lesions induce remote effects on brain function in distant, yet connected brain regions, a phenomenon known as diaschisis (Carrera and Tononi, 2014). In a similar pattern, subcortical stroke lesions have been shown to induce structural degeneration of remote, yet connected white and grey matter, most likely due to retrograde, anterograde as well as transneuronal degeneration (Duering *et al.*, 2015; Cheng *et al.*, 2019).

In recent years, growing evidence supports the understanding of the brain as a complex network of interconnected areas, the structural basis of which can be described by the structural "connectome" (Bullmore and Sporns, 2009). This comprehensive map of neuronal connections reconstructs the brain as a network based on two principle components: Nodes, which represent a pre-specified cortical areas; and edges, representing the interconnecting white matter tracts. In this abstract form, the connectome can be described by mathematical approaches such as graph theory (Rubinov and Sporns, 2010). By this approach, we gain insight into organizational properties of the brain structure, its configuration and topology. A growing number of studies demonstrate that nodes and edges in the connectome are not organized in a random configuration. Rather, structural connections are arranged in a balanced state that supports functional segregation between different specialized brain areas but also allows for their functional integration (Sporns, 2013). These aspects of network topology can be captured by specific graph theoretical measures such as the global efficiency, clustering and modularity (Rubinov and Sporns, 2010; Aerts *et al.*, 2016).

Focal stroke lesions disrupt the large scale network of interconnected brain areas distant from the lesion side as specific form of "connectomal" diaschisis (Carrera and Tononi, 2014). There

For Review Only

is evidence for widespread alterations of functional brain connectivity in the human connectome after stroke studied by EEG and functional magnetic resonance imaging. However studies of structural changes in large scale, whole brain networks after stroke are still scarce (Carter *et al.*, 2012; Aerts *et al.*, 2016). Specifically, there are few reports on the effect of focal stroke lesions on structural brain networks at the contralesional hemisphere. Therefore, we aim to reconstruct and analyze structural connectomes after stroke both in the ipsi- and contralesional hemisphere. For this aim, we examine patients with upper limb motor deficits as a common and relevant clinical deficit in the chronic stage after stroke. We reconstruct the structural connectome based on diffusion weighted imaging and probabilistic fibre tracking algorithm. We apply graph theoretical tools to connectome data to illustrate topological changes in comparison to healthy participants of comparable age. We hypothesize that at the ipsilesional hemisphere; topological measures of the connectome reflect a connectivity deficit as well as a disturbed configuration of structural networks. In addition, we aim to demonstrate changes in connectivity and network topology in the contralesional hemisphere that occur in the wake of structural diaschisis or adaptive and compensatory plasticity. Lastly, studying patients with motor deficits, we hypothesize that global and local graph parameters relate to clinical measures of motor impairment.

For Review Only

Materials and methods

Subjects

For this cross-sectional observational study, patients with first time, singular, supratentorial ischemic stroke and upper limb motor deficits in the chronic stage (more than six months since stroke onset) were recruited from our university hospital. Exclusion criteria included contraindications to magnetic resonance imaging, aphasia or hemianopia rendering patients unable to give written informed consent or participate in clinical evaluations as well as presence of neurological or psychiatric comorbidities. Motor deficits were quantified by means of whole

handgrip force and the Fugl-Meyer assessment of the upper extremity (UEFM), which quantifies active movement range and synergies of upper extremity muscles on an ordinal scale. For grip strength testing, grip force of both hands (affected and unaffected) was measured three consecutive times using a hand dynamometer (Strength JAMAR hand evaluation kit, Elite healthcare, UK) and the average of three measurements was taken for further analysis. Clinical evaluation and testing of motor deficits were performed prior to MR imaging, all participants were scanned by MRI on the same day as clinical testing. Demographic data (gender, age and days since stroke onset) was recorded. In addition to patients, healthy participants of comparable age and gender were recruited. The study was approved by the local ethics committee. All participants gave written informed consent according to the Declaration of Helsinki.

Imaging

Diffusion-weighted and high-resolution T_1-weighted anatomical images were acquired using a 3 Tesla Siemens Skyra MRI scanner (Siemens, Erlangen, Germany). For the former, 75 axial slices were obtained covering the whole brain with gradients (b = 1500 s/mm^2) applied along 64 noncollinear directions with the following sequence parameters: repetition time (TR) = 10 000 ms, echo time (TE) = 82 ms, field of view (FOV) = 256 × 204, slice thickness (ST) = 2 mm, in-plane resolution (IPR) = 2 × 2 mm. For the latter, a three-dimensional magnetization-prepared, rapid acquisition gradient-echo sequence (MPRAGE) was used with the following parameters: TR = 2500 ms, TE = 2.12 ms, FOV = 256 × 208 mm, 256 axial slices, ST = 0.94 mm, and IPR = 0.83 × 0.83 mm.

Reconstruction of the structural brain connectome

Undirected, weighted networks were constructed based on high resolution structural imaging, diffusion tensor imaging and probabilistic tractography to approximate white matter fibre tracts as described previously (Schlemm *et al.*, 2017). In summary, diffusion-weighted images were

For Review Only

analysed using the FSL software package 5.1 (http://www.fmrib.ox.ac.uk/fsl). All datasets were corrected for eddy currents and head motion. Structural T1-weighted anatomical images were processed using the FreeSurfer software package 5.3.0 with standard procedures and parameters resulting in a cortical parcellation of 34 cortical and 6 subcortical regions per hemisphere (Desikan *et al.*, 2006; Behrens *et al.*, 2007). Additionally, one brain stem region was defined manually in MNI space at the level of the pontomedullary junction comprising the area of descending motor tracts. For cortical regions, masks from automated parcellation by Freesurfer were refined to delineate the gray–white matter boundary underlying the cortical areas to increase anatomical accuracy for Diffusion Tensor Imaging (DTI) fiber tracking. Therefore, surface maps of the boundary between grey and white matter were generated by FreeSurfer and applied to constrain parcellation masks to a ribbon directly underlying the cortical grey matter. All resulting masks were visually checked for plausibility and accuracy. In total, 82 masks (41 per hemisphere) were created as listed in supplementary table S1. Stroke lesions were manually delineated on T1-weighted anatomical MR images and visually checked for plausibility and accuracy after registration to individual maps of fractional anisotropy (FA). Stroke lesion masks were applied to exclude affected brain regions from probabilistic fiber tracking. In addition, pathways were disregarded if they entered a voxel with FA < 0.15. This value was chosen based on previous studies and experience (Cheng *et al.*, 2014; Finger *et al.*, 2016) showing this threshold consistently preventing erroneous tracking between neighboring gyri.

Processing of diffusion data included application of a probabilistic diffusion model modified to allow estimation of multiple ($n = 2$) fiber directions using the program bedpostX. Probabilistic fiber tracking was carried out using the command probtrackx2 as implemented in FSL and described previously (Schlemm *et al.*, 2017). From each seed region of interest (ROI) voxel, 5000 streamlines were initiated through the probability distribution on principle fiber direction. Structural connectivity between two regions was measured by masking each seed ROI results

For Review Only

by each of the remaining ROIs. Both whole-brain and intrahemispheric fiber tracking were performed to individually create connectivity matrices including and excluding interhemispheric (transcallosal) connections. For intrahemispheric network reconstruction, tracking streamlines were discarded when they intersected the midline as defined by a manually generated exclusion mask. Weighted connectivity matrices were computed by defining the strength of the connection from ROI s to ROI t to be given by the raw number of streamlines starting in s and running through t, divided by the sum of the volumes of s and t. Network reconstruction resulted in connectivity matrices of dimension 82 x 82 and 41 x 41, for whole-brain and intrahemispheric networks, respectively, which were symmetrised by averaging with their own transpose.

For Review Only

Graph theoretical analysis of structural networks

Properties of the structural connectome were quantified on the level of global connection strength, global and local topological graph parameters as well as patterns of edgewise connectivity. For the latter two, the Brain Connectivity Toolbox (BCT, Version 2017-15-01) (Rubinov and Sporns, 2010) and the network based statistics toolbox (NBS, v1.2) (Zalesky *et al.*, 2010) where used, respectively. In detail, we first calculated median connectivity strength (q_{50}) of each network. Second, we characterized prominent topological aspects of network architectures using global graph parameters (GGP). Specifically, we chose global efficiency, clustering and modularity since these measures are robust and well suited to capture topological properties in large-scale networks. Whereas global efficiency can be viewed as a measure of network integration, global clustering and modularity reflect the element of segregation in large scale brain networks (Bullmore and Sporns, 2009; Welton *et al.*, 2015). In weighted networks, the former two are strongly influenced by both connection strength and topology (Rubinov and Sporns, 2010). Therefore, we normalised global efficiency and clustering in relation to 1000 simulated random networks with equal distribution of edge weight, node strength and degree to

increase comparability between individual connectomes as described previously (Rubinov and Sporns, 2011; Schlemm *et al.*, 2017). Furthermore, measures of network architecture are sensitive to the density at which networks are analysed (van Wijk *et al.*, 2010; Fornito *et al.*, 2013). Estimates of anatomical connection density depend on the resolution of the underlying cortical parcellation and fibre reconstruction method (Bassett and Bullmore, 2017), with values ranging from less than 30% in the binary case to 66% in a Macaque tract-tracing study (Markov *et al.*, 2013). The optimal choice of network density for the analysis of imaging-derived weighted structural connectomes therefore remains controversial (Civier *et al.*, 2019). As done previously (Seguin *et al.*, 2018; Buchanan *et al.*, 2019), weighted brain networks were therefore thresholded by multiplication by a sparsity mask corresponding to the 20%, 25%, (...), 75% and 80% strongest connections in the average network of healthy participants and analysed over this range of network densities. We computed GGP separately for all whole-brain and intrahemispheric networks (including and excluding interhemispheric transcallosal connections, respectively). Lastly, the local graph parameter node strength (Rubinov and Sporns, 2010) was calculated to quantify aspects of a network's architecture in the vicinity of a specific brain region (node) to localize changes in network structure at specific regions in the brain.

Whereas the aforementioned graph parameters characterize network topology at the level of interconnected nodes, changes in the structural connectome can also be described focusing on configuration of edges, i.e. interconnecting white matter tracts. For this purpose, we applied the network based statistics toolbox (NBS) as described previously (Zalesky *et al.*, 2010). In summary, this approach identifies and visualizes subnetworks of edges with significant differences in connection strength between patients and healthy participants. Similar to traditional cluster-based thresholding, NBS applies a two-step method that exploits the extent

For Review Only

to which edges with a large difference in connectivity are interconnected, therefore addressing the issue of family wise error rate of the set of identified edges.

A fixed proportional thresholding of 50% was applied to structural connectomes for the analysis of local network parameters. This value was chosen so as to be consistent with both recent interval estimates of the mammalian structural connectome (Bassett and Bullmore, 2017) and the peak intrahemispheric efficiency we observed in our data. A sensitivity analysis for group differences in local network architecture with respect to network density was performed.

Computational lesion model

In addition to empirically observed alterations of the structural connectome, we were interested in basic computational lesion models that could reproduce and help to interpret observed changes in network topology of chronic stroke patients. Based on the topological assumption of small world characteristics as one key feature of the human connectome (Bassett and Bullmore, 2006), we simulated the influence of stroke lesions on overall network topology using the Watts–Strogatz model which generates networks exhibiting small-worldness characteristics similar to a prominent topological feature observed structural brain networks (Bassett and Bullmore, 2006). We used the Network Generation and Analysis Toolbox implemented in Matlab (Sizemore *et al.*, 2017) to simulate n = 38 realizations of the Watts-Strogatz model (matching the total number of patients and control subjects) with 41 nodes (matching the number of regions of interest in each hemisphere). The following configuration parameters were used: p = 0.25 (probability of rewiring an edge of the ring lattice into a random short cut connection) and k = 10 (desired mean degree for each node). The latter parameter was chosen matching the average degree of the empirical brain networks at a density of $\kappa = 0.27$. Following generation of network models, we simulated the effect of virtual lesions in a paradigm that preferentially affects long-range connections. This paradigm was chosen in respect to the stroke lesion distribution in our group of patients that almost exclusively affected

subcortical regions (see Fig. 1). The resulting network disruption was then quantified by graph theoretical measures analogous to the empirical data. As before, global efficiency and clustering were normalized with respect to the average value in 1000 null models obtained from degree-, strength- and weight-preserving randomization.

Statistical Analysis

We quantified network alterations in ipsi- and contralesional hemispheres in comparison to connectomes from age- and gender-matched healthy participants in terms of global and local network properties. Median connectivity strength was analysed using a mixed effects linear model including (1) the fixed effect *side* with levels left and right; (2) the fixed effect *lesion status* with levels ipsilesional, contralesional, and healthy participants; (3) the random factor *subject.* The model was first fitted with interaction terms; in the absence of a statistically significant interaction it was refitted without interactions, followed by post hoc analyses of main effects. Effect sizes in omnibus tests were quantified as ratios of likelihoods corresponding to models with and without the factor of interest. Residuals were tested against normality using the Shapiro–Wilk test (Shapiro and Francia, 1972). For global graph parameters (GGP), effects of lesion status on efficiency, clustering and modularity were analysed over the entire range of thresholds using the multi-threshold permutation correction (MTPC) approach (Drakesmith *et al.*, 2015). This technique accounts for both the magnitude of the effect at a particular network density and its persistence across network densities and thus mitigates the multiple comparison problem associated with analysing the networks at different sparsity levels. It is thus threshold-agnostic and eliminates the necessity to pre-specify an arbitrary network density. The method was first applied to whole-brain GGP as well as left and right intrahemispheric GGP, respectively. To isolate the effect of lesion status, data were then pooled and an MTPC analysis was performed for the residuals obtained from subtracting from each data point the corresponding hemisphere- and density-specific group average. For group comparison of local

For Review Only

network properties, a mass univariate analysis was chosen. Mixed effects linear models as before were fitted for local graph parameters of individual ROIs and assessed for statistical significance of lesion status. After correction for multiple testing using the Bonferroni approach, brain regions showing altered local network characteristics were identified and subjected to post hoc tests. We investigated significant differences in white matter (edge-wise) connectivity using NBS (Zalesky *et al.*, 2010). Therefore, a range of t-values ranging from 1 to 5 was employed in the first step to detect critical differences in edgewise connectivity. A conservative t-value of 3.1, corresponding to an edge-wise significance level of $p \approx 0.0025$, which maximised the statistical significance of detected network disruptions was chosen to localize the relevant subnetwork (a more detailed description of this approach can be found in the supplementary materials). Imaging-derived measures of connectivity strength as well as global and local network topology were assessed for association with motor performance scores (grip strength and UEFM) using linear regression analysis. All statistical analyses were conducted in the R computing environment (R Core Team, 2008).

For Review Only

Data availability

Data from this study is available for researches upon reasonable request.

Results

Subjects

Table 1 displays an overview of the subjects included in this study. A total of 17 patients were recruited (3 female). Mean age was 64.5 ± 8.4 years, 8 (47 %) patients had lesions in the left hemisphere. In addition, 21 healthy participants with comparable distributions of age and gender (10 females, age 64.9 ± 10.3 years) were recruited. More detailed individual values for demographic, clinical and graph theoretical data can be found in supplementary table S2. There was no statistically significant association between age and disease status ($F_{2,35} = 0.009$, p =

0.999) or gender and disease status (χ_2 = 3.287, simulated p = 0.219). Lesion distribution is illustrated in Fig. 1 with lesion volume ranging between 0.1 ml and 3.9 ml (median 0.8 ml, IQR 0.3 – 1.7 ml). All stroke lesions were located in brain areas supplied by the middle cerebral artery (MCA) involving the basal ganglia and subcortical white matter. In a minority of patients, cortical insular regions were affected. Primary sensory and motor cortices were unaffected by stroke lesions in all patients. Analysis of motor scores indicated a deficit in grip strength at the affected compared to the unaffected hand (Cohen's d=1.06, p=0.004). There was no significant association between lesion volume and grip strength of the affected hand (r=0.44; p=0.075) as well as UEFM (r=0.12; p=0.639).

For Review Only

Brain network measures and graph theoretical analysis

We investigated three aspects of structural brain networks in association with clinical parameters to capture the integrity and topological changes of the structural connectome during the chronic stage after stroke. Therefore, we applied measures from graph theory that quantify the degree of overall connectivity, network organization (topology) and localization of pronounced network changes in specific anatomical brain areas.

First, overall connectivity of structural brain networks was quantified by means of median connectivity strength and compared to healthy participants as are shown in Fig. 2. Median connectivity strength q_{50} was different between hemispheres of healthy participants, contralesional hemispheres and ipsilesional hemispheres (Λ_2 = 16.933, p < 0.001). Post hoc tests detailed in Table 2 indicated overall reduced connection strength in ipsilesional hemispheres compared to both contralesional hemispheres and hemispheres of healthy participants. A trend of lower connection strength was also detected in contralesional hemispheres compared to healthy participants although this did not reach statistical significance. Inclusion of age and sex as cofactors did not essentially alter the results of this analysis (supplementary table S3). There was no evidence for a significant difference in global

connectivity between left and right hemispheres (q_{50}: $F_{1, 37} = 0.112$, $p = 0.739$; λ: $F_{1, 37} = 0.865$, $p = 0.358$) or a significant interaction between lesion status and side of the lesion (q_{50}: $\Lambda_2 = 0.779$, $p = 0.677$; λ: $\Lambda_2 = 2.702$, $p = 0.259$). No statistically significant associations between global connectivity and grip strength of the affected hand or the UEFM were observed (see supplemental table S4 for details).

Second, we investigated topological changes of the connectome after stroke that can be considered as the layout and organisation of structural brain networks that is not captured by measures of connection strength alone. These topological features are described by measures of graph theory that indicate the degree of integration and segregation in structural networks (Bullmore and Sporns, 2009; Welton *et al.*, 2015). As described in the methods section, we chose global efficiency, clustering and modularity to capture these aspects, results are illustrated in Fig. 3. In the analysis of whole brain networks (Fig. 3A), normalized global efficiency was reduced in connectomes of patients with both left- and right-sided lesions compared to brain networks of healthy participants. Normalized global clustering and modularity were increased in stroke patients compared to normal participants with a more pronounced effect in right-sided lesions. In the separate analysis of intrahemispheric networks excluding interhemispheric connections (Fig. 3B), we observed similar results. Normalized global efficiency was reduced equally in both ipsi- and contralesional hemispheres, whereas the increase in clustering, while present both ipsi- and contralesionally, was more pronounced in directly affected ipsilesional hemispheres. For both measures, the effects were independent from hemisphere side (left and right). Intrahemispheric modularity was increased ipsilesionally in patients with right-sided stroke and unchanged in contralesional hemispheres. Details of the statistical analysis are reported in supplementary table S5.

Third, we aimed to localize the reported changes of network connective and topology to specific anatomical brain regions using local graph theoretical parameters and network based statistical

For Review Only

mapping (NBS). As shown in Fig. 4, we identified five brain regions with significant changes in local node strength compared to healthy participants based on an analysis by mass univariate linear mixed effects modelling. Local node strength was significantly reduced in ipsilesional hemispheres compared to contralesional hemispheres and healthy participants and most prominently reduced in the post- and precentral cortices as well as the thalamus, pallidum and putamen (after Bonferroni correction for multiple comparisons). There were no significant interactions of lesion status with hemisphere side. These results were stable across a range of network densities imposed by proportional thresholding (Fig S1). As an alternative and complementary approach, we applied network-based statistics to identify connections and subnetworks that are significantly altered in our group of patients. Results are shown in Fig. 5. The resulting subnetwork comprises edges with significantly lower connectivity in the ipsilesional hemisphere compared to healthy participants. These include cortico-subcortical white matter tracts between the superior temporal gyrus, pre- and postcentral gyrus, insular cortex, caudate nucleus thalamus, putamen and pallidum. Results over a wider range of initial t-thresholds are shown in supplementary Fig. S2 and S3.

For Review Only

Simulated network lesion model

Fig. 6 illustrates the effect of simulating focal lesions in a computational network model with prominent small world properties. Removing a proportion ($q = 0.5$) of long-range connections from the network architecture resulted in an overall and significant decrease of normalized global efficiency as well as an increase of normalized global clustering and modularity.

Discussion

In our study, comprehensive analysis of structural brain networks in chronic stroke patients yielded two major results: Firstly, significantly reduced structural connectivity was apparent in

the ipsilesional hemisphere. Secondly, graph theoretical analysis revealed a disturbed topology of structural brain networks in both ipsi- and contralesional hemispheres characterized by lower integration and higher segregation. These topological alterations were apparent from overall decreased global efficiency (as a measure of integration) and increased global clustering and modularity (as measures of network segregation) in structural connectomes of stroke patients compared to healthy participants. We did not detect associations with clinical parameters of motor impairment with global or local graph theoretical measures.

Rapid and persisting disruption of structural brain connectivity is the key characteristic of ischemic stroke underlying clinical deficits. In our group of patients in the chronic stage after stroke, these effects were apparent in marked overall reduction of white matter connectivity at the ipsilesional hemisphere. In terms of a more localized, network-based analysis, these changes were most prominently shown affecting nodes (i.e. brain regions) involved in the control and execution of motor functions. Local node strength, a measure of nodal connectivity, was significantly reduced in the pre- and postcentral cortex, superior and inferior parietal cortical areas as well as the basal ganglia (Fig. 4). Whereas altered connectivity of subcortical regions directly affected by the stroke lesions are self-evident, altered nodal graph measures in primarily unaffected brain regions (such as the pre- and postcentral cortex) can be explained by structural disconnections of cortical areas after stroke. In accordance with this mechanism, separate analysis of edge wise connection strength by network-based statistics (NBS) detected reduced structural integrity involving connections in a cortico-subcortical subnetwork surrounding the focus of stroke lesions in our patients (Fig. 5).

Beyond the extent of reduced global connectivity in the ipsi- and contralesional hemisphere, we characterized changes of large-scale network topology at the chronic stage after stroke. Specifically, we were interested in structural network measures that promote integration and segregation of brain function that are known to be altered in various neurological diseases that

For Review Only

are not captured directly by measures of connectivity strength (Bassett and Bullmore, 2009). For this purpose, we have chosen the parameters of global efficiency, clustering and modularity as established measures of brain network topology in various neurological diseases and to facilitate comparability of our results with previous studies (Aerts *et al.*, 2016). To account for the influence of connection strength on these measures, we aimed to establish the significance of network measures by comparison with statistics calculated on null hypothesis networks (Albert and Barabási, 2002). Our results show that in the chronic stage after stroke, structural brain networks were characterized by lower global efficiency and increased global clustering compared to healthy, age-matched participants. These changes were robust and consistent over various network densities and affected the ipsilesional as well as contralesional hemisphere independent of lesion side. Changes in normalized modularity were characterized by higher values at the ipsilesional hemispheres compared to healthy participants. Taken together, these results point toward an altered topology of the structural connectome with a lower potential to integrate functional activity between various, distant brain areas. On the contrary, structural networks of stroke patients were characterized by increases of localized, clustered and small-scale subnetworks. Regarding our group of patients with mainly subcortical stroke lesions, we hypothesize that these changes in network topology are best explained by ischemic injury and consecutive disintegration of long white matter fibre tracts interconnecting distant cortical and subcortical areas. To further investigate this hypothesis, we applied an established, basic mathematical network model (Watts-Strogatz) simulating characteristic aspects of brain network topologies, specifically small world properties (Fig. 6). We found that a paradigm with selective loss of long distant connections led to changes in global efficiency, clustering and modularity analogous to observations in the ipsilesional hemisphere of stroke patients. Application of this simplified model illustrates how single, strategically localized lesions can lead to the "break down" of efficient network architecture observed in our stroke patients. Although the network model chosen in our study reflects on basic (yet fundamental) properties

For Review Only

of neuronal networks, it remains oversimplified and does not account for the far more complex configuration of the human connectome. Therefore, comprehensive, large-scale models and simulations, optimally in native brain space are needed that would exceed the purpose of this study.

Contrary to changes of post-stroke global functional brain connectivity, reports on alterations in large scale, whole-brain structural connectomes after stroke are scarce. In a previous topological network analysis, "communicability" as a localized, nodal measure of efficient information transfer was significantly reduced in structural connectomes of nine patients in the early chronic stage (4-5 weeks) after stroke (Crofts *et al.*, 2011). Compared to age-matched healthy controls, changes were most prominent ipsilesional and in regions surrounding the stroke lesion, comparable to our observations in the ipsilesional hemisphere by nodal and edgewise network analysis. In addition, classification algorithms separated patients from healthy participants based on lower communicability values in homologous brain regions at the contralesional hemispheres. In contrast to findings from our study, global graph measures such as efficiency and clustering were not significantly divergent between patients and healthy controls. This is potentially due to the significantly larger interval after stroke in our group of patients, as structural white matter disintegration is known to evolve over time and become more prominent and more easily detected by structural imaging modalities such as DTI. Focussing on changes of the contralesional hemisphere, a recent experimental animal study applied ultra-high resolution diffusion MRI to reconstruct structural brain networks 70 days after experimental stroke in post-mortem rat brains (Sinke *et al.*, 2018). Graph theoretical measures were calculated at contralesional hemisphere using a minimum spanning tree algorithm to detect relevant changes of network topology, specifically the "back bone" of fundamental structural connections. As a result, several topological features of contralesional brain networks were associated with sensory-motor deficits. Specifically, high network

For Review Only

eccentricity (longer path lengths between nodes in a spanning tree) was associated with worse performance in tests of sensory and motor behaviour. Longer path lengths between nodes is also the underlying base for a decrease of global efficiency. Thus, these results from experimental stroke models would be in line with our observations of decreased global efficiency in chronic stroke patients. Taken together, these results point towards alterations of structural network topology at the contralesional hemisphere that is not directly affected by a stroke. Our results also indicate a globally reduced structural connectivity in the contralesional hemisphere, which, however, did not reach statistical significance in post hoc testing. At the level of network topology, significant changes were apparent with structural connectome topology being shifted to a less optimal configuration with lowered ability to integrate various brain regions segregated into a higher number of clusters with lesser degree of long distance interconnections. In terms of a conceptual framework of complex networks, these changes signify an alteration from the proposedly optimal "small world" configuration towards a more random topology involving both hemispheres after stroke (Aerts *et al.*, 2016). Analogous to the underlying damage to white matter tracts at the ipsilesional hemisphere, we propose that degeneration of long range, white matter tracts is the primary pathophysiological mechanism of disturbed network topology in contralesional hemispheres of stroke patients. Distant effects of stroke lesions at the contralesional hemisphere were previously reported in terms of white and grey matter disintegration in contralesional hemispheres after stroke. (Koliatsos *et al.*, 2004; Avanzino *et al.*, 2007; Schaechter *et al.*, 2009; Cheng *et al.*, 2019). Although the primary mechanism of these effects at the distance of stroke lesions are still to be elucidated, they are most likely induced by transneuronal mechanisms via the degeneration of interneurons at the cerebral cortex and transcallosal fibers subsequently resulting in apoptosis of contralesional white matter tracts. However, other factors, such as diffusely distributed cortical hypometabolism found in previous studies of diaschisis after subcortical stroke at the contralesional hemisphere are potential contributors (Kushner *et al.*, 1984; Pappata *et al.*, 1990).

For Review Only

Taken together, our observations demonstrate the impact of focal lesions on contralesional connectomes as a form of "Connectomal Diaschisis" in analogy to the well described effects of ipsilateral lesions to distant brain structure and function after stroke (Carrera and Tononi, 2014).

In our study, we focussed on patients with upper limb deficits that have a major impact on daily activities and occur frequently in stroke populations. In addition, recovery of upper extremity function after stroke has been extensively studied by structural MRI studies highlighting brain structures crucial for clinical outcome such as the pyramidal tract or connected primary and secondary motor areas. Disturbances of structural organization in the chronic phase after stroke were not associated with severity of clinical motor impairment as measured by grip strength and the UEFM. This is in contrast to several neurological diseases where alterations of the structural brain network topology were related to the degree of clinical deficits (Aerts *et al.*, 2016). Lack of clinical implications in our study might stem from the relatively small patient number or long time interval after stroke, were adaptive mechanisms in brain function have led to rehabilitation of initial motor impairment "despite" extensive damages to structural brain networks (Aerts *et al.*, 2016).

Several limitations of our study have to be mentioned. Considering the cross-sectional design of our study and lack of longitudinal data, we cannot conclude that the observed alterations in structural network integrity and topology are solely caused by the localized stroke lesion. Although the observed changes are pathophysiologically plausible and reproducible in a basic network simulation model, it is conceivable that they are influenced by other premorbid risk factors for cerebrovascular diseases such as hypertension or diabetes commonly present in stroke patients. Since we did not systematically record these potentially confounding factors, further studies applying a multivariate analysis and longitudinal design are needed to elucidate the significance of potential pre-morbid vulnerabilities of brain networks in stroke patients. In terms of adaptive mechanisms, changes of network measures such as increased

For Review Only

communicability have been found in the two previous studies mentioned above and discussed to indicate structural plasticity after stroke (Crofts *et al.*, 2011; Sinke *et al.*, 2018). In our study, we did not observe increases in structural connectivity or topological changes that would indicate adaptive improvements in network function, most likely due to the cross-sectional design of our study. Given the sample size of 17 patients, it was necessary to restrict attention to a small number of well-established network measures in order to minimise the risk of overfitting. Results should therefore be confirmed and extended to more subtle network properties in larger cohorts of stroke patients. Further studies including data from functional brain imaging, are needed to clarify the extent and role of compensatory structural network changes after stroke. Lastly, due to the pre-specified phenomenological selection of stroke patients in our study, we are unable to draw conclusions as to the generalizability of our results to stroke patients demonstrating different lesion patterns (i.e. mainly involving cortical areas).

In summary, we demonstrate that stroke lesions affect not only structural integrity, but also topology of the human structural connectome. Our findings suggest that alterations in white matter structure, primarily of long fiber tracts connecting distant brain areas, influence network measures of efficiency of communication in both hemispheres following stroke. Further studies are needed to investigate the functional relevance of disturbed optimal balance between regional segregation and interregional integration in structural connectomes after stroke.

For Review Only

Funding

This research was supported from the German Research Foundation (DFG) SFB-936 Multi-site Communication in the Brain (projects A1, C1 and C2).

Competing interests

All authors declare that there are no competing interests.

For Review Only

References

Aerts H, Fias W, Caeyenberghs K, Marinazzo D. Brain networks under attack: Robustness properties and the impact of lesions. Brain 2016; 139: 3063–3083.

Albert R, Barabási A-L. Statistical mechanics of complex networks. Rev Mod Phys 2002; 74: 47–97.

Avanzino L, Teo JTH, Rothwell JC. Intracortical circuits modulate transcallosal inhibition in humans. J Physiol 2007; 583: 99–114.

Bassett DS, Bullmore E. Small-world brain networks. Neuroscientist 2006; 12: 512–23.

Bassett DS, Bullmore ET. Human brain networks in health and disease. Curr Opin Neurol 2009; 22: 340–347.

Bassett DS, Bullmore ET. Small-World Brain Networks Revisited. Neuroscientist 2017; 23: 499–516.

Behrens TEJ, Berg HJ, Jbabdi S, Rushworth MFS, Woolrich MW. Probabilistic diffusion tractography with multiple fibre orientations: What can we gain? Neuroimage 2007; 34: 144–155.

Buchanan CR, Bastin ME, Ritchie SJ, Liewald DC, Madole J, Tucker-Drob EM, et al. The effect of network thresholding and weighting on structural brain networks in the UK Biobank. bioRxiv 2019: 649418.

Bullmore E, Sporns O. Complex brain networks: Graph theoretical analysis of structural and functional systems. Nat Rev Neurosci 2009; 10: 186–198.

Carrera E, Tononi G. Diaschisis: Past, present, future. Brain 2014; 137: 2408–2422.

Carter AR, Shulman GL, Corbetta M. Why use a connectivity-based approach to study stroke

For Review Only

and recovery of function? Neuroimage 2012; 62: 2271–2280.

Cheng B, Braass H, Ganos C, Treszl A, Biermann-Ruben K, Hummel FC, et al. Altered intrahemispheric structural connectivity in Gilles de la Tourette syndrome. NeuroImage Clin 2014; 4: 174–181.

Cheng B, Dietzmann P, Schulz R, Boenstrup M, Krawinkel L, Fiehler J, et al. Cortical atrophy and transcallosal diaschisis following isolated subcortical stroke. J Cereb Blood Flow Metab 2019: 0271678X1983158.

Civier O, Smith RE, Yeh C-H, Connelly A, Calamante F. Is removal of weak connections necessary for graph-theoretical analysis of dense weighted structural connectomes from diffusion MRI? Neuroimage 2019; 194: 68–81.

Crofts JJJ, Higham DJJ, Bosnell R, Jbabdi S, Matthews PMM, Behrens TEJEJ, et al. Network analysis detects changes in the contralesional hemisphere following stroke. Neuroimage 2011; 54: 161–169.

Desikan RS, Ségonne F, Fischl B, Quinn BT, Dickerson BC, Blacker D, et al. An automated labeling system for subdividing the human cerebral cortex on MRI scans into gyral based regions of interest. Neuroimage 2006; 31: 968–980.

Drakesmith M, Caeyenberghs K, Dutt A, Lewis G, David AS, Jones DK. Overcoming the effects of false positives and threshold bias in graph theoretical analyses of neuroimaging data. Neuroimage 2015; 118: 313–333.

Duering M, Righart R, Wollenweber FA, Zietemann V, Gesierich B, Dichgans M. Acute infarcts cause focal thinning in remote cortex via degeneration of connecting fiber tracts. Neurology 2015; 84: 1685–1692.

Finger H, Bönstrup M, Cheng B, Messé A, Hilgetag C, Thomalla G, et al. Modeling of Large-

For Review Only

Scale Functional Brain Networks Based on Structural Connectivity from DTI: Comparison with EEG Derived Phase Coupling Networks and Evaluation of Alternative Methods along the Modeling Path. PLOS Comput Biol 2016; 12: e1005025.

Fornito A, Zalesky A, Breakspear M. Graph analysis of the human connectome: Promise, progress, and pitfalls. Neuroimage 2013; 80: 426–444.

Koliatsos VE, Dawson TM, Kecojevic A, Zhou Y, Wang Y-F, Huang K-X. Cortical interneurons become activated by deafferentation and instruct the apoptosis of pyramidal neurons. Proc Natl Acad Sci 2004; 101: 14264–14269.

Kushner M, Alavi A, Reivich M, Dann R, Burke A, Robinson G. Contralateral cerebellar hypometabolism following cerebral insult: A positron emission tomographic study. Ann Neurol 1984; 15: 425–434.

Markov NT, Ercsey-Ravasz M, Lamy C, Ribeiro Gomes AR, Magrou L, Misery P, et al. The role of long-range connections on the specificity of the macaque interareal cortical network. Proc Natl Acad Sci U S A 2013; 110: 5187–92.

Pappata S, Mazoyer B, Dinh ST, Cambon H, Levasseur M, Baron JC. Effects of capsular or thalamic stroke on metabolism in the cortex and cerebellum: A positron tomography study. Stroke 1990; 21: 519–524.

R Core Team. Computational Many-Particle Physics. Berlin, Heidelberg: Springer Berlin Heidelberg; 2008

Rubinov M, Sporns O. Complex network measures of brain connectivity: Uses and interpretations. Neuroimage 2010; 52: 1059–1069.

Rubinov M, Sporns O. Weight-conserving characterization of complex functional brain networks. Neuroimage 2011; 56: 2068–2079.

For Review Only

Schaechter JD, Fricker ZP, Perdue KL, Helmer KG, Vangel MG, Greve DN, et al. Microstructural status of ipsilesional and contralesional corticospinal tract correlates with motor skill in chronic stroke patients. Hum Brain Mapp 2009; 30: 3461–74.

Schlemm E, Cheng B, Fischer F, Hilgetag C, Gerloff C, Thomalla G. Altered topology of structural brain networks in patients with Gilles de la Tourette syndrome. Sci Rep 2017; 7: 10606.

Seguin C, van den Heuvel MP, Zalesky A. Navigation of brain networks. Proc Natl Acad Sci U S A 2018; 115: 6297–6302.

Shapiro SS, Francia RS. An Approximate Analysis of Variance Test for Normality. J Am Stat Assoc 1972; 67: 215–216.

Sinke MR, Otte WM, van Meer MP, van der Toorn A, Dijkhuizen RM. Modified structural network backbone in the contralesional hemisphere chronically after stroke in rat brain. J Cereb Blood Flow Metab 2018; 38: 1642–1653.

Sizemore A, Giusti C, Bassett DS. Classification of weighted networks through mesoscale homological features. J Complex Networks 2017; 5: 245–273.

Sporns O. Network attributes for segregation and integration in the human brain. Curr Opin Neurobiol 2013; 23: 162–171.

Welton T, Kent D a, Auer DP, Dineen R a. Reproducibility of Graph-Theoretic Brain Network Metrics: A Systematic Review. Brain Connect 2015; 5: 193–202.

van Wijk BCMM, Stam CJ, Daffertshofer A. Comparing brain networks of different size and connectivity density using graph theory. PLoS One 2010; 5: e13701.

Zalesky A, Fornito A, Bullmore ET. Network-based statistic: identifying differences in brain networks. Neuroimage 2010; 53: 1197–207.

For Review Only

Tables

	Patients (n=17)	Controls (n=21)	p-value
**Female (%)**	13 (76.5)	10 (47.6)	p=0.140
**Mean age [years] (+/- SD)**	64.5 (+/- 8.4)	64.9 (+/- 10.3)	p=0.919
**Lesion on the left (%)**	8 (47.1)	-	
**Days since stroke (median, IQR)**	377 (363, 610)	-	
**Delta grip strength [kg](median, IQR)**	5.66 (4.00, 13.00)	-	
**UEFM (median, IQR)**	66 (60, 66)	-	

Table 1: Demographic and clinical details

Baseline characteristics of stroke patients and healthy controls included in the study. p-values are based on group comparison by unpaired t-tests.

For Review Only

	Group and lesion status			Test statistic
	Controls	Ipsilesional	Contralesional	
**Any hemisphere**	1.56 ± 0.08[a]	1.05 ± 0.08	1.47 ± 0.09[a]	$F_{2,36.65} = 9.29$; $p = 0.001$
**Left Hemisphere**	1.60 ± 0.11[a]	1.07 ± 0.13	1.41 ± 0.10[a]	$F_{2,35} = 4.02$; $p = 0.027$
**Right Hemisphere**	1.53 ± 0.11[a]	1.03 ± 0.10	1.54 ± 0.176[a]	$F_{2,35} = 4.05$; $p = 0.026$

Table 2: Median connectivity strength (q_{50}) in intrahemispheric connectomes. Test statistics in the last column represent the effect of lesion status (control, ipsilesional, contralesional). Identical superscripts within a row indicate the absence of a statistically significant difference in post hoc mean-separation testing. See also Fig. 2 for illustration.

For Review Only

Figure legends

Figure 1: Lesion overlay. Overlay plot illustrating the stroke lesion distribution of all patients (n=17), slice numbers represent z coordinates correspond to the standard MNI152 brain template. Number of affected patients is represented by colour. For illustrative purposes, all lesions were flipped to the same hemisphere.

For Review Only

Figure 2: Global intrahemispheric connectivity strength q_{50} stratified by lesion status. Boxplots show median values. Diamonds (grey colour) mark the group mean in healthy controls (blue), contralesional (green) and ipsilesional (red) hemispheres. Circles and triangles indicate left and right hemispheres, respectively. Individual contrasts with a statistical significance exceeding $\alpha=0.05$ are indicated by brackets. *** $p<0.0005$

Figure 3: Global graph parameters (GGP) as functions of network density. Global efficiency, clustering and modularity of whole brain (A) as well as intrahemispheric brain networks (B) for patients with lesions in the left (triangle marker) and right (circle marker) hemispheres, as well as healthy controls (box marker). Ribbons indicate standard errors of the mean. Efficiency and clustering have been normalized with respect to average values in $n = 1000$ strength-, degree- and weight-preserving random null models. (C) Residuals of hemispheric GGP after subtraction of the average values in left and right hemispheres for healthy controls (+), hemispheres affected by stroke (×), as well as contralesional hemispheres (∗). For ease of visualisation, the curves in the second row in panels (A) and (B) represent the

difference between the normalised clustering coefficients and one, multiplied by the square of the network density, i.e. $(\tilde{C}-1)\kappa^2$. Abbreviations: HC, healthy controls.

Figure 4: Connectome disruption quantified by local node strength. Height of bars indicate strength of statistical evidence from linear mixed effects modelling for main effect of lesion status (ipsilesional, contralesional, healthy control) on local node strength of each predefined region (negative logarithm of the obtained p-value). Horizontal lines correspond to significant effect in mass univariate modelling (p=0.05 and p=0.05/41 as the criticality threshold after Bonferroni correction). **Inset**: Node strength of five brain regions exhibiting a significant effect of lesions status after Bonferroni correction. Diamonds mark the group means. Individual circles and triangles indicate left and right hemispheres, respectively. Individual contrasts with a statistical significance are indicated by brackets. * $p<0.05$; ** $p<0.005$; *** $p<0.0005$.

For Review Only

Figure 5: Network based statistics (NBS) analysis. Lateral **(A)** and frontal **(B)** projections on a standard brain template. Displayed is the ipsilesional subnetwork consisting of edges with a connectivity deficit compared to healthy controls, quantified by an edgewise t-statistic exceeding a threshold value of t=3.1. Blue and yellow spheres indicate subcortical and cortical regions, respectively. To illustrate three dimensional spatial co-localizations with stroke lesion, core lesion distribution (red object) from the majority of patients is shown (11 of 17 patients, see also Fig. 1 for a two dimensional representation of stroke lesions). Abbreviations: C: caudate nucleus; Pa: pallidum; Pu: putamen; T: thalamus; IC: insular cortex; PrC: precentral cortex; PoC: postcentral cortex; SPC: superior parietal cortex.

Figure 6: Effect of lesioning long range connections in the Watts–Strogatz model. (A) Simulation of a network model using a weighted ring lattice with 41 nodes and mean degree 10. **(B)** Subsequently, a proportion of long range connections is removed, modelling the effect of a focal, e.g. ischaemic, lesion (red circle). **(C)** Resulting "lesioned" network model **(D)** Global graph parameters (GGP) efficiency, clustering coefficient, and modularity of 38 realizations (21 intact, 17 lesioned) of Watts–Strogatz networks. The former two measures were normalized with respect to n =1000strength-, degree- and weight preserving random null models. Diamonds mark the mean and brackets a significant statistical group difference. *** p< 0.0005, ***** p<0.000005

For Review Only

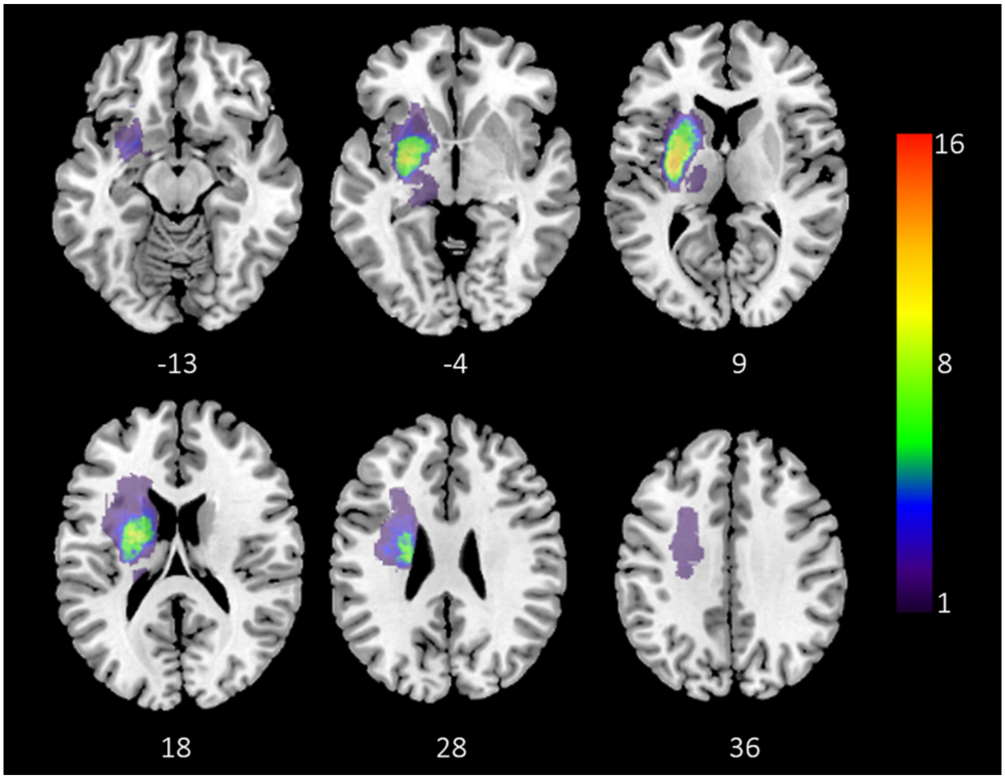

Figure 1: Lesion overlay. Overlay plot illustrating the stroke lesion distribution of all patients (n=17), slice numbers represent z coordinates correspond to the standard MNI152 brain template. Number of affected patients is represented by colour. For illustrative purposes, all lesions were flipped to the same hemisphere.

79x61mm (300 x 300 DPI)

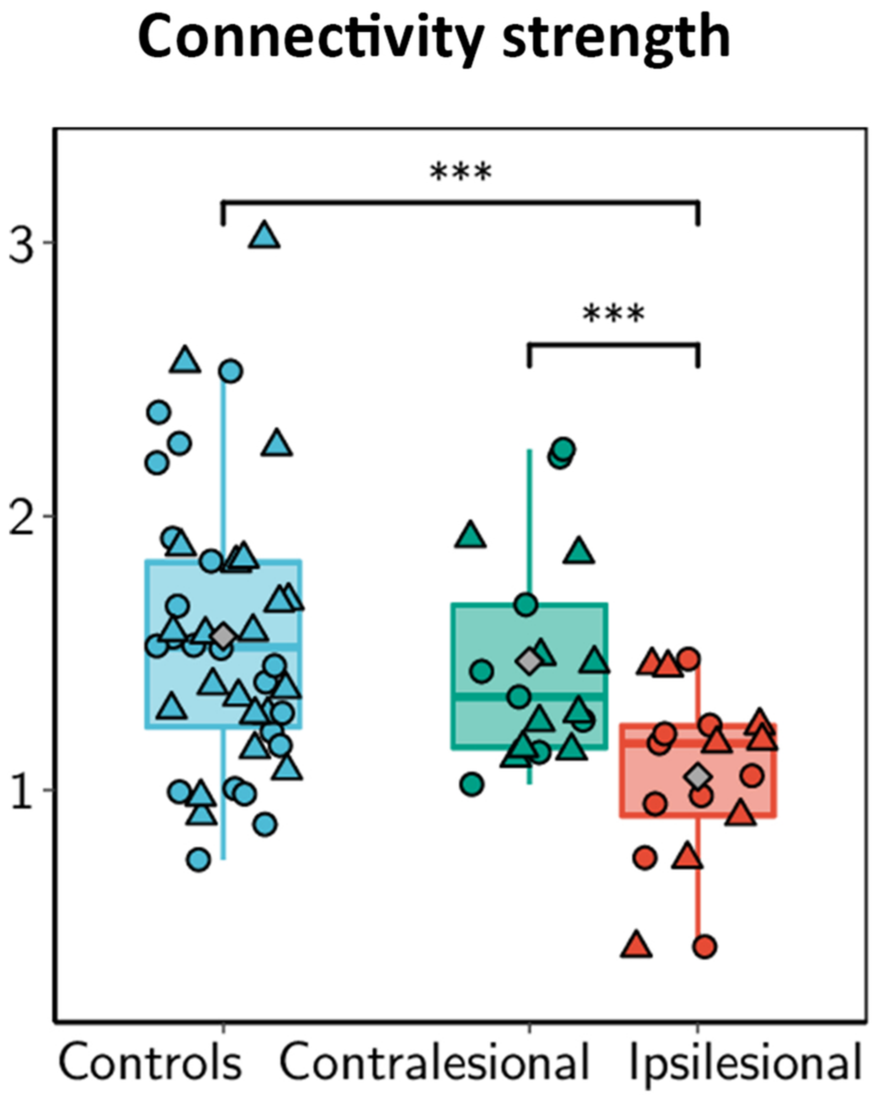

Figure 2: Global intrahemispheric connectivity strength q50 stratified by lesion status. Boxplots show median values. Diamonds (grey colour) mark the group mean in healthy controls (blue), contralesional (green) and ipsilesional (red) hemispheres. Circles and triangles indicate left and right hemispheres, respectively. Individual contrasts with a statistical significance exceeding α=0.05 are indicated by brackets. *** $p<0.0005$

79x101mm (300 x 300 DPI)

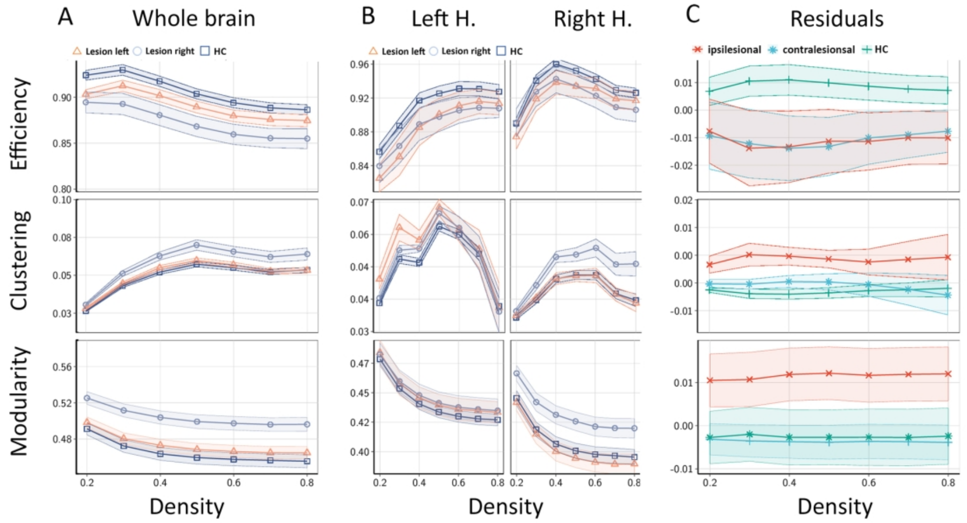

Figure 3: Global graph parameters (GGP) as functions of network density. Global efficiency, clustering and modularity of whole brain (A) as well as intrahemispheric brain networks (B) for patients with lesions in the left (triangle marker) and right (circle marker) hemispheres, as well as healthy controls (box marker). Ribbons indicate standard errors of the mean. Efficiency and clustering have been normalized with respect to average values in n=1000 strength-, degree- and weight-preserving random null models. (C) Residuals of hemispheric GGP after subtraction of the average values in left and right hemispheres for healthy controls (+), hemispheres affected by stroke (×), as well as contralesional hemispheres (∗). For ease of visualisation, the curves in the second row in panels (A) and (B) represent the difference between the normalised clustering coefficients and one, multiplied by the square of the network density, i.e. (C̃-1) κ^2. Abbreviations: HC, healthy controls.

170x89mm (300 x 300 DPI)

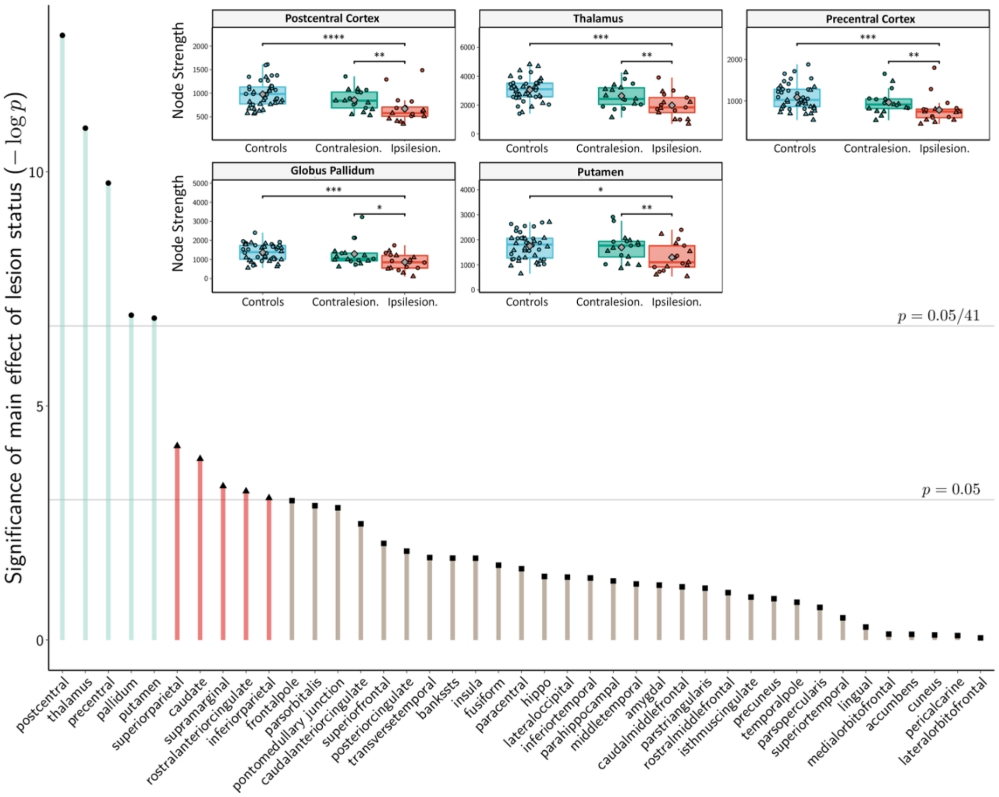

Figure 4: Connectome disruption quantified by local node strength. Height of bars indicate strength of statistical evidence from linear mixed effects modelling for main effect of lesion status (ipsilesional, contralesional, healthy control) on local node strength of each predefined region (negative logarithm of the obtained p-value). Horizontal lines correspond to significant effect in mass univariate modelling (p=0.05 and p=0.05/41 as the criticality threshold after Bonferroni correction). Inset: Node strength of five brain regions exhibiting a significant effect of lesions status after Bonferroni correction. Diamonds mark the group means. Individual circles and triangles indicate left and right hemispheres, respectively. Individual contrasts with a statistical significance are indicated by brackets. * p<0.05; ** p<0.005; *** p<0.0005.

170x136mm (300 x 300 DPI)

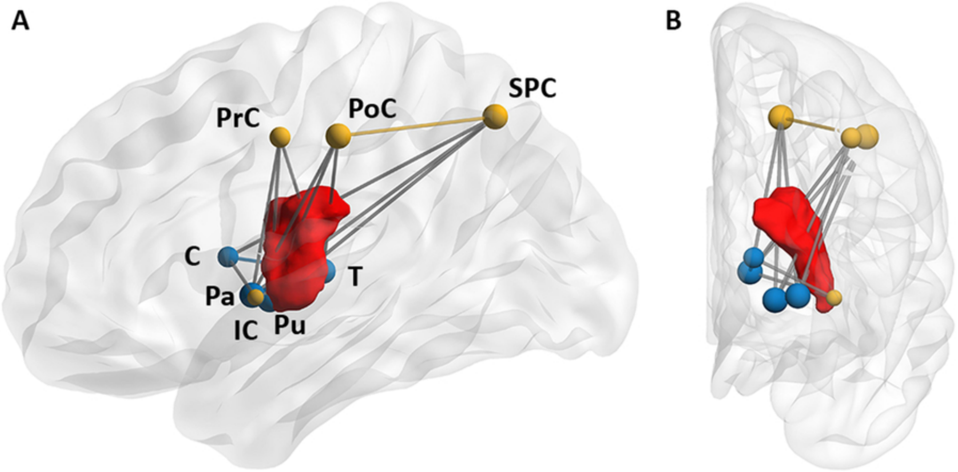

Figure 5: Network based statistics (NBS) analysis. Lateral (A) and frontal (B) projections on a standard brain template. Displayed is the ipsilesional subnetwork consisting of edges with a connectivity deficit compared to healthy controls, quantified by an edgewise t-statistic exceeding a threshold value of t=3.1. Blue and yellow spheres indicate subcortical and cortical regions, respectively. To illustrate three dimensional spatial co-localizations with stroke lesion, core lesion distribution (red object) from the majority of patients is shown (11 of 17 patients, see also Fig. 1 for a two dimensional representation of stroke lesions). Abbreviations: C: caudate nucleus; Pa: pallidum; Pu: putamen; T: thalamus; IC: insular cortex; PrC: precentral cortex; PoC: postcentral cortex; SPC: superior parietal cortex.

79x41mm (300 x 300 DPI)

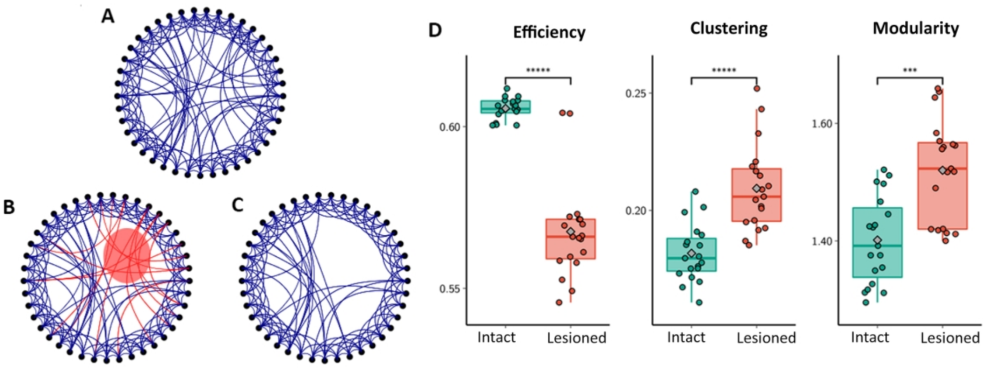

Figure 6: Effect of lesioning long range connections in the Watts–Strogatz model. (A) Simulation of a network model using a weighted ring lattice with 41 nodes and mean degree 10. (B) Subsequently, a proportion of long range connections is removed, modelling the effect of a focal, e.g. ischaemic, lesion (red circle). (C) Resulting "lesioned" network model (D) Global graph parameters (GGP) efficiency, clustering coefficient, and modularity of 38 realizations (21 intact, 17 lesioned) of Watts–Strogatz networks. The former two measures were normalized with respect to n =1000strength-, degree- and weight preserving random null models. Diamonds mark the mean and brackets a significant statistical group difference. *** p< 0.0005, ***** p<0.000005

169x64mm (300 x 300 DPI)

Altered topology of structural brain networks in chronic stroke

Supplementary Material

Bastian Cheng*[1], Eckhard Schlemm*[1], Robert Schulz[1], Marlene Boenstrup[1,2], Arnaud Messé[3], Claus Hilgetag3, Christian Gerloff[1], Götz Thomalla[1]

[1] Department of Neurology, University Medical Center Hamburg-Eppendorf, Hamburg, Germany

[2] Human Cortical Physiology and Neurorehabilitation Section, National Institute of Neurological Disorders and Stroke, National Institutes of Health, Bethesda, MD, USA

[3] Institute of Computational Neuroscience, University Medical Center Hamburg-Eppendorf, Hamburg, Germany

[*] Both authors contributed equally

Correspondence:

Bastian Cheng, MD
University Medical Center Hamburg Eppendorf
Martinistraße 52
D-20246 Hamburg
fon: 0049-40-7410-51082
fax: 0049-40--7410-57391
E-Mail: b.cheng@uke.de

For Review Only

Supplemental methods

Network-based statistics (NBS)

Network lesion mapping with the networks-based statistics toolbox requires one to specify a threshold.(Zalesky *et al.*, 2010) The optimal choice of threshold is not *a priori* clear. We therefore examined the resulting network disruptions for a range of thresholds and plotted their size (number of edges) as well as statistical significance (family-wise error rate) in supplementary Fig. S2. As expected, the sizes of NBS networks decrease with more stringent thresholds and are generally larger and more significant in ipsilesional hemispheres than in contralesional hemispheres. We also note that FEWR of contralesional NBS networks is minimised at t=1.6 which corresponds, roughly, to an edge-wise significance level of α=0.05, as determined in a separate simulation study (not shown). Network disruptions in hemispheres directly affected by stroke persist at much less liberal thresholds, with a region of relative stability around t=3.0. In supplementary Fig. S3, we show balls-and-stick models of the ipsilesional network disruptions identified around that stricter threshold, allowing an assessment of the sensitivity towards changes in the threshold and isolation of the stable core.

For Review Only

Abbreviation	Full anatomical description
accumbens	Nucleus accumbens
amygdal	Amygdala
bankssts	Banks superior temporal sulcus
caudalanteriorcingulate	Caudal anterior-cingulate cortex
caudalmiddlefrontal	Caudal middle frontal gyrus
caudate	Caudate nucleus
cuneus	Cuneus cortex
frontalpole	Frontal pole
fusiform	Fusiform gyrus
hippo	Hippocampus
inferiorparietal	Inferior parietal cortex
inferiortemporal	Inferior temporal gyrus
insula	Insula
isthmuscingulate	Isthmus–cingulate cortex
lateraloccipital	Lateral occipital cortex
lateralorbitofrontal	Lateral orbital frontal cortex
lingual	Lingual gyrus
medialorbitofrontal	Medial orbital frontal cortex
middletemporal	Middle temporal gyrus
pallidum	Globus pallidum
paracentral	Paracentral lobule
parahippocampal	Parahippocampal gyrus
parsopercularis	Pars opercularis
parsorbitalis	Pars orbitalis
parstriangularis	Pars triangularis
pericalcarine	Pericalcarine cortex
pontomedullary junction	Pontomedullary junction / Corticospinal tract
postcentral	Postcentral gyrus
posteriorcingulate	Posterior-cingulate cortex
precentral	Precentral gyrus
precuneus	Precuneus cortex
putamen	Putamen
rostralanteriorcingulate	Rostral anterior cingulate cortex
rostralmiddlefrontal	Rostral middle frontal gyrus
superiorfrontal	Superior frontal gyrus
superiorparietal	Superior parietal cortex
superiortemporal	Superior temporal gyrus
supramarginal	Supramarginal gyrus
temporalpole	Temporal pole
thalamus	Thalamus
transversetemporal	Transverse temporal cortex

For Review Only

Supplemental table S1: Cortical parcellation scheme applied for connectome generation according to the standardized FreeSurfer algorithm (Desikan *et al.*, 2006).

ID	Stroke hemisphere	Age	Gender	Time since stroke [days]	Grip force affected hand [kg]	UEFM	q50 ipsilesional	q50 contralesional
1	right	58	female	610	25	64	0.950	1.863
2	left	74	male	995	18	46	1.237	1.678
3	right	52	male	2387	13.5	37	1.479	1.921
4	left	57	male	587	39	60	1.458	2.217
5	right	71	male	803	30	49	1.172	1.491
6	left	63	male	367	33.4	66	0.906	1.139
7	left	66	male	361	33.5	66	0.750	1.256
8	right	69	male	350	47.67	66	0.754	1.119
9	right	71	male	348	37.3	66	1.053	1.281
10	right	74	female	363	25.67	66	0.429	1.145
11	left	52	male	563	42.3	66	0.426	2.245
12	right	71	male	377	34.67	66	1.238	1.154
13	right	53	male	340	44.3	66	0.980	1.463
14	right	64	male	402	18.33	60	1.206	1.249
15	left	59	female	1323	34.33	59	1.448	1.341
16	left	64	male	374	38.67	66	1.171	1.022
17	left	79	female	373	23.3	66	1.181	1.434

Supplemental table S2: Individual demographic, clinical and graph theoretical data from stroke patients included in the study. Mean connectivity strength (q50) is shown for the ipsi- and contralesional hemisphere. Abbreviations: UEFM, Fugl-Meyer assessment of the upper extremity.

For Review Only

	Lesion status			Test statistic
	Controls	Contralesional	Ipsilesional	
**Whole Brain**	1.56 ± 0.08[a]	1.47 ± 0.09[a]	1.05 ± 0.08	$F_{2,35.43}$ = 9.33, p = 0.001
**Left Hemisphere**	1.59 ± 0.11[a]	1.41 ± 0.10[a]	1.07 ± 0.12	$F_{2,33}$ = 3.81, p = 0.0324
**Right Hemisphere**	1.52 ± 0.11[a]	1.54 ± 0.16[a]	1.03 ± 0.10	$F_{2,33}$ = 4.42, p = 0.019

Supplemental table S3: Global connectivity measures corrected for covariates age and gender. Reported are median connectivity strength (q_{50}) in left and right hemispheres affected by stroke, non-affected hemispheres in stroke patients, and hemispheres from healthy controls. The test statistics in the last column represent the effect of lesion status. In the pooled analysis of left and right hemispheres in the first row, the Satterthwaite approximation is used.(Satterthwaite, 1946) Identical superscripts within a row indicate the absence of a statistically significant difference in post-hoc mean-separation testing.

For Review Only

		Grip strength	UEFM
**Lesion volume [ml]**		r = 0.4428<br>p = 0.0751	r = 0.1228<br>p = 0.6387
**Median connectivity**		r = −0.3376<br>p = 0.1851	r = −0.5452<br>p = 0.0236
**GGP**	Normalised efficiency	r = 0.1495<br>p = 0.5668	r = −0.1721<br>p = 0.509
	Normalised Clustering	r = −0.0760<br>p = 0.7718	r = −0.1285<br>p = 0.5696
	Modularity	r = −0.1210<br>p = 0.6436	r = 0.1672<br>p = 0.5327
**Nodal strength**	Precentral	r = −0.3474<br>p = 0.1718	r = −0.3043<br>p = 0.2350
	Postcentral	r = −0.2433<br>p = 0.3468	r = −0.3109<br>p = 0.2245
	Putamen	r = −0.1204<br>p = 0.6452	r = −0.3928<br>p = 0.1188
	Pallidum	r = −0.3940<br>p = 0.1176	r = −0.3260<br>p = 0.2017
	Thalamus	r = 0.0695<br>p = 0.7909	r = 0.1365<br>p = 0.6013

Supplemental table S4: associations of imaging and graph theoretical measurements with clinical data. Values for lesion volume, median connectivity strength, global graph parameters (GGP) and local node strength with significant effects of lesion status (figure 4, main manuscript) are shown. Linear correlations r between structural properties of ipsilesional stroke hemispheres and clinical characteristics. p−values are uncorrected. Abbreviations: GGP, global graph parameters; UEFM, upper extremities Fugl-Meyer Assessment of Motor Recovery.

For Review Only

	Normalized Efficiency				Normalized Clustering				Modularity			
	k*	ES	A	A_{crit}	k*	ES	A	A_{crit}	k*	ES	A	A_{crit}
**Main effect**	0.4	3.8015	13.29	3.26	0.4	4.7397	10.27	3.05	-	-	-	-
**Simple effects**												
**-Ipsilesional vs control**	0.3	-2.8390	33.47	1.13	0.4	4.8973	80.06	2.97	0.5	2.7231	39.36	5.30
**-Contralesional vs control**	0.4	-2.0216	11.24	3.08	0.2	2.3737	11.15	2.93	-	-	-	-
**-Ipsi- vs contralesional**	-	-	-	-	0.2	3.1258	27.04	2.31	0.5	2.1624	16.52	2.52

Supplemental table S5: Statistics from MTCP- Analysis (Drakesmith *et al.*, 2015)**.** Statistical details of a multi-threshold permutation correction analysis of the effect of lesion status (healthy, contralesional, ipsilesional) on intrahemispheric global graph parameters. κ^* denotes the network density with the maximal individual effect size (ES), which in turn is quantified by the value of the F and t statistic for the omnibus test and marginal contrasts, respectively. A denotes the empirical supra-critical area under curve (sc-AUC), whereas A_{crit} is the critical value of sc-AUC at a significance level of $\alpha = 0.05$ obtained from $n = 10^4$ random permutations of the empirical data.

For Review Only

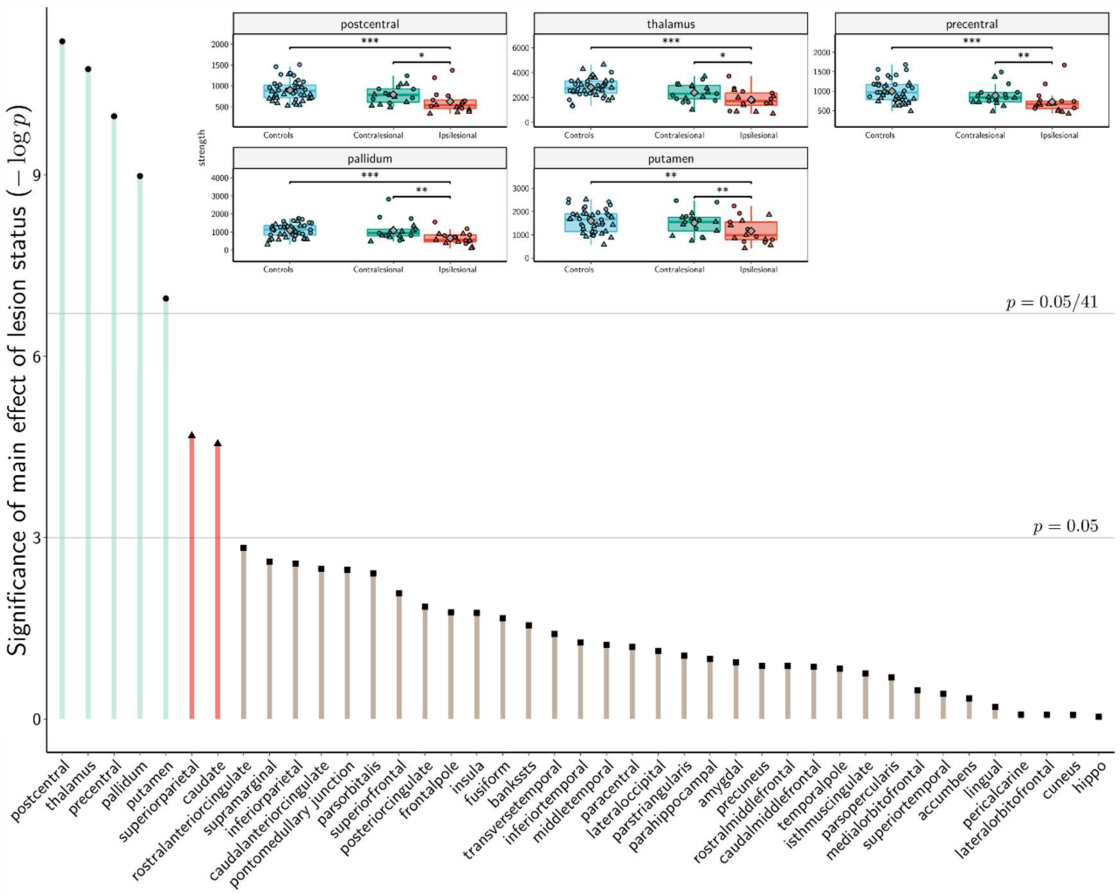
Significance of main effect of lesion status ($-\log p$)
postcentral
thalamus
precentral
pallidum
putamen
strength
Controls
Contralesional
Ipsilesional
$p = 0.05/41$
$p = 0.05$
postcentral thalamus precentral pallidum putamen superiorparietal caudate rostralanteriorcingulate supramarginal inferiorparietal caudalanteriorcingulate pontomedullary junction parsorbitalis superiorfrontal posteriorcingulate frontalpole insula fusiform bankssts transversetemporal inferiortemporal middletemporal paracentral lateraloccipital parstriangularis parahippocampal amygdal precuneus rostralmiddlefrontal caudalmiddlefrontal temporalpole isthmuscingulate parsopercularis medialorbitofrontal superiortemporal accumbens lingual pericalcarine lateralorbitofrontal cuneus hippo

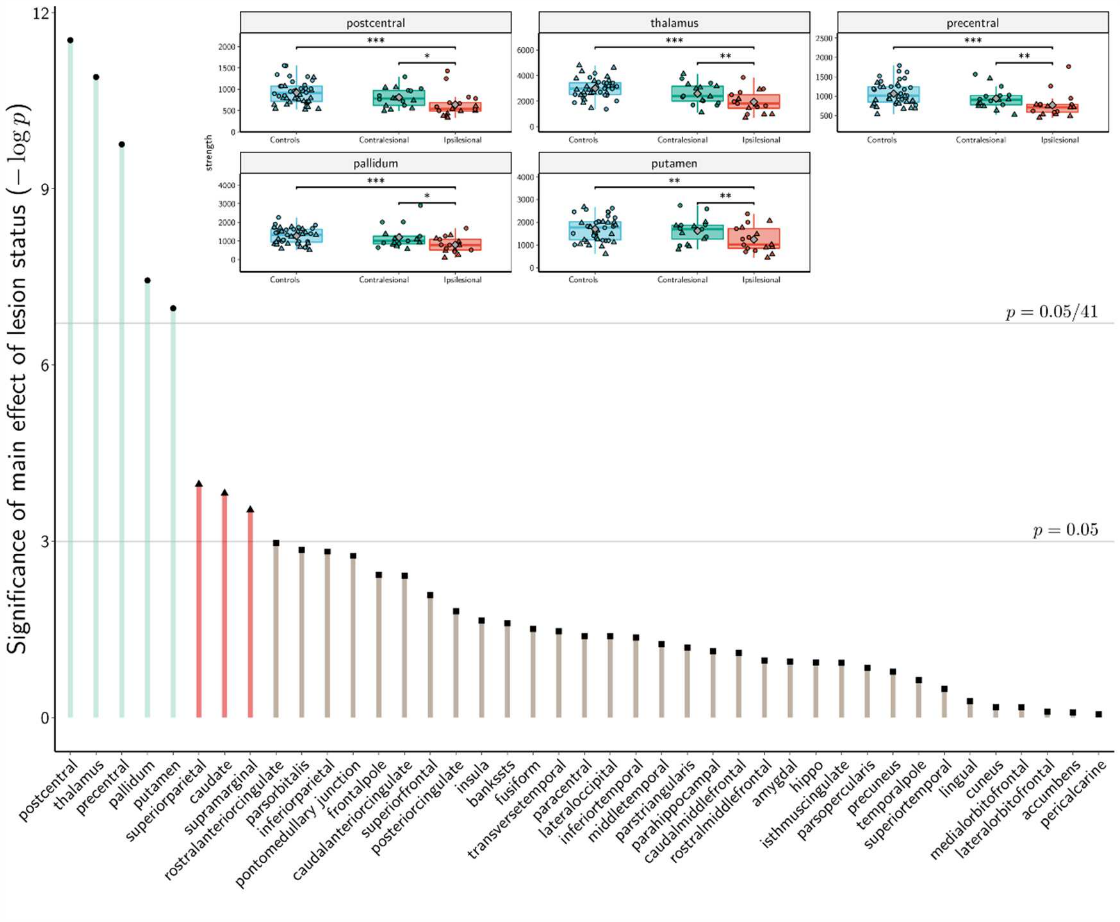
Significance of main effect of lesion status ($-\log p$)
postcentral
thalamus
precentral
pallidum
putamen
strength
Controls
Contralesional
Ipsilesional
$p = 0.05/41$
$p = 0.05$
postcentral thalamus precentral pallidum putamen superiorparietal caudate supramarginal rostralanteriorcingulate parsorbitalis inferiorparietal pontomedullary junction frontalpole caudalanteriorcingulate superiorfrontal posteriorcingulate insula bankssts fusiform transversetemporal paracentral lateraloccipital inferiortemporal middletemporal parstriangularis parahippocampal caudalmiddlefrontal rostralmiddlefrontal amygdal hippo isthmuscingulate parsopercularis precuneus temporalpole superiortemporal lingual cuneus medialorbitofrontal lateralorbitofrontal accumbens pericalcarine

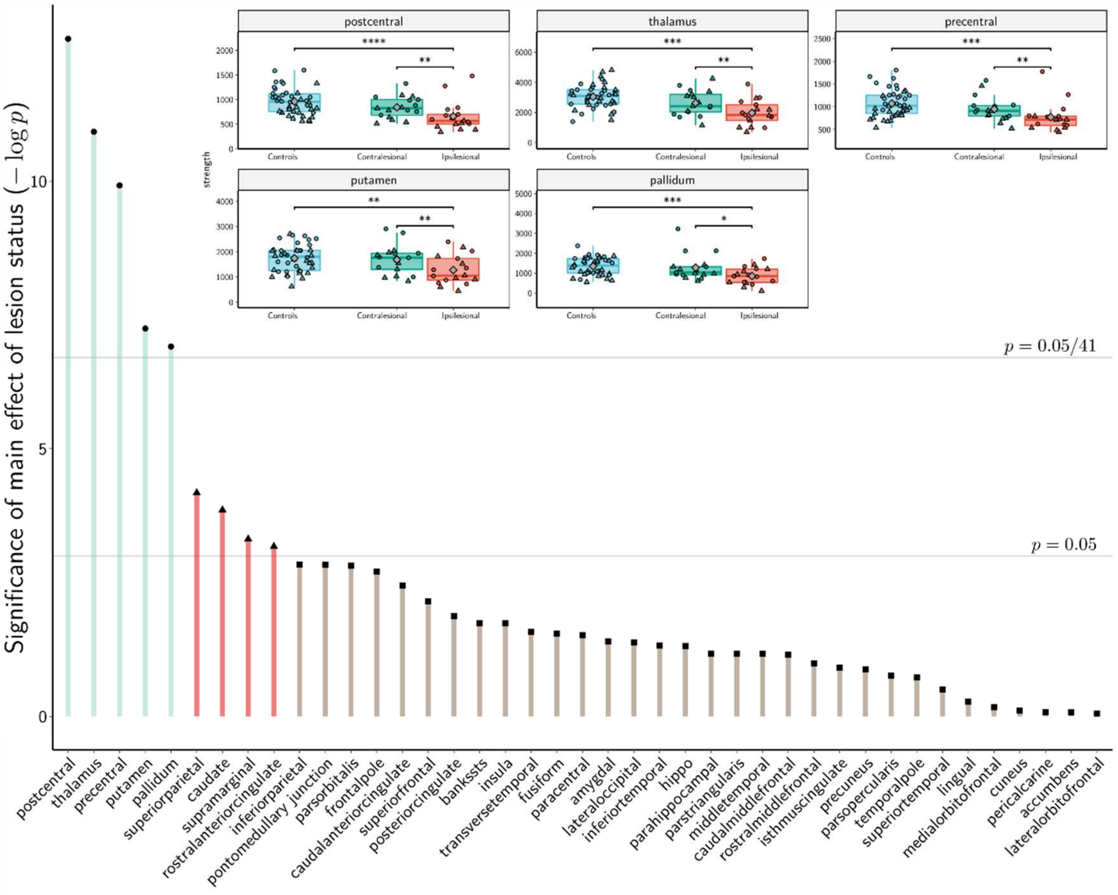
Significance of main effect of lesion status ($-\log p$)
$p = 0.05/41$
$p = 0.05$
postcentral
thalamus
precentral
putamen
pallidum
strength
Controls
Contralesional
Ipsilesional
postcentral
thalamus
precentral
putamen
pallidum
superiorparietal
caudate
supramarginal
rostralanteriorcingulate
inferiorparietal
pontomedullary junction
parsorbitalis
frontalpole
caudalanteriorcingulate
superiorfrontal
posteriorcingulate
bankssts
insula
transversetemporal
fusiform
paracentral
amygdal
lateraloccipital
inferiortemporal
hippo
parahippocampal
parstriangularis
middletemporal
caudalmiddlefrontal
rostralmiddlefrontal
isthmuscingulate
precuneus
parsopercularis
temporalpole
superiortemporal
lingual
medialorbitofrontal
cuneus
pericalcarine
accumbens
lateralorbitofrontal

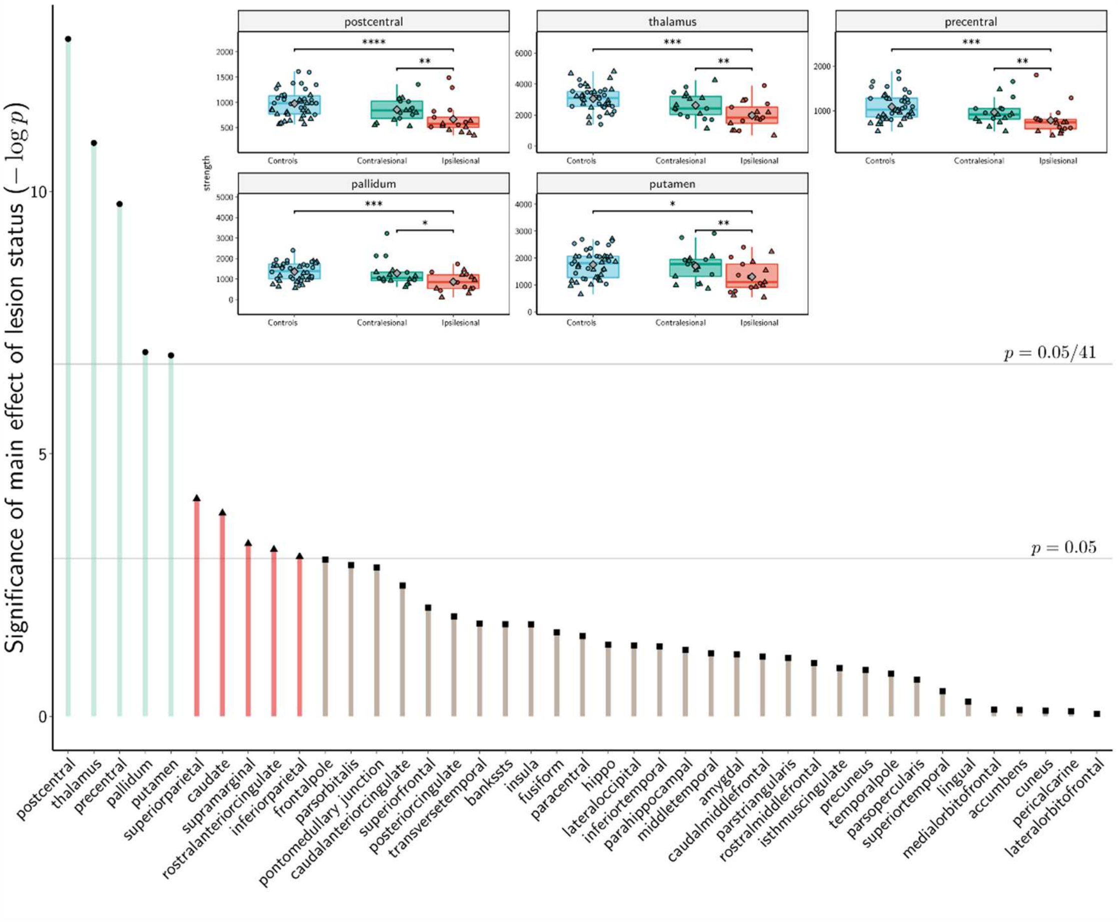
Significance of main effect of lesion status ($-\log p$)
$p = 0.05/41$
$p = 0.05$
postcentral
thalamus
precentral
pallidum
putamen
strength
Controls
Contralesional
Ipsilesional
postcentral
thalamus
precentral
pallidum
putamen
superiorparietal
caudate
supramarginal
rostralanteriorcingulate
inferiorparietal
frontalpole
parsorbitalis
pontomedullary junction
caudalanteriorcingulate
superiorfrontal
posteriorcingulate
transversetemporal
bankssts
insula
fusiform
paracentral
hippo
lateraloccipital
inferiortemporal
parahippocampal
middletemporal
amygdal
caudalmiddlefrontal
parstriangularis
rostralmiddlefrontal
isthmuscingulate
precuneus
temporalpole
parsopercularis
superiortemporal
lingual
medialorbitofrontal
accumbens
cuneus
pericalcarine
lateralorbitofrontal

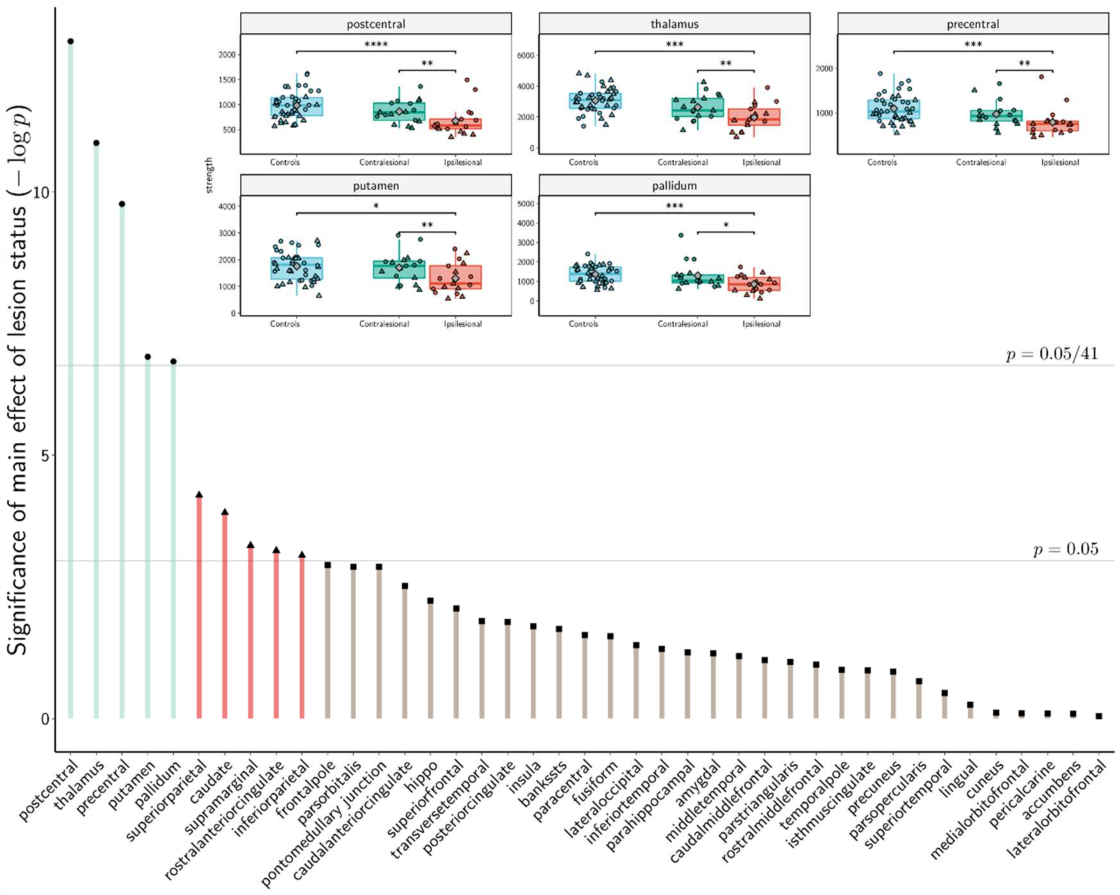
postcentral
thalamus
precentral
putamen
pallidum
strength
Controls
Contralesional
Ipsilesional
Significance of main effect of lesion status ($-\log p$)
$p = 0.05/41$
$p = 0.05$
postcentral
thalamus
precentral
putamen
pallidum
superiorparietal
caudate
supramarginal
rostralanteriorcingulate
inferiorparietal
frontalpole
parsorbitalis
pontomedullary junction
caudalanteriorcingulate
hippo
superiorfrontal
transversetemporal
posteriorcingulate
insula
bankssts
paracentral
fusiform
lateraloccipital
inferiortemporal
parahippocampal
amygdal
middletemporal
caudalmiddlefrontal
parstriangularis
rostralmiddlefrontal
temporalpole
isthmuscingulate
precuneus
parsopercularis
superiortemporal
lingual
cuneus
medialorbitofrontal
pericalcarine
accumbens
lateralorbitofrontal

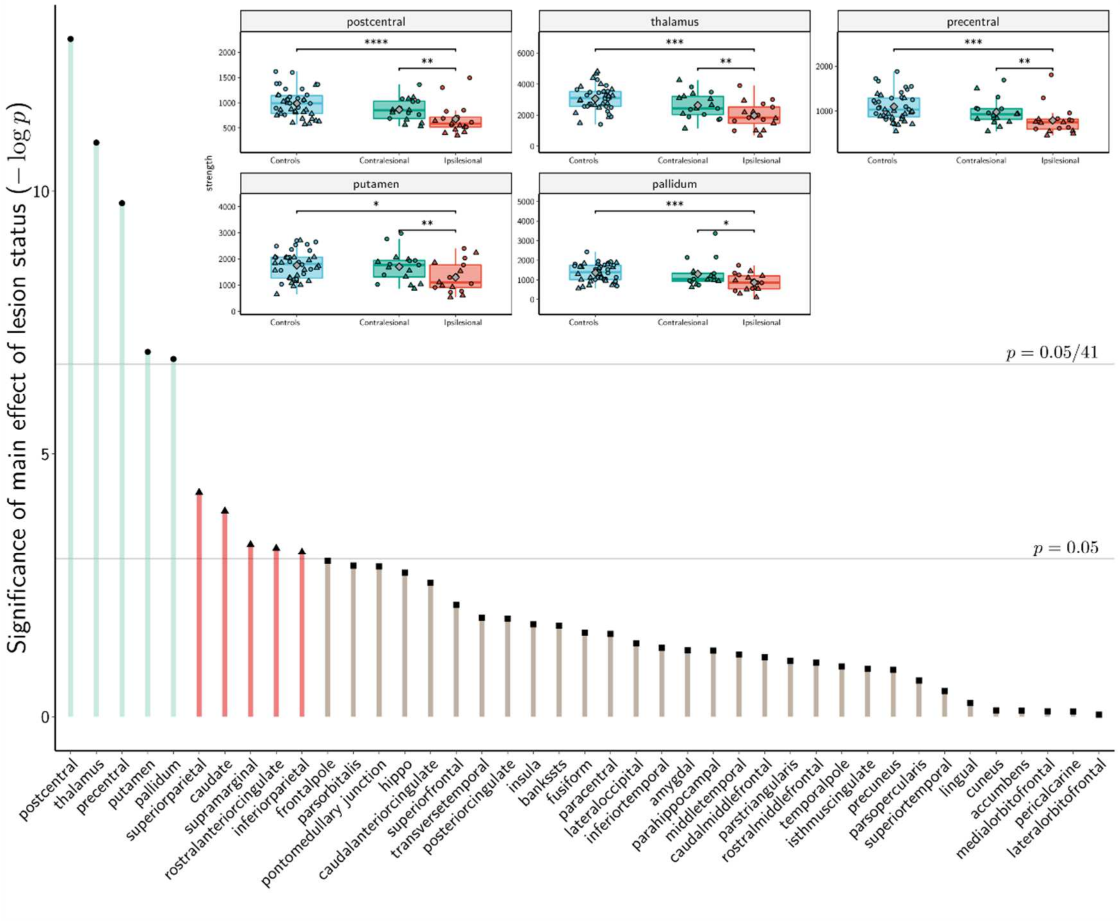
postcentral
thalamus
precentral
putamen
pallidum
strength
Controls
Contralesional
Ipsilesional
Significance of main effect of lesion status ($-\log p$)
$p = 0.05/41$
$p = 0.05$
postcentral
thalamus
precentral
putamen
pallidum
superiorparietal
caudate
supramarginal
rostralanteriorcingulate
inferiorparietal
frontalpole
parsorbitalis
pontomedullary junction
hippo
caudalanteriorcingulate
superiorfrontal
transversetemporal
posteriorcingulate
insula
bankssts
fusiform
paracentral
lateraloccipital
inferiortemporal
amygdal
parahippocampal
middletemporal
caudalmiddlefrontal
parstriangularis
rostralmiddlefrontal
temporalpole
isthmuscingulate
precuneus
parsopercularis
superiortemporal
lingual
cuneus
accumbens
medialorbitofrontal
pericalcarine
lateralorbitofrontal

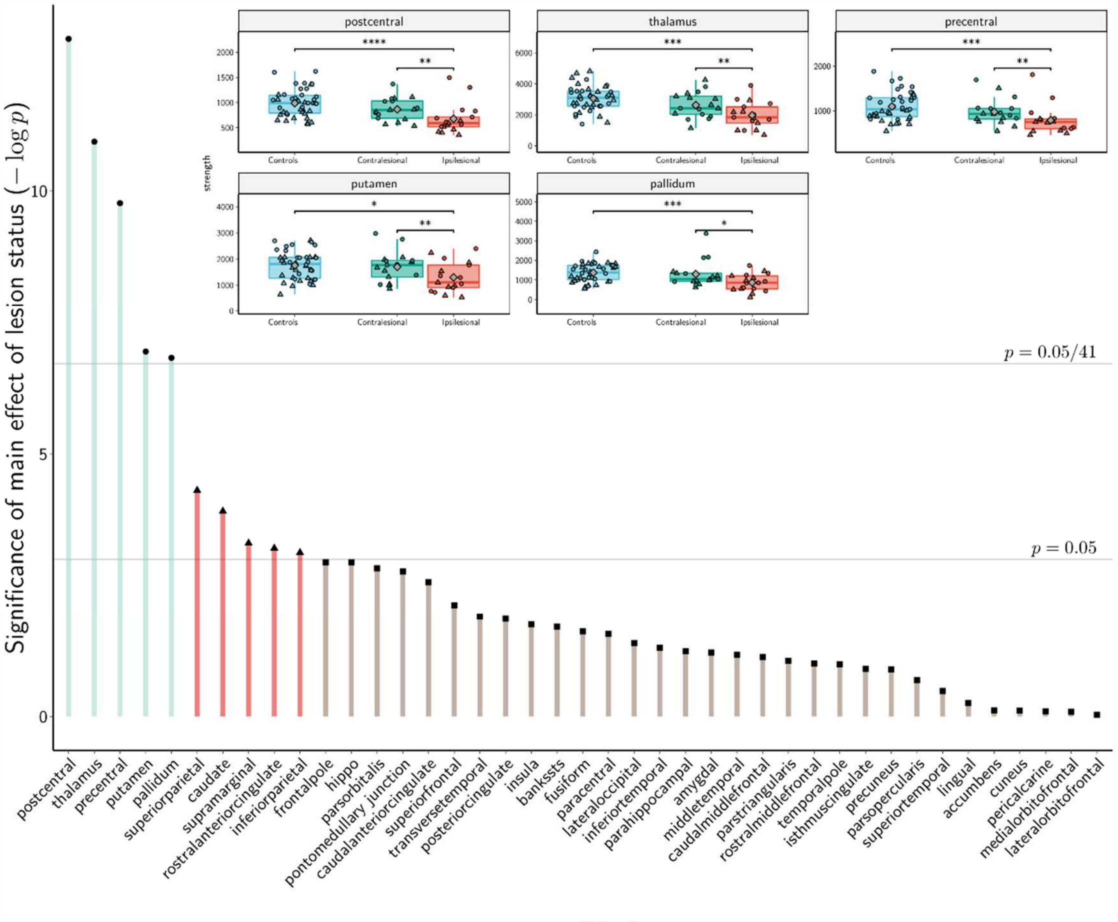

Supplemental figure S1: Sensitivity analysis for the effect of network density on differences in local network architecture. Individual panels correspond to network densities imposed by proportional thresholding ranging from 20% (upper most illustration) to 80% (last illustration), increasing in 10% increments. Height of bars indicate strength of statistical evidence from linear mixed effects modelling for main effect of lesion status (ipsilesional, contralesional, healthy control) on local node strength of each predefined region (negative logarithm of the obtained p-value). Horizontal lines correspond to significant effect in mass univariate modelling (p=0.05 and p=0.05/41 as the criticality threshold after Bonferroni correction). Inset: Node strength of a consistent set of five brain regions exhibiting a significant effect of lesions status after Bonferroni correction at each threshold. Diamonds mark the group means. Individual circles and triangles indicate left and right hemispheres, respectively. Individual contrasts with a statistical significance are indicated by brackets. * p<0.05; ** p<0.005; *** p<0.0005.

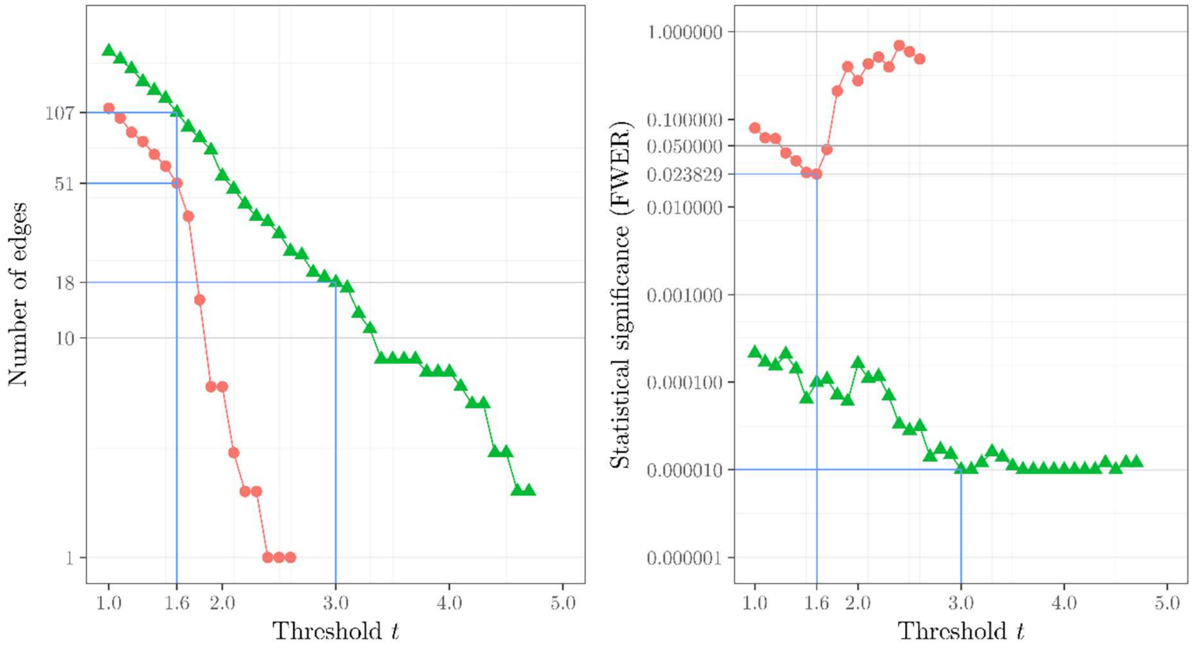

Supplemental figure S2: Statistical detail for network based statistics (NBS)`(Zalesky *et al.*, 2010)**.** Number of edges (left panel) and family-wise statistical significance p (right panel) of the subnetworks consisting of supra-critical edges whose strength in ipsilesional (green triangles) and contralesional (red dots) hemispheres is sufficiently different from that in healthy controls. Supracriticality of an edge is defined by the t-statistic in a univariate linear-mixed effects model exceeding the threshold t (horizontal axis). The p-value is computed from a Monte–Carlo estimation of the largest connected component in the supracritical networks obtained from n = 10^7 random permutations of the original data subject to preserving the anatomical left/right matching.

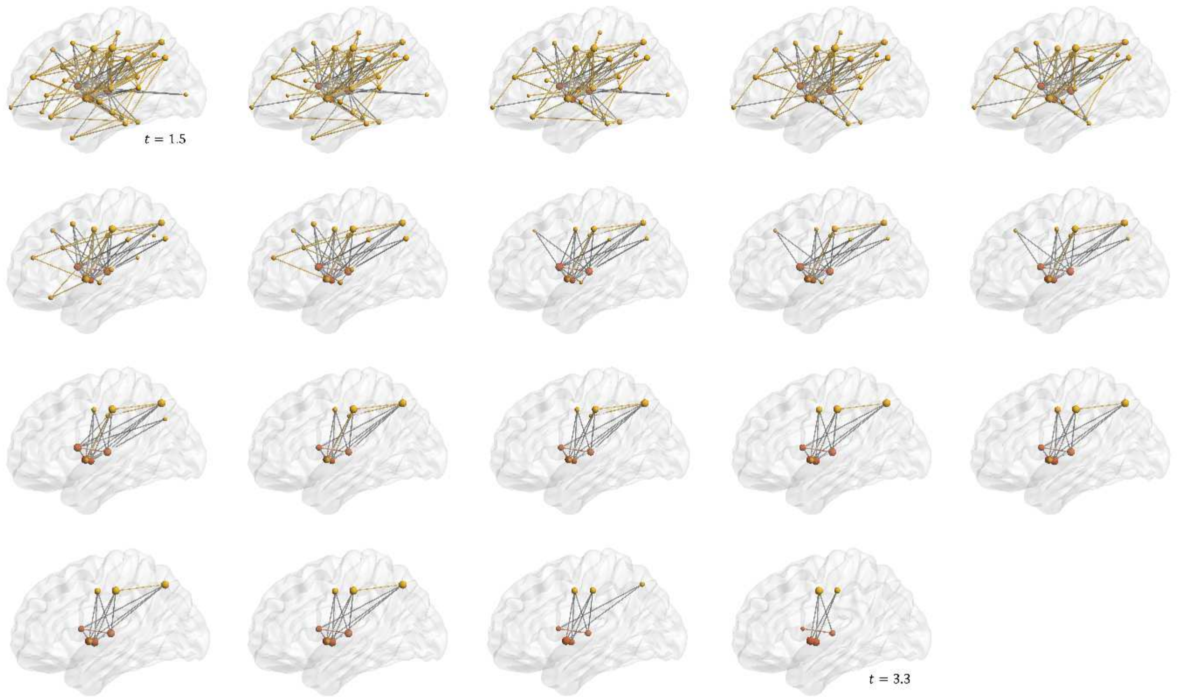

Supplemental figure S3: Network lesion mapping in stroke hemispheres. Subnetworks detected by NBS corresponding with increasing t-values (starting at t=1.5 up to t=3.3, higher values correspond to more conservative thresholds)

Review Only

Supplemental References

Desikan RS, Ségonne F, Fischl B, Quinn BT, Dickerson BC, Blacker D, et al. An automated labeling system for subdividing the human cerebral cortex on MRI scans into gyral based regions of interest. Neuroimage 2006; 31: 968–80.
Drakesmith M, Caeyenberghs K, Dutt a., Lewis G, David a. SS, Jones DKK. Overcoming the effects of false positives and threshold bias in graph theoretical analyses of neuroimaging data. Neuroimage 2015; 118: 313–333.
Satterthwaite FE. An Approximate Distribution of Estimates of Variance Components. Biometrics Bull. 1946
Zalesky A, Fornito A, Bullmore ET. Network-based statistic: identifying differences in brain networks. Neuroimage 2010; 53: 1197–207.

For Review Only
