# Supplementary material for: Altered topology of large-scale structural brain networks in chronic stroke
Source: Brain Commun. 2019 Oct 4;1(1):fcz020. doi: 10.1093/braincomms/fcz020 (PMC7425306; doi:10.1093/braincomms/fcz020)
Supplement: fcz020_Supplementary_Data [file fcz020_supplementary_data.zip › Supplementary_material.pdf]

Altered topology of structural brain networks in chronic stroke

Supplementary Material

Bastian Cheng*[1], Eckhard Schlemm*[1], Robert Schulz[1], Marlene Boenstrup[1,2], Arnaud Messé[3], Claus Hilgetag3, Christian Gerloff[1], Götz Thomalla[1]

[1] Department of Neurology, University Medical Center Hamburg-Eppendorf, Hamburg, Germany

[2] Human Cortical Physiology and Neurorehabilitation Section, National Institute of Neurological Disorders and Stroke, National Institutes of Health, Bethesda, MD, USA

[3] Institute of Computational Neuroscience, University Medical Center Hamburg-Eppendorf, Hamburg, Germany

[*] Both authors contributed equally

Correspondence:

Bastian Cheng, MD
University Medical Center Hamburg Eppendorf
Martinistraße 52
D-20246 Hamburg
fon: 0049-40-7410-51082
fax: 0049-40--7410-57391
E-Mail: b.cheng@uke.de

Supplemental methods

Network-based statistics (NBS)

Network lesion mapping with the networks-based statistics toolbox requires one to specify a threshold.(Zalesky *et al.*, 2010) The optimal choice of threshold is not *a priori* clear. We therefore examined the resulting network disruptions for a range of thresholds and plotted their size (number of edges) as well as statistical significance (family-wise error rate) in supplementary Fig. S2. As expected, the sizes of NBS networks decrease with more stringent thresholds and are generally larger and more significant in ipsilesional hemispheres than in contralesional hemispheres. We also note that FEWR of contralesional NBS networks is minimised at $t=1.6$ which corresponds, roughly, to an edge-wise significance level of $\alpha=0.05$, as determined in a separate simulation study (not shown). Network disruptions in hemispheres directly affected by stroke persist at much less liberal thresholds, with a region of relative stability around $t=3.0$. In supplementary Fig. S3, we show balls-and-stick models of the ipsilesional network disruptions identified around that stricter threshold, allowing an assessment of the sensitivity towards changes in the threshold and isolation of the stable core.

Abbreviation	Full anatomical description
accumbens	Nucleus accumbens
amygdal	Amygdala
bankssts	Banks superior temporal sulcus
caudalanteriorcingulate	Caudal anterior-cingulate cortex
caudalmiddlefrontal	Caudal middle frontal gyrus
caudate	Caudate nucleus
cuneus	Cuneus cortex
frontalpole	Frontal pole
fusiform	Fusiform gyrus
hippo	Hippocampus
inferiorparietal	Inferior parietal cortex
inferiortemporal	Inferior temporal gyrus
insula	Insula
isthmuscingulate	Isthmus–cingulate cortex
lateraloccipital	Lateral occipital cortex
lateralorbitofrontal	Lateral orbital frontal cortex
lingual	Lingual gyrus
medialorbitofrontal	Medial orbital frontal cortex
middletemporal	Middle temporal gyrus
pallidum	Globus pallidum
paracentral	Paracentral lobule
parahippocampal	Parahippocampal gyrus
parsopercularis	Pars opercularis
parsorbitalis	Pars orbitalis
parstriangularis	Pars triangularis
pericalcarine	Pericalcarine cortex
pontomedullary junction	Pontomedullary junction / Corticospinal tract
postcentral	Postcentral gyrus
posteriorcingulate	Posterior-cingulate cortex
precentral	Precentral gyrus
precuneus	Precuneus cortex
putamen	Putamen
rostralanteriorcingulate	Rostral anterior cingulate cortex
rostralmiddlefrontal	Rostral middle frontal gyrus
superiorfrontal	Superior frontal gyrus
superiorparietal	Superior parietal cortex
superiortemporal	Superior temporal gyrus
supramarginal	Supramarginal gyrus
temporalpole	Temporal pole
thalamus	Thalamus
transversetemporal	Transverse temporal cortex

Supplemental table S1: Cortical parcellation scheme applied for connectome generation according to the standardized FreeSurfer algorithm (Desikan *et al.*, 2006).

ID	Stroke hemisphere	Age	Gender	Time since stroke [days]	Grip force affected hand [kg]	UEFM	q50 ipsilesional	q50 contralesional
1	right	58	female	610	25	64	0.950	1.863
2	left	74	male	995	18	46	1.237	1.678
3	right	52	male	2387	13.5	37	1.479	1.921
4	left	57	male	587	39	60	1.458	2.217
5	right	71	male	803	30	49	1.172	1.491
6	left	63	male	367	33.4	66	0.906	1.139
7	left	66	male	361	33.5	66	0.750	1.256
8	right	69	male	350	47.67	66	0.754	1.119
9	right	71	male	348	37.3	66	1.053	1.281
10	right	74	female	363	25.67	66	0.429	1.145
11	left	52	male	563	42.3	66	0.426	2.245
12	right	71	male	377	34.67	66	1.238	1.154
13	right	53	male	340	44.3	66	0.980	1.463
14	right	64	male	402	18.33	60	1.206	1.249
15	left	59	female	1323	34.33	59	1.448	1.341
16	left	64	male	374	38.67	66	1.171	1.022
17	left	79	female	373	23.3	66	1.181	1.434

Supplemental table S2: Individual demographic, clinical and graph theoretical data from stroke patients included in the study. Mean connectivity strength (q50) is shown for the ipsi- and contralesional hemisphere. Abbreviations: UEFM, Fugl-Meyer assessment of the upper extremity.

	Lesion status			Test statistic
	Controls	Contralesional	Ipsilesional	
**Whole Brain**	1.56 ± 0.08^{a}	1.47 ± 0.09^{a}	1.05 ± 0.08	$F_{2,35.43} = 9.33$, $p = 0.001$
**Left Hemisphere**	1.59 ± 0.11^{a}	1.41 ± 0.10^{a}	1.07 ± 0.12	$F_{2,33} = 3.81$, $p = 0.0324$
**Right Hemisphere**	1.52 ± 0.11^{a}	1.54 ± 0.16^{a}	1.03 ± 0.10	$F_{2,33} = 4.42$, $p = 0.019$

Supplemental table S3: Global connectivity measures corrected for covariates age and gender. Reported are median connectivity strength (q_{50}) in left and right hemispheres affected by stroke, non-affected hemispheres in stroke patients, and hemispheres from healthy controls. The test statistics in the last column represent the effect of lesion status. In the pooled analysis of left and right hemispheres in the first row, the Satterthwaite approximation is used.(Satterthwaite, 1946) Identical superscripts within a row indicate the absence of a statistically significant difference in post-hoc mean-separation testing.

		Grip strength	UEFM
**Lesion volume [ml]**		r = 0.4428<br>p = 0.0751	r = 0.1228<br>p = 0.6387
**Median connectivity**		r = −0.3376<br>p = 0.1851	r = −0.5452<br>p = 0.0236
**GGP**	Normalised efficiency	r = 0.1495<br>p = 0.5668	r = −0.1721<br>p = 0.509
	Normalised Clustering	r = −0.0760<br>p = 0.7718	r = −0.1285<br>p = 0.5696
	Modularity	r = −0.1210<br>p = 0.6436	r = 0.1672<br>p = 0.5327
**Nodal strength**	Precentral	r = −0.3474<br>p = 0.1718	r = −0.3043<br>p = 0.2350
	Postcentral	r = −0.2433<br>p = 0.3468	r = −0.3109<br>p = 0.2245
	Putamen	r = −0.1204<br>p = 0.6452	r = −0.3928<br>p = 0.1188
	Pallidum	r = −0.3940<br>p = 0.1176	r = −0.3260<br>p = 0.2017
	Thalamus	r = 0.0695<br>p = 0.7909	r = 0.1365<br>p = 0.6013

Supplemental table S4: associations of imaging and graph theoretical measurements with clinical data. Values for lesion volume, median connectivity strength, global graph parameters (GGP) and local node strength with significant effects of lesion status (figure 4, main manuscript) are shown. Linear correlations r between structural properties of ipsilesional stroke hemispheres and clinical characteristics. p−values are uncorrected. Abbreviations: GGP, global graph parameters; UEFM, upper extremities Fugl-Meyer Assessment of Motor Recovery.

	Normalized Efficiency				Normalized Clustering				Modularity			
	k*	ES	A	A_{crit}	k*	ES	A	A_{crit}	k*	ES	A	A_{crit}
**Main effect**	0.4	3.8015	13.29	3.26	0.4	4.7397	10.27	3.05	-	-	-	-
**Simple effects**												
**-Ipsilesional vs control**	0.3	-2.8390	33.47	1.13	0.4	4.8973	80.06	2.97	0.5	2.7231	39.36	5.30
**-Contralesional vs control**	0.4	-2.0216	11.24	3.08	0.2	2.3737	11.15	2.93	-	-	-	-
**-Ipsi- vs contralesional**	-	-	-	-	0.2	3.1258	27.04	2.31	0.5	2.1624	16.52	2.52

Supplemental table S5: Statistics from MTCP- Analysis (Drakesmith *et al.*, 2015)**.** Statistical details of a multi-threshold permutation correction analysis of the effect of lesion status (healthy, contralesional, ipsilesional) on intrahemispheric global graph parameters. κ^* denotes the network density with the maximal individual effect size (ES), which in turn is quantified by the value of the F and t statistic for the omnibus test and marginal contrasts, respectively. A denotes the empirical supra-critical area under curve (sc-AUC), whereas A_{crit} is the critical value of sc-AUC at a significance level of $\alpha = 0.05$ obtained from $n = 10^4$ random permutations of the empirical data.

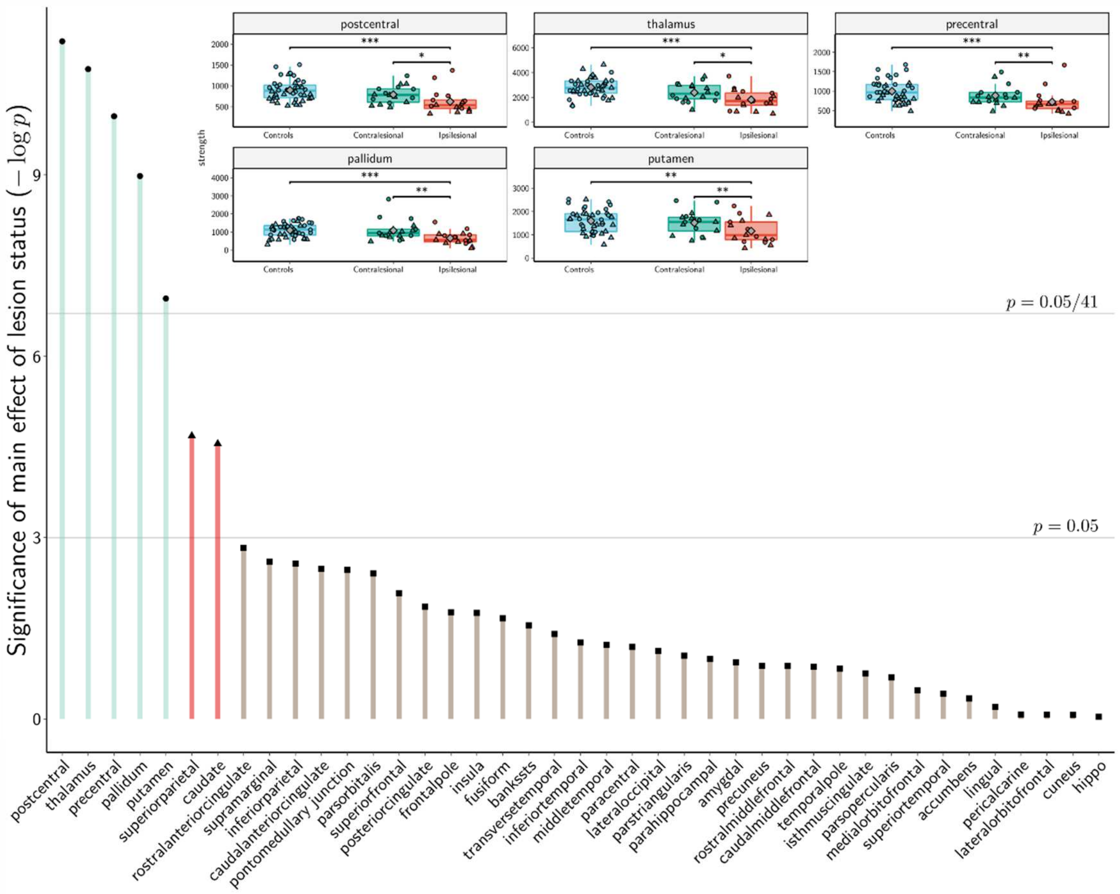

Significance of main effect of lesion status $(-\log p)$
$p = 0.05/41$
$p = 0.05$
postcentral
thalamus
precentral
pallidum
putamen
strength
Controls
Contralesional
Ipsilesional
postcentral
thalamus
precentral
pallidum
putamen
superiorparietal
caudate
rostralanteriorcingulate
supramarginal
inferiorparietal
caudalanteriorcingulate
pontomedullary junction
parsorbitalis
superiorfrontal
posteriorcingulate
frontalpole
insula
fusiform
bankssts
transversetemporal
inferiortemporal
middletemporal
paracentral
lateraloccipital
parstriangularis
parahippocampal
amygdal
precuneus
rostralmiddlefrontal
caudalmiddlefrontal
temporalpole
isthmuscingulate
parsopercularis
medialorbitofrontal
superiortemporal
accumbens
lingual
pericalcarine
lateralorbitofrontal
cuneus
hippo

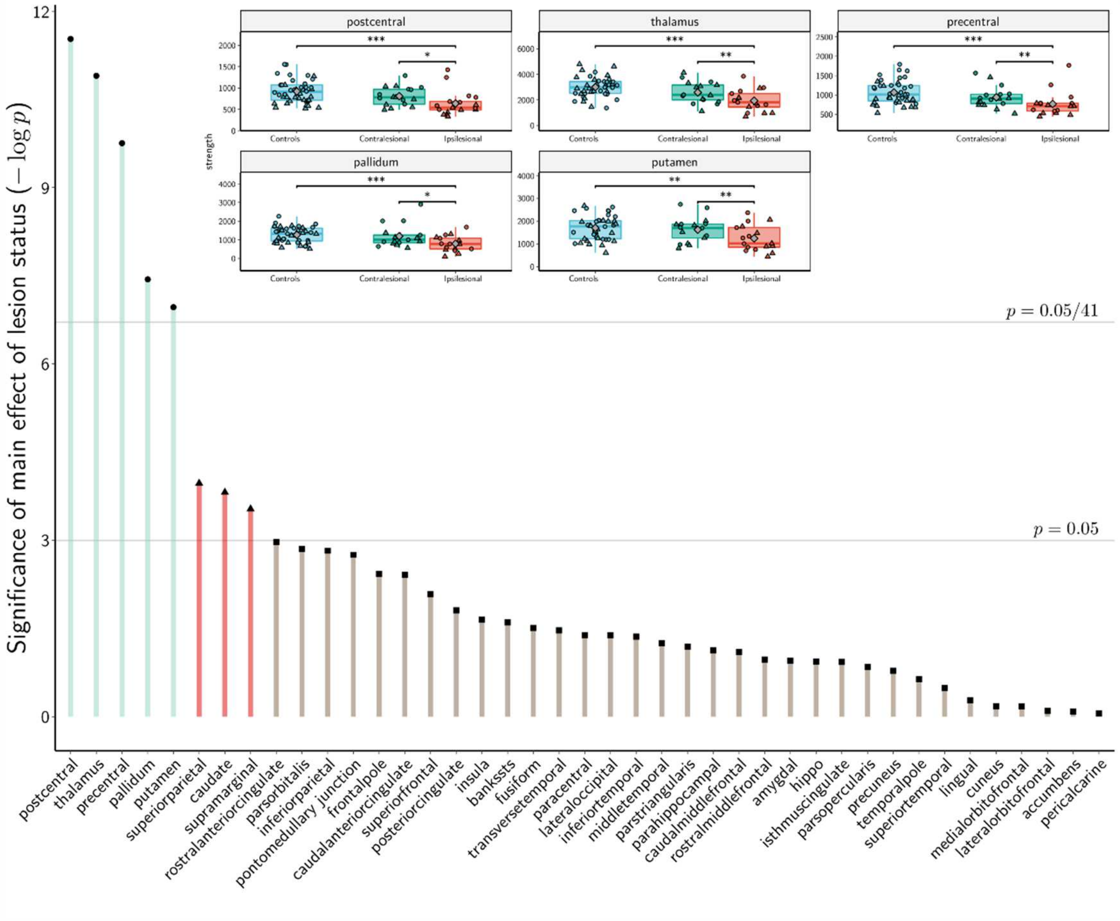

Significance of main effect of lesion status $(-\log p)$
$p = 0.05/41$
$p = 0.05$
postcentral
thalamus
precentral
pallidum
putamen
strength
Controls
Contralesional
Ipsilesional
postcentral
thalamus
precentral
pallidum
putamen
superiorparietal
caudate
supramarginal
rostralanteriorcingulate
parsorbitalis
inferiorparietal
pontomedullary junction
frontalpole
caudalanteriorcingulate
superiorfrontal
posteriorcingulate
insula
bankssts
fusiform
transversetemporal
paracentral
lateraloccipital
inferiortemporal
middletemporal
parstriangularis
parahippocampal
caudalmiddlefrontal
rostralmiddlefrontal
amygdal
hippo
isthmuscingulate
parsopercularis
precuneus
temporalpole
superiortemporal
lingual
cuneus
medialorbitofrontal
lateralorbitofrontal
accumbens
pericalcarine

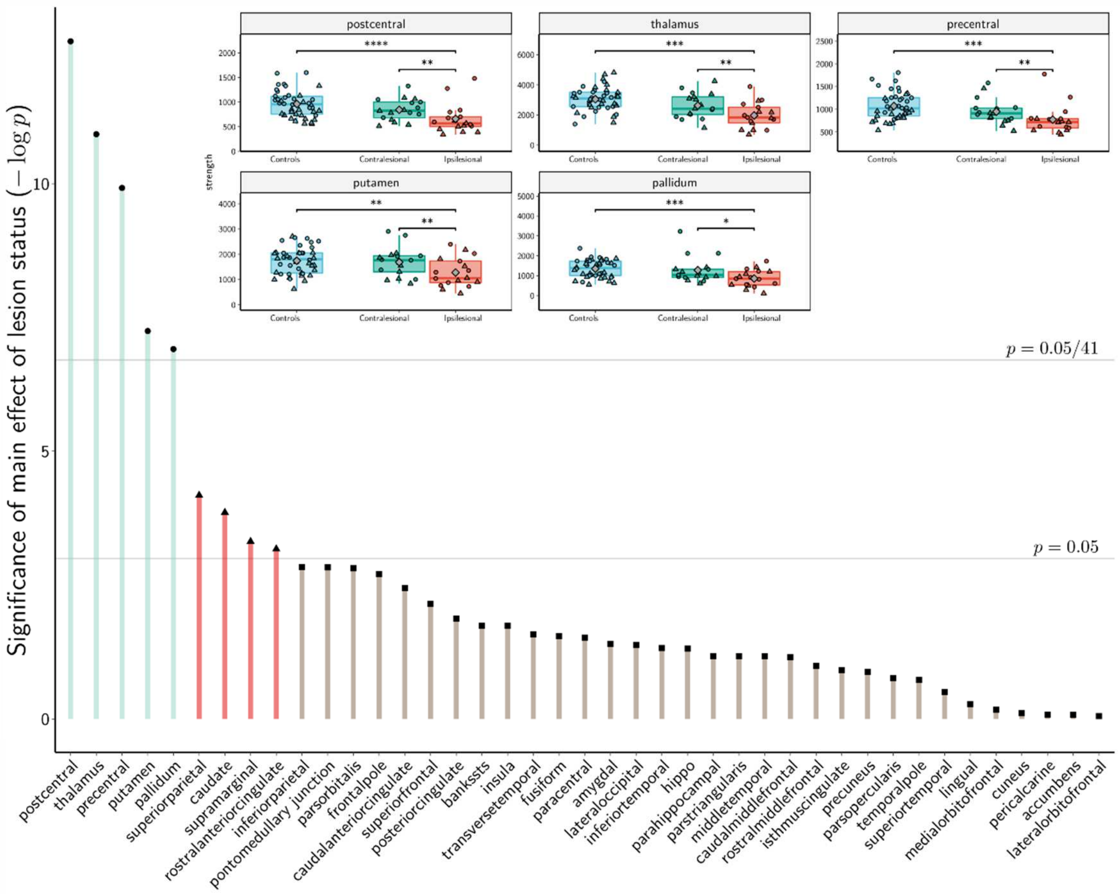
Significance of main effect of lesion status $(-\log p)$
$p = 0.05/41$
$p = 0.05$
postcentral
thalamus
precentral
putamen
pallidum
strength
Controls
Contralesional
Ipsilesional
postcentral thalamus precentral putamen pallidum superiorparietal caudate supramarginal rostralanteriorcingulate inferiorparietal pontomedullary junction parsorbitalis frontalpole caudalanteriorcingulate superiorfrontal posteriorcingulate bankssts insula transversetemporal fusiform paracentral amygdal lateraloccipital inferiortemporal hippo parahippocampal parstriangularis middletemporal caudalmiddlefrontal rostralmiddlefrontal isthmuscingulate precuneus parsopercularis temporalpole superiortemporal lingual medialorbitofrontal cuneus pericalcarine accumbens lateralorbitofrontal

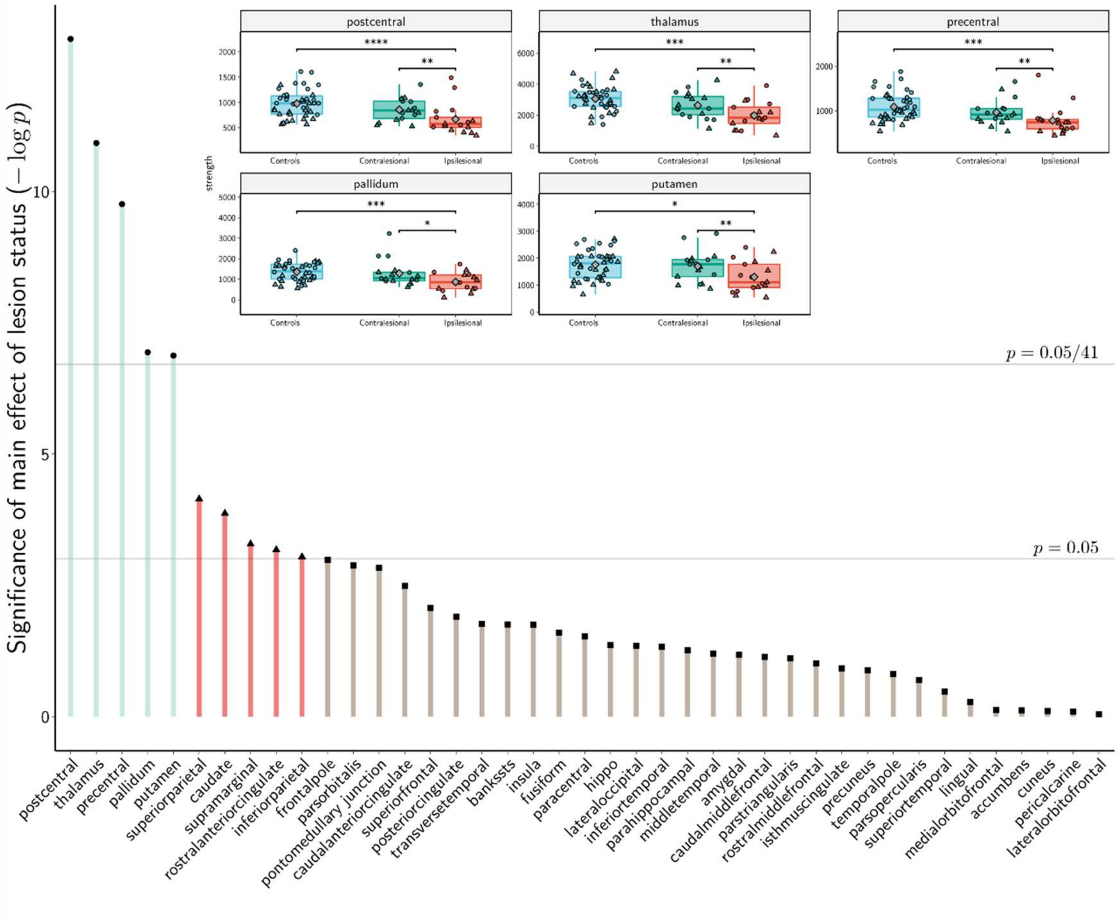
Significance of main effect of lesion status $(-\log p)$
$p = 0.05/41$
$p = 0.05$
postcentral
thalamus
precentral
pallidum
putamen
strength
Controls
Contralesional
Ipsilesional
postcentral thalamus precentral pallidum putamen superiorparietal caudate supramarginal rostralanteriorcingulate inferiorparietal frontalpole parsorbitalis pontomedullary junction caudalanteriorcingulate superiorfrontal posteriorcingulate transversetemporal bankssts insula fusiform paracentral hippo lateraloccipital inferiortemporal parahippocampal middletemporal amygdal caudalmiddlefrontal parstriangularis rostralmiddlefrontal isthmuscingulate precuneus temporalpole parsopercularis superiortemporal lingual medialorbitofrontal accumbens cuneus pericalcarine lateralorbitofrontal

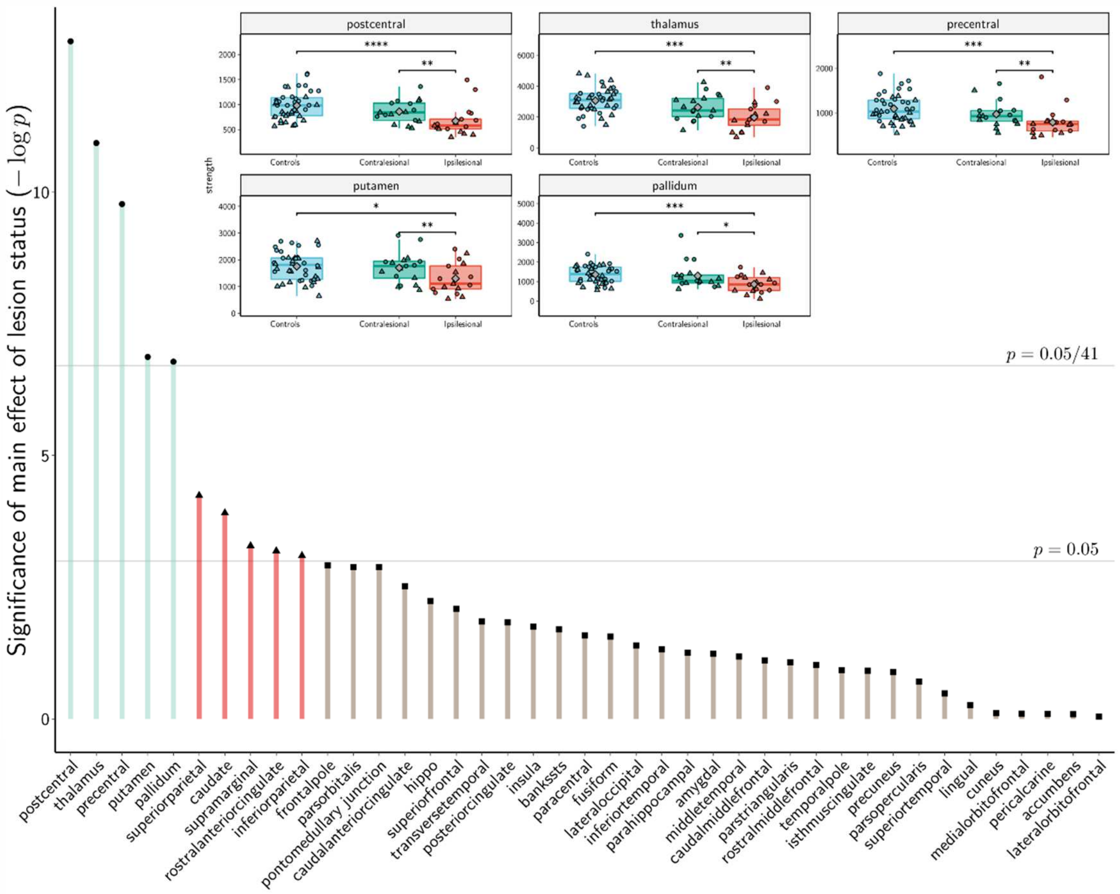
Significance of main effect of lesion status $(-\log p)$
0
5
10
$p = 0.05/41$
$p = 0.05$
postcentral
thalamus
precentral
putamen
pallidum
strength
Controls
Contralesional
Ipsilesional
postcentral
thalamus
precentral
putamen
pallidum
superiorparietal
caudate
supramarginal
rostralanteriorcingulate
inferiorparietal
frontalpole
parsorbitalis
pontomedullary junction
caudalanteriorcingulate
hippo
superiorfrontal
transversetemporal
posteriorcingulate
insula
bankssts
paracentral
fusiform
lateraloccipital
inferiortemporal
parahippocampal
amygdal
middletemporal
caudalmiddlefrontal
parstriangularis
rostralmiddlefrontal
temporalpole
isthmuscingulate
precuneus
parsopercularis
superiortemporal
lingual
cuneus
medialorbitofrontal
pericalcarine
accumbens
lateralorbitofrontal

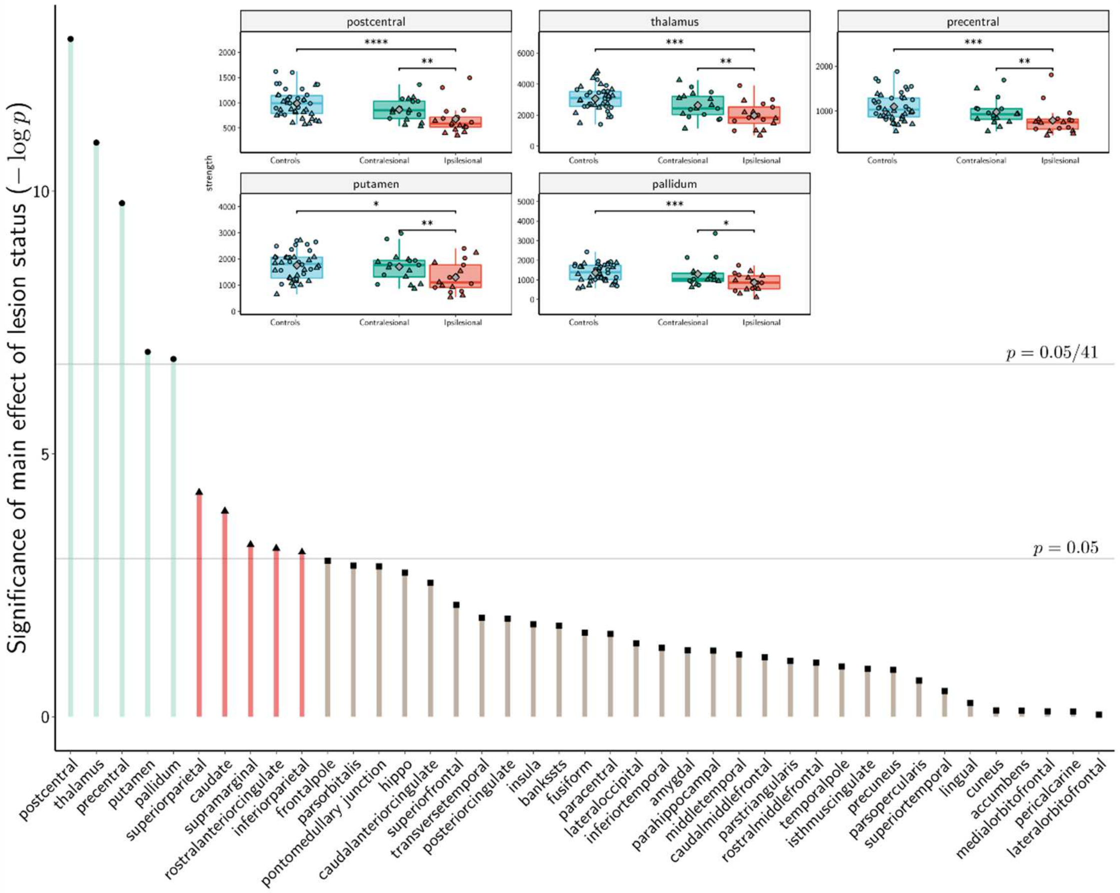
Significance of main effect of lesion status $(-\log p)$
0
5
10
$p = 0.05/41$
$p = 0.05$
postcentral
thalamus
precentral
putamen
pallidum
strength
Controls
Contralesional
Ipsilesional
postcentral
thalamus
precentral
putamen
pallidum
superiorparietal
caudate
supramarginal
rostralanteriorcingulate
inferiorparietal
frontalpole
parsorbitalis
pontomedullary junction
hippo
caudalanteriorcingulate
superiorfrontal
transversetemporal
posteriorcingulate
insula
bankssts
fusiform
paracentral
lateraloccipital
inferiortemporal
amygdal
parahippocampal
middletemporal
caudalmiddlefrontal
parstriangularis
rostralmiddlefrontal
temporalpole
isthmuscingulate
precuneus
parsopercularis
superiortemporal
lingual
cuneus
accumbens
medialorbitofrontal
pericalcarine
lateralorbitofrontal

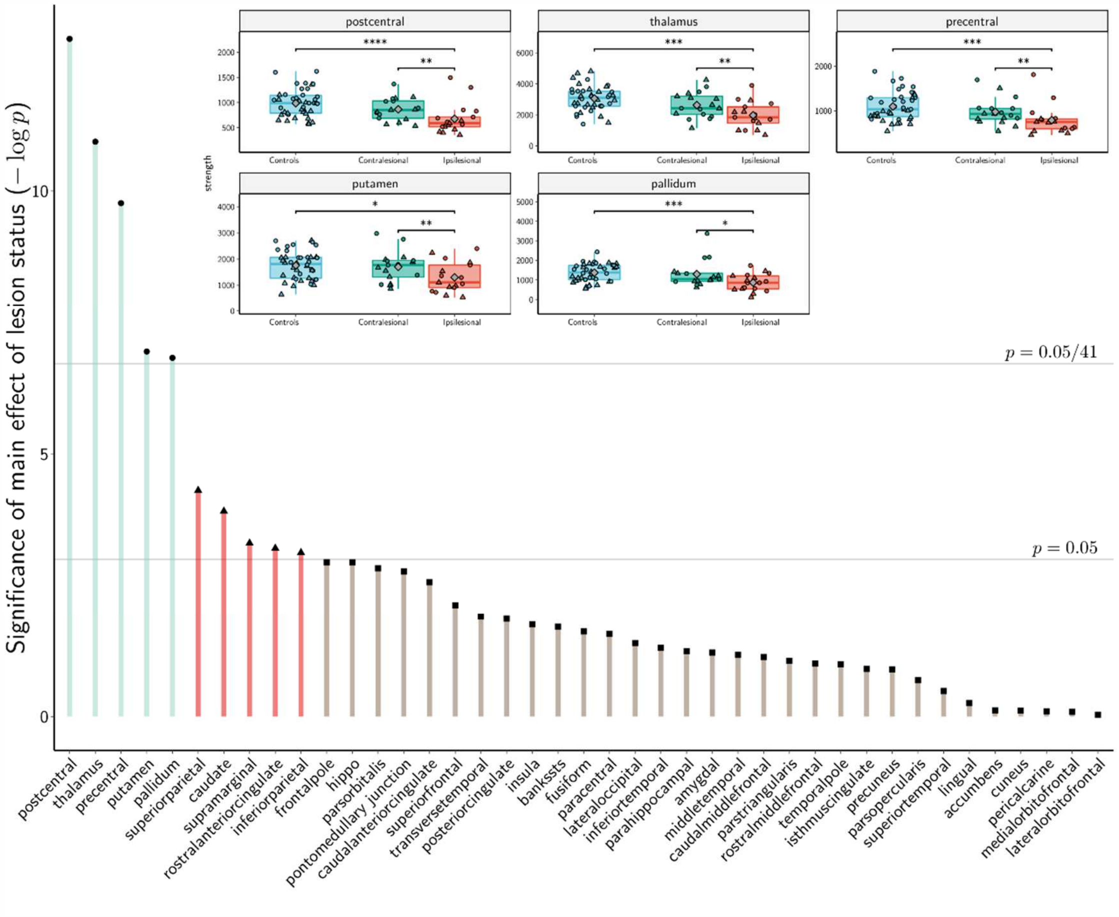

Supplemental figure S1: Sensitivity analysis for the effect of network density on differences in local network architecture. Individual panels correspond to network densities imposed by proportional thresholding ranging from 20% (upper most illustration) to 80% (last illustration), increasing in 10% increments. Height of bars indicate strength of statistical evidence from linear mixed effects modelling for main effect of lesion status (ipsilesional, contralesional, healthy control) on local node strength of each predefined region (negative logarithm of the obtained p-value). Horizontal lines correspond to significant effect in mass univariate modelling (p=0.05 and p=0.05/41 as the criticality threshold after Bonferroni correction). Inset: Node strength of a consistent set of five brain regions exhibiting a significant effect of lesions status after Bonferroni correction at each threshold. Diamonds mark the group means. Individual circles and triangles indicate left and right hemispheres, respectively. Individual contrasts with a statistical significance are indicated by brackets. * p<0.05; ** p<0.005; *** p<0.0005.

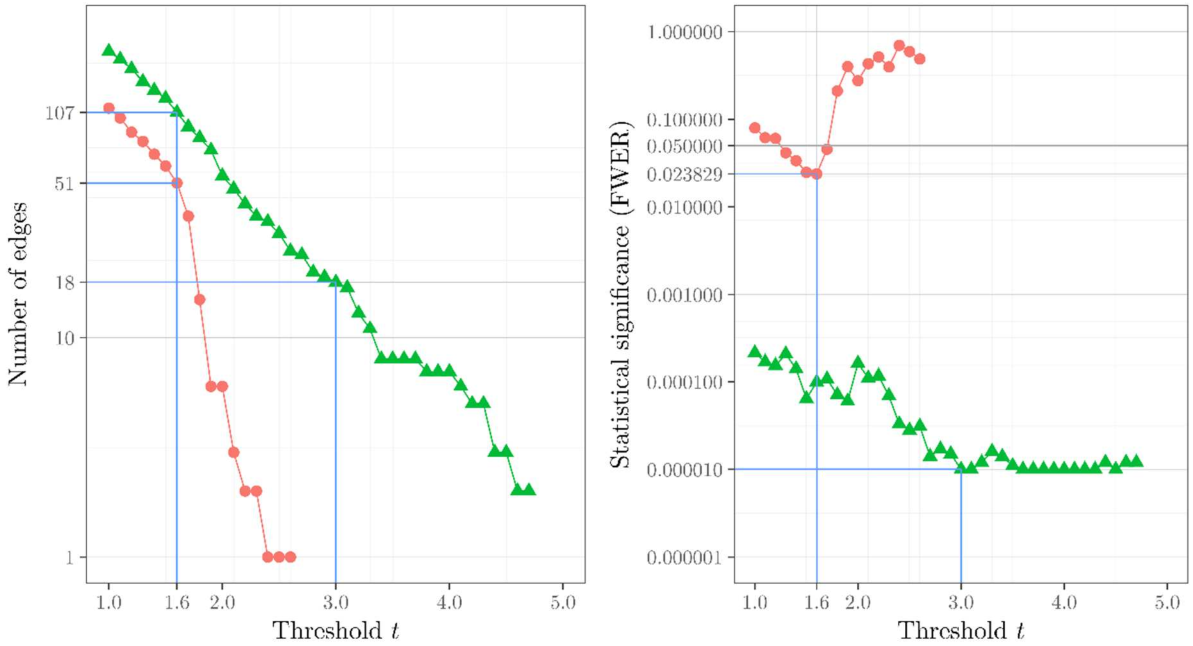

Supplemental figure S2: Statistical detail for network based statistics (NBS)` (Zalesky *et al.*, 2010)**.** Number of edges (left panel) and family-wise statistical significance p (right panel) of the subnetworks consisting of supra-critical edges whose strength in ipsilesional (green triangles) and contralesional (red dots) hemispheres is sufficiently different from that in healthy controls. Supracriticality of an edge is defined by the t-statistic in a univariate linear-mixed effects model exceeding the threshold t (horizontal axis). The p-value is computed from a Monte–Carlo estimation of the largest connected component in the supracritical networks obtained from $n = 10^7$ random permutations of the original data subject to preserving the anatomical left/right matching.

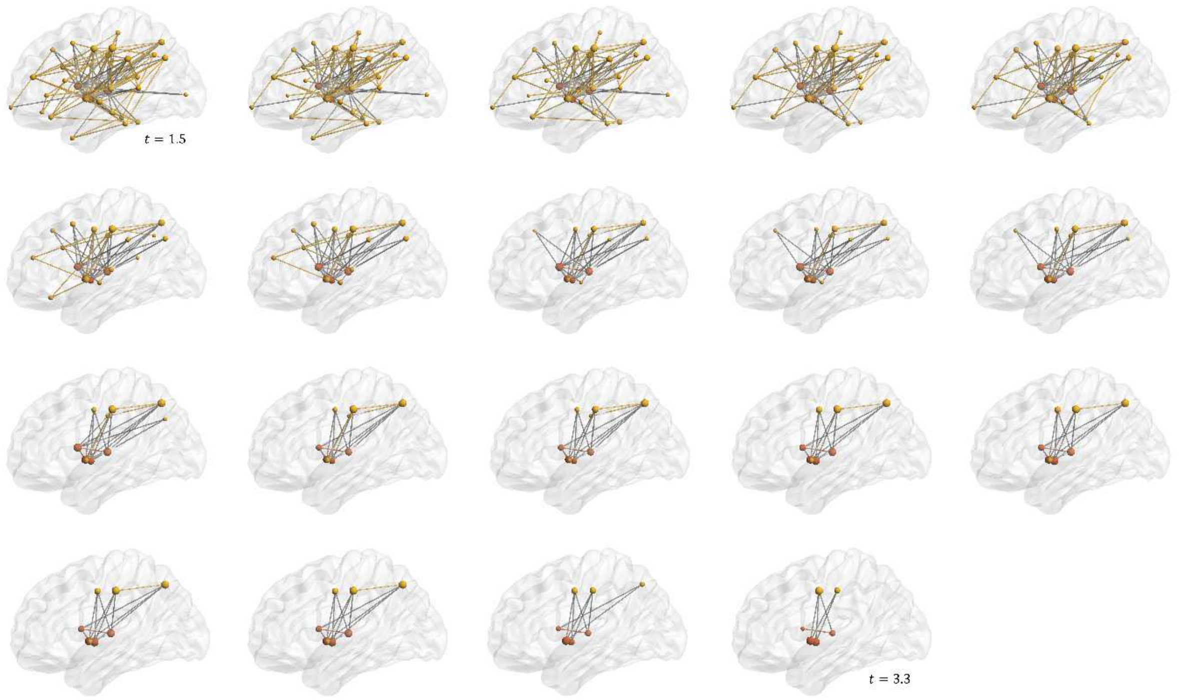

Supplemental figure S3: Network lesion mapping in stroke hemispheres. Subnetworks detected by NBS corresponding with increasing t-values (starting at t=1.5 up to t=3.3, higher values correspond to more conservative thresholds)

Supplemental References

Desikan RS, Ségonne F, Fischl B, Quinn BT, Dickerson BC, Blacker D, et al. An automated labeling system for subdividing the human cerebral cortex on MRI scans into gyral based regions of interest. Neuroimage 2006; 31: 968–80.

Drakesmith M, Caeyenberghs K, Dutt a., Lewis G, David a. SS, Jones DKK. Overcoming the effects of false positives and threshold bias in graph theoretical analyses of neuroimaging data. Neuroimage 2015; 118: 313–333.

Satterthwaite FE. An Approximate Distribution of Estimates of Variance Components. Biometrics Bull. 1946

Zalesky A, Fornito A, Bullmore ET. Network-based statistic: identifying differences in brain networks. Neuroimage 2010; 53: 1197–207.
