# Supplementary material for: Altered topology of large-scale structural brain networks in chronic stroke
Source: Brain Commun. 2019 Oct 4;1(1):fcz020. doi: 10.1093/braincomms/fcz020 (PMC7425306; doi:10.1093/braincomms/fcz020)
Supplement: fcz020_Supplementary_Data [file Response_to_reviewers_comments_original.pdf › Response_to_reviewers_comments_original.pdf]

Revised manuscript:

"Altered topology of large-scale structural brain networks in chronic stroke"

Response to the reviewers of *Brain Communications*

We thank the reviewers for their thoughtful comments and suggestions, which clearly helped us to improve the manuscript. Please find below our detailed response to all the criticism raised. All changes in the manuscript have been marked (yellow).

Reviewer: 1

This paper addresses a timely and interesting question, namely how structural network topology is affected by stroke. The authors use some standard approaches to investigate differences between stroke patients and controls in two small samples, while also performing some basic simulation work. Although they are to be commended for attempting to tackle this topic, several issues need to be addressed:

Q1: Was fiber tracking performed from a gray matter mask only? We know that this might be problematic, which is why a lot of studies use the gray/white matter rim as a seed region.

A: We agree with the reviewer on the importance of cortical seed mask location in DTI tractography. Indeed, we have taken measures to constrain tracking masks to the surface underlying the cortical gray matter to avoid spurious and implausible tracking caused by the non-uniform distribution of fiber orientations in gray matter. We have now clarified this methodologically adding a more detailed description of our approach:

For cortical regions, masks from automated parcellation by Freesurfer were refined to delineate the gray–white matter boundary underlying the cortical areas to increase anatomical accuracy for DTI fiber tracking. Therefore, surface maps of the boundary between grey and white matter were generated by FreeSurfer and applied to constrain parcellation masks to a ribbon directly underlying the cortical grey matter. All resulting masks were visually checked for plausibility and accuracy.

Q2: Were the stroke lesions masked out? Was visual inspection used to check whether lesions induced strange results due to problems with the tracking algorithm? These are essential things to take into account when using diffusion imaging around lesions that may induce severe artifacts.

A: We thank the reviewer for addressing this issue, which we neglected to report in our manuscript. We have now clarified how fiber tracking was constrained by masking stroke lesions:

Stroke lesions were manually delineated on T1-weighted anatomical MR images and visually checked for plausibility and accuracy after registration to individual maps of fractional anisotropy (FA). Stroke lesion masks were applied to exclud affected brain regions from probabilistic fiber tracking. In addition, pathways were disregarded if they entered a voxel with FA < 0.15. This value was chosen based on previous studies and experience (Cheng *et al.*, 2014; Finger *et al.*, 2016) showing this threshold consistently preventing erroneous tracking between neighboring gyri.

Q3: What is the rationale of using 70/80% proportional thresholds? These are very high, while recent studies show that the 'correct' density of a structural network lies around 20%, but is certainly not as high as 70/80%.

A: We thank the reviewer for raising the important question of network density and its implication for the present analysis. Despite recent advances, the choice of a ‚correct‘ threshold, especially in weighted networks, remains controversial (Civier *et al.*, 2019). In order to avoid the problem of prescribing an a-priori threshold we analyzed networks over thresholds ranging from 20% to 80%. In addition to a threshold-agnostic global group effect that we established using the recently published MTCP method, this approach served as an intrinsic sensitivity analysis with respect to the choice of threshold and showed that group effects are stable over a wide range of network densities. In the manuscript, we added the following clarifications:

Estimates of anatomical connection density depend on the resolution of the underlying cortical parcellation and fibre reconstruction method (Bassett and Bullmore, 2017), with values ranging from less than 30% in the binary case to 66% in a Macaque tract-tracing study (Markov *et al.*, 2013). The optimal choice of network density for the analysis of imaging-derived weighted structural connectomes therefore remains controversial (Civier *et al.*, 2019). As done previously (Seguin *et al.*, 2018; Buchanan *et al.*, 2019), weighted brain networks were therefore thresholded by multiplication by a sparsity mask corresponding to the 20%, 25%, (...), 75% and 80% strongest connections in the average network of healthy participants and analysed over this range of network densities.

Q4: The statistical approach would be very robust in a large sample, but does bring up the question of overfitting and power issues in the context of only 17 (heterogeneous) patients being included. Can the authors comment on this issue?

We appreciate the reviewer's bringing up the issue of statistical power and agree that overfitting is a potential concern in a small-sample study. To minimize the risk of false-positive signals in our analysis we restricted analysis to a small set of well-established network measures (q50, efficiency, clustering and modularity) and simple statistical tests. In the case of unavoidable parameter choices that might affect results, such as network density for global graph measures or t-test threshold in the NBS analysis, we performed transparent sensitivity analyses, adapted the statistical approach to address the multiple-testing problem, and reported effect sizes for different parameter choices. We included sample-size as a limitation of the study:

Given the sample size of 17 patients, it was necessary to restrict attention to a small number of well-established network measures in order to minimise the risk of overfitting. Results should be confirmed and extended to more subtle network properties in larger cohorts of stroke patients.

Q5: The authors use 'changes' to indicate what are in fact differences between patients and controls. Although I understand that the assumption here is that the network measured in patients reflects possible changes that have occurred as a result of stroke, this assumption should be explicitized. Also, I would recommend making clear in the discussion that it is also possible that differences in network topology between groups are (in part) due to premorbid differences, as the increasingly rich literature on vulnerability in functional/structural networks already indicate. In the context of this manuscript, it could be that people with already greater segregation of their structural network because of some preexistent variable are at greater risk for stroke. Furthermore, the lack of association with any clinical parameters also take away from the linear view that the authors present on the impact of stroke on a structural connectome.

A: We agree with the reviewer that this is an important aspect for the interpretation of our results, which we have addressed as a limitation in the discussion of our results:

Several limitations of our study have to be mentioned. Considering the cross sectional design of our study and lack of longitudinal data, we cannot conclude that the observed alterations in structural network integrity and topology are solely caused by the localized stroke lesion. Although the observed changes are pathophysiologically plausible and reproducible in a basic network simulation model, it is conceivable that they are influenced by other premorbid risk factors for cerebrovascular diseases such as hypertension or diabetes commonly present in stroke patients. Since we did not systematically record these potentially confounding factors, further studies applying a multivariate analysis and longitudinal design are needed to elucidate the significance of potential pre-morbid vulnerabilities of brain networks in stroke patients.

Q6: "No thresholding of structural connectomes was applied for the analysis of local network parameters." Why not? Why was this procedure used for global, but not local measures, which are obviously also impacted by the total number/weight of connections?

We thank the reviewer for this important question, which, in addition to raising a very valid methodological concern, with which we agree, points out an erroneous description of our thresholding procedure in the original manuscript. Indeed, a fixed proportional thresholding with target network density 50% was used for the analysis of local network parameters. This value was chosen to be consistent with both recent interval estimates of the human structural connectome and the peak intrahemispheric efficiency we observed in our data. We included the remark in the section of graph-theoretical analysis, as well as an additional figure Fig S1 in the Supplement, illustrating the stability of the effect of lesion status on nodal strength across thresholds.

A fixed proportional thresholding of 50% was applied to structural connectomes for the analysis of local network parameters. This value was chosen so as to be consistent with both recent interval estimates of the mammalian structural connectome (Bassett and Bullmore, 2017) and the peak intrahemispheric efficiency we observed in our data. A sensitivity analysis for group differences in local network architecture with respect to network density was performed.

Q7: Grammar throughout the manuscript should be critically evaluated. For instance, the second sentence of the abstract is already incorrect and thereby confusing: "stroke lesion have shown do induce alterations in functional brain connectivity and brain network topology associated with clinical severity and recovery". Another example in which the incorrect use of 'on' obscures what is meant by the authors is the second to last sentence of the abstract: "We propose that the observed network changes in our patients are best explained on the disruption of inter- and intrahemispheric, long white matter fiber tracts connecting distant brain regions." Should this be 'by'? Finally, the use of hyphens (e.g. in graph-theory, 3-T scanner, etc) should be decreased.

A: We thank the reviewer for the thorough revision. To ameliorate these issues, the manuscript has been critically revised by a native speaker for spelling and grammatical errors. Throughout the manuscript, unnecessary hyphens have been deleted.

Reviewer: 2

Cheng and colleagues have investigated alterations in the structural connectome in the chronic response to stroke in a cohort of 17 individuals who have had focal cerebral ischemia caused by MCA stroke in comparison with an age matched control group of 21 individuals. Alterations to the post stroke connectome have been studied previously with EEG and fMRI and in the present manuscript the authors have used diffusion-weighted imaging and probabilistic fiber tracking to study the impact of focal lesions on the structural connectome. The findings are novel and will be of interest to the stroke community interested in how a focal lesion can impact on long term motor and cognitive deficits. There are some issues that should be addressed.

Q1: The timepoints post stroke when the patients were scanned is not well defined, and are referred to only as the ‘chronic response’ greater than 6 months. The IQR suggests that there is a lot of variability in the time that the individuals were scanned. It would be useful to have a table (perhaps in supplemental data or an online resourse) where the exact timepoints for each patient was reported, together with grip strength, sex of patient, and some of the key connectome data ie. Connectivity strength. It could be that stratifying the data based on recovery time post stroke may reveal trends in the data which may reveal how the connectome alters over time after a stroke (although the study is likely underpowered for this)

A: We agree with the reviewer that a more detailed description would be helpful for the reader to comprehend the characteristics of clinical and basic connectome data of our group of stroke patients. We have added a table to the supplementary materials.

ID	Stroke hemisphere	Age	Gender	Time since stroke [days]	Grip force affected hand [kg]	UEFM	q50 ipsilesional	q50 contralesional
1	right	58	female	610	25	64	0.950	1.863
2	left	74	male	995	18	46	1.237	1.678
3	right	52	male	2387	13.5	37	1.479	1.921
4	left	57	male	587	39	60	1.458	2.217
5	right	71	male	803	30	49	1.172	1.491
6	left	63	male	367	33.4	66	0.906	1.139
7	left	66	male	361	33.5	66	0.750	1.256
8	right	69	male	350	47.67	66	0.754	1.119
9	right	71	male	348	37.3	66	1.053	1.281
10	right	74	female	363	25.67	66	0.429	1.145
11	left	52	male	563	42.3	66	0.426	2.245
12	right	71	male	377	34.67	66	1.238	1.154
13	right	53	male	340	44.3	66	0.980	1.463
14	right	64	male	402	18.33	60	1.206	1.249
15	left	59	female	1323	34.33	59	1.448	1.341
16	left	64	male	374	38.67	66	1.171	1.022
17	left	79	female	373	23.3	66	1.181	1.434

Supplemental table S2: Individual demographic, clinical and graph theoretical data from stroke patients included in the study. Mean connectivity strength (q50) is shown for the ipsi- and contralesional hemisphere. Abbreviations: UEFM, Fugl-Meyer assessment of the upper extremity.

Q2: In general, there is not enough information in the methods to allow the study to be reproduced. For example, no details are provided about the protocol for how grip strength was assessed in the patients, was it assessed at the same time as patients were scanned?

A: We agree with the reviewer and have corrected this by adding detailed descriptions of the study protocol and clinical assessment to increase reproducibility of our study:

Exclusion criteria included contraindications for magnetic resonance imaging, aphasia or hemianopia disabling patients to give written informed consent or participate in clinical evaluation as well as presence of neurological or psychiatric comorbidities. Motor deficits were quantified by means of whole-hand grip force and the Fugl-Meyer assessment of the upper extremity (UEFM) which quantifies active movement

range and synergies of upper extremity muscles on an ordinal scale. For grip strength testing, grip force of both hands (affected and unaffected) was measured three consecutive times using a hand dynamometer (Strength JAMAR hand evaluation kit, Elite healthcare, UK) and the average of three measurements was taken for further analysis. Clinical evaluation and testing of motor deficits was performed prior to MR imaging, all participants were scanned by MRI on the same day as clinical testing.

Q 3: Although this article will be of interest to the greater stroke community, there is a lot of specialist language that could be understood only by experts in the field of graph theory. The results could be rewritten to improve the narrative and to briefly introduce and explain some of the technical concepts.

A: We thank the reviewer for this suggestion to make our manuscript more comprehensible and appealing to the larger stroke community. As such, we have restructured the graph theoretical results section in three parts capturing the main aspects of our analysis.

"We investigated three aspects of structural brain networks in association with clinical parameters to capture the integrity and topological changes of the structural connectome during the chronic stage after stroke. Therefore, we applied measures from graph theory that quantify the degree of overall connectivity, network organization (topology) and localization of pronounced network changes in specific anatomical brain areas."

In addition, we now start every section with a brief narrative introduction to guide readers through our analysis (further changes marked in the revised manuscript).

Q4: The authors hypothesised that the global and local graph parameters relate to clinical measures of motor impairment but their data do not support this hypothesis. Although they discuss possible reasons why it might be interesting (if known) if factors like hypertension, known to influence white matter connectivity, may be related to alterations in connectivity in this population.

A: We agree with the reviewer that this aspect needs to be addressed. We have added a section stating the limitations of our study:

Several limitations of our study have to be mentioned. Considering the cross sectional design of our study and lack of longitudinal data, we cannot exclude that the observed alterations in structural network integrity and topology are solely based on the localized stroke lesion. Although the observed changes are pathophysiologically plausible and reproducible in a basic network simulation model, it is conceivable that they are influenced by other premorbid risk factors for cerebrovascular diseases such as hypertension or

diabetes commonly present in stroke patients. Since we did not systematically record these potentially confounding factors, further studies applying a multivariate analysis and longitudinal design are needed to elucidate the significance of potential pre-morbid vulnerabilities of brain networks in stroke patients

Q5: Figure 6 should be introduced in the results section, it should not firstly be referred to in the discussion.

A: The figure is now mentioned first in the results section. Please note that after restructuring the results section, this is now figure 5 in the revised manuscript.

Minor points:

Q6: Sentence 2 of the abstract contains typos and should be corrected.

A: This has been corrected. In addition, the manuscript has been critically revised for grammatical and spelling errors by a native speaker.

Q7: The opening section of the discussion states that there are “indications of non-significantly reduced connection strength in the contralesional hemispheres”. If the data are not significant, they are not significant and therefore there is no evidence to suggest that connection strength is altered in the contralesional hemisphere.

A: We follow this conception and have omitted the sentence in the discussion section.

References

Bassett DS, Bullmore ET. Small-World Brain Networks Revisited. Neuroscientist 2017; 23: 499–516.

Buchanan CR, Bastin ME, Ritchie SJ, Liewald DC, Madole J, Tucker-Drob EM, et al. The effect of network thresholding and weighting on structural brain networks in the UK Biobank. bioRxiv 2019: 649418.

Cheng B, Braass H, Ganos C, Treszl A, Biermann-Ruben K, Hummel FC, et al. Altered intrahemispheric structural connectivity in Gilles de la Tourette syndrome. NeuroImage Clin 2014; 4: 174–181.

Civier O, Smith RE, Yeh C-H, Connelly A, Calamante F. Is removal of weak connections necessary for

graph-theoretical analysis of dense weighted structural connectomes from diffusion MRI? Neuroimage 2019; 194: 68–81.

Finger H, Bönstrup M, Cheng B, Messé A, Hilgetag C, Thomalla G, et al. Modeling of Large-Scale Functional Brain Networks Based on Structural Connectivity from DTI: Comparison with EEG Derived Phase Coupling Networks and Evaluation of Alternative Methods along the Modeling Path. PLOS Comput Biol 2016; 12: e1005025.

Markov NT, Ercsey-Ravasz M, Lamy C, Ribeiro Gomes AR, Magrou L, Misery P, et al. The role of long-range connections on the specificity of the macaque interareal cortical network. Proc Natl Acad Sci U S A 2013; 110: 5187–92.

Seguin C, van den Heuvel MP, Zalesky A. Navigation of brain networks. Proc Natl Acad Sci U S A 2018; 115: 6297–6302.
