# Supplementary material for: Altered topology of large-scale structural brain networks in chronic stroke
Source: Brain Commun. 2019 Oct 4;1(1):fcz020. doi: 10.1093/braincomms/fcz020 (PMC7425306; doi:10.1093/braincomms/fcz020)
Supplement: fcz020_Supplementary_Data [file Decision_original_submission.pdf › Decision_original_submission.pdf]

25-Jul-2019

Dear Dr. Cheng,

Thank you for your recent submission BRAINCOM-2019-032 entitled "Altered topology of large-scale structural brain networks in chronic stroke" to Brain Communications.

Your manuscript has been reviewed, and while the reviewers and editorial team appreciate many aspects of the paper, they raise some concerns that you will need to address in order for the paper to be considered for publication.

Therefore, I invite you to respond to the reviewer(s)' comments and revise your manuscript. The reviewer(s)’ comments are included at the bottom of this message.

In addition to the reviewers' comments, please note our manuscript formatting guidelines which can be found here:

https://academic.oup.com/braincomms/pages/General_Instructions

To revise your manuscript, click the link below or log into https://mc.manuscriptcentral.com/braincom and enter your Author Centre, where you will find your manuscript title listed under "Manuscripts with Decisions." Under "Actions," click on "Create a Revision." Your manuscript number has been appended to denote a revision.

*** PLEASE NOTE: This is a two-step process. After clicking on the link, you will be directed to a webpage to confirm. ***

https://mc.manuscriptcentral.com/braincom?URL_MASK=2e9de2c895b74500813de21865387c68

Please upload a revised manuscript with changes tracked with track changes or in coloured text.

When submitting your revised manuscript, you will be asked to respond to the reviewer(s) comments. Please be as specific as possible in your response to all of the comments.

The editorial team at Brain Communications aims to increase transparency in scientific publishing and to demonstrate the value that peer review brings to the scientific process. Please therefore consider whether you are willing to have reviewer(s)’ comments on your paper and your response published as supplementary data with the paper if it is accepted. This is entirely optional and will only be done if both the authors and reviewers agree. While submitting your revision there will be an opportunity to review or update the answer you gave previously, if necessary.

IMPORTANT: Your original files are available to you when you upload your revised manuscript. Please delete any redundant files before completing the submission.

Because we are trying to facilitate timely publication of manuscripts submitted to Brain Communications, we would appreciate your revised manuscript as soon as possible. If it is not possible for you to resubmit within 3 months, the option to submit a revision will expire, and we would then consider your revised paper as a new submission. However, if you need a little more time, please get in touch and we can extend this deadline for you.

Once again, thank you for submitting your manuscript to Brain Communications and I look forward to receiving your revision.

Yours sincerely,
Prof. Kevin Talbot
Editor, Brain Communications

Reviewer(s)' Comments to Author:

Reviewer: 1

Comments to the Author
This paper addresses a timely and interesting question, namely how structural network topology is affected by stroke. The authors use some standard approaches to investigate differences between stroke patients and controls in two small samples, while also performing some basic simulation work.

Although they are to be commended for attempting to tackle this topic, several issues need to be addressed:
- Was fiber tracking performed from a gray matter mask only? We know that this might be problematic, which is why a lot of studies use the gray/white matter rim as a seed region.
- Were the stroke lesions masked out? Was visual inspection used to check whether lesions induced strange results due to problems with the tracking algorithm? These are essential things to take into account when using diffusion imaging around lesions that may induce severe artifacts.
- What is the rationale of using 70/80% proportional thresholds? These are very high, while recent studies show that the 'correct' density of a structural network lies around 20%, but is certainly not as high as 70/80%.
- The statistical approach would be very robust in a large sample, but does bring up the question of overfitting and power issues in the context of only 17 (heterogeneous) patients being included. Can the authors comment on this issue?
- The authors use 'changes' to indicate what are in fact differences between patients and controls. Although I understand that the assumption here is that the network measured in patients reflects possible changes that have occurred as a result of stroke, this assumption should be explicitized. Also, I would recommend making clear in the discussion that it is also possible that differences in network topology between groups are (in part) due to premorbid differences, as the increasingly rich literature on vulnerability in functional/structural networks already indicate. In the context of this manuscript, it could be that people with already greater segregation of their structural network because of some preexistent variable are at greater risk for stroke. Furthermore, the lack of association with any clinical parameters also take away from the linear view that the authors present on the impact of stroke on a structural connectome.
- "No thresholding of structural connectomes was applied for the analysis of local network parameters." Why not? Why was this procedure used for global, but not local measures, which are obviously also impacted by the total number/weight of connections?
- Grammar throughout the manuscript should be critically evaluated. For instance, the second sentence of the abstract is already incorrect and thereby confusing: "stroke lesion have shown do induce alterations in functional brain connectivity and brain network topology associated with clinical severity and recovery". Another example in which the incorrect use of 'on' obscures what is meant by the authors is the second to last sentence of the abstract: "We propose that the observed network changes in our patients are best explained on the disruption of inter- and intrahemispheric, long white matter fiber tracts connecting distant brain regions." Should this be 'by'? Finally, the use of hyphens (e.g. in graph-theory, 3-T scanner, etc) should be decreased.

Reviewer: 2

Comments to the Author
Cheng and colleagues have investigated alterations in the structural connectome in the chronic response to stroke in a cohort of 17 individuals who have had focal cerebral ischemia caused by MCA stroke in comparison with an age matched control group of 21 individuals. Alterations to the post stroke connectome have been studied previously with EEG and fMRI and in the present manuscript the authors have used diffusion-weighted imaging and probabilistic fiber tracking to study the impact of focal lesions on the structural connectome. The findings are novel and will be of interest to the stroke community interested in how a focal lesion can impact on long term motor and cognitive deficits.
There are some issues that should be addressed.

1) The timepoints post stroke when the patients were scanned is not well defined, and are referred to only as the ‘chronic response’ greater than 6 months. The IQR suggests that there is a lot of variability in the time that the individuals were scanned. It would be useful to have a table (perhaps in supplemental data or an online resourse) where the exact timepoints for each patient was reported, together with grip strength, sex of patient, and some of the key connectome data ie. Connectivity

strength. It could be that stratifying the data based on recovery time post stroke may reveal trends in the data which may reveal how the connectome alters over time after a stroke (although the study is likely underpowered for this),

2) In general, there is not enough information in the methods to allow the study to be reproduced. For example, no details are provided about the protocol for how grip strength was assessed in the patients, was it assessed at the same time as patients were scanned?

3) Although this article will be of interest to the greater stroke community, there is a lot of specialist language that could be understood only by experts in the field of graph theory. The results could be rewritten to improve the narrative and to briefly introduce and explain some of the technical concepts.

4) The authors hypothesised that the global and local graph parameters relate to clinical measures of motor impairment but their data do not support this hypothesis. Although they discuss possible reasons why it might be interesting (if known) if factors like hypertension, known to influence white matter connectivity, may be related to alterations in connectivity in this population.

5) Figure 6 should be introduced in the results section, it should not firstly be referred to in the discussion.

Minor points:

Sentence 2 of the abstract contains typos and should be corrected.

The opening section of the discussion states that there are “indications of non-significantly reduced connection strength in the contralesional hemispheres”. If the data are not significant, they are not significant and therefore there is no evidence to suggest that connection strength is altered in the contralesional hemisphere.
