# Supplementary material for: Altered topology of large-scale structural brain networks in chronic stroke
Source: Brain Commun. 2019 Oct 4;1(1):fcz020. doi: 10.1093/braincomms/fcz020 (PMC7425306; doi:10.1093/braincomms/fcz020)
Supplement: fcz020_Supplementary_Data [file Decision_revision_1.pdf › Decision_revision_1.pdf]

02-Sep-2019

Dear Dr. Cheng,

It is a pleasure to accept your manuscript entitled "Altered topology of large-scale structural brain networks in chronic stroke" for publication in Brain Communications. Any comments made by the reviewer(s) are included below.

The next step is for your paper to go through “First Look”. We will contact you again shortly if any final files or formatting changes are required for publication or if we have any queries.

If no changes are needed, your submission will be transferred to our Production Department and you will hear from the Production Editor in due course.

Thank you very much for your contribution. On behalf of the Editors of Brain Communications, we look forward to your future contributions to the Journal.

Yours sincerely,

Professor Kevin Talbot
Associate Editor
Brain Communications

Reviewer(s)' Comments to Author:

Reviewer: 2

Comments to the Author
The manuscript has been well revised and now addresses both reviewers comments.
